# Supplementary material for: The DEXA-CORT trial: study protocol of a randomised placebo-controlled trial of hydrocortisone in patients with brain tumour on the prevention of neuropsychiatric adverse effects caused by perioperative dexamethasone
Source: BMJ Open. 2021 Dec 28;11(12):e054405. doi: 10.1136/bmjopen-2021-054405 (PMC8719188; doi:10.1136/bmjopen-2021-054405)
Supplement: Supplementary data [file bmjopen-2021-054405supp002.pdf]

Versie 4, 14-02-20

The

DEXA-CORT

Trial

Koning A-SCAM, et al. BMJ Open 2021; 11:e054405. doi: 10.1136/bmjopen-2021-054405

Proefpersoneninformatie voor deelname aan medisch-wetenschappelijk onderzoek

Cortisol om psychische bijwerkingen van dexamethason te voorkomen

Hydrocortison als co-medicatie om nadelige neuropsychiatrische effecten van dexamethason te voorkomen.

Inleiding

Geachte heer/mevrouw,

Wij vragen u om mee te doen aan een medisch-wetenschappelijk onderzoek.
Meedoen is vrijwillig. Om mee te doen is wel uw schriftelijke toestemming nodig. U ontvangt deze brief omdat bij u een hersentumor is gevonden waaraan u binnenkort wordt geopereerd. Voordat u beslist of u wilt meedoen aan dit onderzoek, is het belangrijk om meer te weten over het onderzoek. Lees deze informatie rustig door. Bespreek het met uw partner, vrienden of familie. Heeft u na het lezen van de informatie nog vragen? Dan kunt u terecht bij de onderzoeker of de onafhankelijk deskundige, die aan het eind van deze brief genoemd wordt. Verdere informatie over meedoen aan zo'n onderzoek staat in de bijgevoegde brochure 'Medisch-wetenschappelijk onderzoek'.

1. Algemene informatie

Dit onderzoek is opgezet door het Leids Universitair Medisch Centrum (LUMC) in samenwerking met het Erasmus MC Universitair Medisch Centrum Rotterdam (EMC), het Universitair Medisch Centrum Utrecht (UMCU) en Haaglanden Medisch Centrum (HMC). Voor dit onderzoek zijn 180 proefpersonen nodig. De medisch-ethische toetsingscommissie van het LUMC heeft dit onderzoek goedgekeurd. Algemene informatie over de toetsing van onderzoek vindt u in de brochure 'Medisch-wetenschappelijk onderzoek'.

2. Doel van het onderzoek

Het doel van dit onderzoek is uitzoeken of de psychische (geestelijke) bijwerkingen van het medicijn dexamethason verminderd kunnen worden. Dexamethason wordt standaard gebruikt bij hersenoperaties om zwelling en ontsteking te voorkomen. Echter, het is bekend dat dexamethason psychische bijwerkingen kan veroorzaken, bijvoorbeeld depressie, angst en verstoorde gedachten. Wij denken dat deze bijwerkingen worden veroorzaakt omdat dexamethason de aanmaak van het lichaamseigen hormoon cortisol (= hormoon gemaakt in de bijnier) afremt. Door nu dit lichaamseigen hormoon cortisol (= hydrocortison) in tabletvorm aan de dexamethason toe te voegen, denken wij dat deze bijwerkingen minder vaak zullen optreden.

Koning A-SCAM, *et al. BMJ Open* 2021; 11:e054405. doi: 10.1136/bmjopen-2021-054405

3. Achtergrond van het onderzoek

Dexamethason wordt door dokters aan verschillende groepen patiënten voorgeschreven. Ongeveer 1 op de 5 patiënten krijgt last van psychische bijwerkingen of slaapproblemen. Of deze klachten daadwerkelijk optreden en de ernst hiervan hangen af van de dosis en de duur van de behandeling. Omdat deze klachten zeer hinderlijk kunnen zijn en kunnen leiden tot een verminderde kwaliteit van leven, willen wij met deze studie een behandeling testen om dit soort bijwerkingen tegen te gaan. Meer gedetailleerde informatie over het onderzoek kunt u vinden in **bijlage D:** Extra achtergrond informatie.

4. Wat meedoen inhoudt

Als u meedoet, krijgt u rondom de operatie, tijdens het verblijf in het ziekenhuis twee keer daags een extra tablet toegediend naast de normale behandeling. Deze tablet bevat ofwel het lichaamseigen hormoon hydrocortison, dat bij u niet meer aangemaakt wordt, ofwel een niet werkzame stof (placebo). Tijdens de opname en tot 3 maanden erna wordt u op verschillende momenten onderzocht waarbij u vragenlijsten moet invullen die de aanwezigheid van de psychische bijwerkingen en uw algehele functioneren in kaart brengen. De testmomenten voor het onderzoek worden gekoppeld aan de zorg die u al krijgt. De studiemedicatie zult u gedurende uw opname in het ziekenhuis krijgen en misschien thuis, dit zal maximaal 15 dagen zijn, afhankelijk van hoe lang u de dexamethason moet gebruiken. In de weken na uw ontslag uit het ziekenhuis zullen de vragenlijsten aan u voorgelegd worden op de dag dat u voor controle terug verwacht wordt in het ziekenhuis. Daarnaast moet u nog op 2 andere momenten thuis vragenlijsten invullen. Omdat het laatste meetmoment 3 maanden na de operatie is gepland, is de totale duur van de studie 3 maanden. U zult in totaal ongeveer 5 tot 6 uur met het invullen van de vragenlijsten bezig zijn verspreid over 3 maanden.

Geschiktheidsonderzoek

Eerst bepalen we of u kunt meedoen. Dit gebeurt aan de hand van een aantal voorwaarden waaraan u moet voldoen, daarbij zijn onder andere uw leeftijd en het gebruik van andere medicijnen van belang. Wanneer u besluit mee te doen aan de studie, wordt aan u gevraagd of u een toestemmingsformulier wilt tekenen. Na ondertekening wordt uw deelname aan de studie officieel. De onderzoeker zal dan vragen naar uw medische geschiedenis, zoals psychische achtergrond, medicatie en klachten.

Behandeling

We behandelen u met de studiemedicatie even lang als u volgens de standaard zorg het medicijn dexamethason toegediend krijgt. De ene helft van de mensen die deelnemen aan dit onderzoek krijgt het lichaamseigen hormoon hydrocortison, de andere helft krijgt een tablet waarin geen werkzame stof zit (placebo). Loting bepaalt welke behandeling u krijgt. U, de onderzoeker, verpleegkundige en de arts weten niet in welke groep u zit. Als het voor uw gezondheid belangrijk is, kan dit wel worden opgezocht. Algemene informatie hierover vindt u in de brochure ‘Medisch-wetenschappelijk onderzoek’.

Koning A-SCAM, *et al. BMJ Open* 2021; 11:e054405. doi: 10.1136/bmjopen-2021-054405

Bezoeken en metingen

Voor deze studie zijn geen extra bezoeken aan het ziekenhuis nodig. De testen die uitgevoerd moeten worden voor het onderzoek, worden gepland op de dagen dat u toch al in het ziekenhuis bent.

Er zal dan het volgende gebeuren:
- Bij het eerste gesprek vragen we naar uw medische geschiedenis (psychisch en medicatie) en vragen naar klachten.
- We laten u op verschillende momenten vragenlijsten invullen over uw slaapkwaliteit, kwaliteit van leven, hoe u zichzelf voelt en uw psychologisch welzijn.
- We meten op verschillende momenten uw psychisch welzijn via een interview met één van de onderzoekers. Ook zullen de verpleegkundigen uw welzijn in de gaten houden.
- We laten u verschillende korte testen voor geheugen, aandacht en taal uitvoeren.
- We laten u een horloge dragen dat de slaapkwaliteit kan meten.

Veel van de vragenlijsten kunt u (digitaal) thuis invullen. In **bijlage C:** Schema onderzoek handelingen staat welke metingen er bij elk van de bezoeken plaatsvinden. En hoeveel tijd dit ongeveer in beslag zal nemen.

Tijdens het onderzoek kan aan u gevraagd worden om bloed af te staan. In dit bloed willen wij kijken naar genetische variatie. Daarmee onderzoeken we of de reactie op medicatie mede bepaald wordt door de samenstelling van uw DNA.

Daarnaast kan tijdens het onderzoek gevraagd worden of er een stukje van het verwijderde tumor- of hersenweefsel bewaard mag blijven voor nader onderzoek. In dit weefsel willen wij de werking van de hydrocortison onderzoeken.

Het buisje bloed en stukje van het verwijderde weefsel wordt eerst opgeslagen in de lokale onderzoeksinstelling en pas onderzocht nadat de studie is afgerond. Onderzoek naar het lichaamsmateriaal wordt in het LUMC uitgevoerd. Indien u niet in het desbetreffende centrum onder behandeling bent geweest, wordt uw lichaamsmateriaal naar het LUMC gebracht, opgeslagen en onderzocht. Meer gedetailleerde informatie over dit deel van het onderzoek kunt u vinden in **bijlage D:** Extra achtergrond informatie. Als u liever niet aan dit deel van het onderzoek wilt meedoen, kunt u dit aangeven in het toestemmingsformulier.

5. Wat wordt er van u verwacht

Om het onderzoek goed te laten verlopen is het belangrijk dat u zich aan de volgende afspraken houdt.

De afspraken zijn dat u:
- de studiemedicatie inneemt volgens de uitleg.
- niet meedoet aan nog een ander medisch-wetenschappelijk onderzoek.
- afspraken voor bezoeken nakomt.

Het is belangrijk dat u contact opneemt met de onderzoeker:

- voordat u andere geneesmiddelen gaat gebruiken. Ook als dat homeopathische geneesmiddelen, natuurgeneesmiddelen, vitaminen en/of geneesmiddelen van de drogist zijn.
- als u in de maanden na de operatie in een ziekenhuis wordt opgenomen of behandeld.
- als u plotseling gezondheidsklachten krijgt.
- als u niet meer wilt meedoen aan het onderzoek.
- als uw contactgegevens wijzigen.

Partner, familielid of andere naaste die voor u zorgt
Wij willen voor het onderzoek ook uw partner (of familielid of andere naaste) vragen om drie keer een vragenlijst in te vullen over uw psychisch welzijn.

6. Mogelijke bijwerkingen

Wij kunnen van te voren niet voorspellen of u last gaat krijgen van de dexamethason. Wij verwachten geen extra bijwerkingen door de studiemedicatie, omdat de studiemedicatie nagenoeg gelijk is aan de hoeveelheid lichaamseigen cortisol die normaal geproduceerd zou worden. We vullen een tekort aan. Meedoen aan de studie zal daarom naar onze inschatting geen extra risico's met zich meebrengen naast de al bestaande risico's van uw behandeling.

7. Mogelijke voor- en nadelen

Het is belangrijk dat u de mogelijke voor- en nadelen goed afweegt voordat u besluit mee te doen.

Voordelen van deelname:

- De studiemedicatie zou de mogelijke psychische bijwerkingen van dexamethason kunnen verminderen. Vooral gevoelens van angst en onrust zouden minder kunnen optreden, maar zeker is dat niet.
- De studiemedicatie zou uw slaapkwaliteit kunnen verbeteren, maar zeker is dat niet.

Nadelen van deelname:

- Het is belangrijk dat u de medicijnen met regelmaat en volgens de instructies inneemt.
- De testmomenten waarbij vragenlijsten ingevuld moeten worden kosten tijd en kunnen vermoeiend zijn.
- U heeft afspraken waaraan u zich moet houden.
- De vragenlijsten kunnen confronterend zijn, omdat ze vragen naar hoe u zich voelt en hoe het in het dagelijks leven met u gaat.
- Tijdens de testen voor het geheugen, de aandacht en de taal komen uw sterke maar ook uw minder sterke functies aan het licht. Dit kan soms confronterend zijn.

Koning A-SCAM, *et al. BMJ Open* 2021; 11:e054405. doi: 10.1136/bmjopen-2021-054405

8. Als u niet wilt meedoen of wilt stoppen met het onderzoek

U beslist zelf of u meedoet aan het onderzoek. Deelname is vrijwillig. Als u niet wilt meedoen, hoeft u verder niets te doen. U hoeft niets te tekenen. U hoeft ook niet te zeggen waarom u niet wilt meedoen. U krijgt gewoon de behandeling die u anders ook zou krijgen. Er verandert niets.

Als u wel meedoet, kunt u zich altijd bedenken en toch stoppen, ook tijdens het onderzoek. U wordt dan weer op de gebruikelijke manier verder behandeld. U hoeft niet te zeggen waarom u stopt. Wel moet u dit direct melden aan de onderzoeker.
De gegevens die tot dat moment zijn verzameld, worden gebruikt voor het onderzoek. Als u wilt, en indien van toepassing, kan verzameld lichaamsmateriaal worden vernietigd. Dit moet u wel zelf aangeven. Wanneer u dit niet meldt, wordt het gebruikt voor het onderzoek.

Als er nieuwe informatie over het onderzoek is die belangrijk voor u is, laat de onderzoeker dit aan u weten. U wordt dan gevraagd of u blijft meedoen.

9. Einde van het onderzoek

Uw deelname aan het onderzoek stopt als

- alle testmomenten voorbij zijn en de vragenlijsten zijn ingeleverd
- u zelf kiest om te stoppen
- de arts het beter voor u vindt om te stoppen
- de overheid of de beoordelende medisch-ethische toetsingscommissie, besluit om het onderzoek te stoppen.

Het hele onderzoek is afgelopen als alle deelnemers klaar zijn.
De medicatie die u gebruikt heeft bij het onderzoek, is niet beschikbaar na afloop van het onderzoek.

Na het verwerken van alle gegevens informeert de onderzoeker u over de belangrijkste uitkomsten van het onderzoek. Dit gebeurt ongeveer 2 tot 3 jaar na uw deelname.
De onderzoeker kan u ook vertellen welke behandeling u heeft gehad/in welke groep u zat. Als u dit niet wilt, dan kunt u dit tegen de onderzoeker zeggen. De onderzoeker mag het u dan niet vertellen.

10. Gebruik en bewaren van uw gegevens en lichaamsmateriaal

Voor dit onderzoek worden uw persoonsgegevens en, indien van toepassing, lichaamsmateriaal verzameld, gebruikt en bewaard. Het gaat om gegevens zoals uw naam, adres, geboortedatum en om gegevens over uw gezondheid. Het verzamelen, gebruiken en bewaren van uw gegevens en mogelijk uw lichaamsmateriaal is nodig om de vragen die in dit onderzoek worden gesteld te kunnen beantwoorden en de resultaten te kunnen publiceren. Wij vragen voor het gebruik van uw gegevens en mogelijk lichaamsmateriaal uw toestemming.

Vertrouwelijkheid van uw gegevens en mogelijk lichaamsmateriaal

Om uw privacy te beschermen geven we een code aan uw gegevens en, indien van toepassing, uw lichaamsmateriaal. Uw naam en andere gegevens die u direct kunnen identificeren worden daarbij weggelaten. Alleen met de sleutel van de code zijn gegevens tot u te herleiden. De sleutel van de code blijft opgeborgen in de lokale onderzoeksinstelling. Ook in rapporten en publicaties over het onderzoek zijn de gegevens niet tot u te herleiden.

Toegang tot uw gegevens voor controle

Sommige personen kunnen op de onderzoekslocatie toegang krijgen tot al uw gegevens. Ook tot de gegevens zonder code. Dit is nodig om te kunnen controleren of het onderzoek goed en betrouwbaar is uitgevoerd. Personen die ter controle inzage kunnen krijgen in uw gegevens zijn: de commissie die de veiligheid van het onderzoek in de gaten houdt, monitors die voor het LUMC werken en de Inspectie Gezondheidszorg en Jeugd. Zij houden uw gegevens geheim. Wij vragen u voor deze inzage toestemming te geven.

Bewaartermijn gegevens en lichaamsmateriaal

Uw gegevens moeten 15 jaar worden bewaard op de onderzoekslocatie. Indien van toepassing, uw lichaamsmateriaal wordt voor onbepaalde tijd bewaard in de lokale onderzoeksinstelling. Hiermee willen wij na deze studie nog bepalingen doen. Onderzoek naar het lichaamsmateriaal wordt in het LUMC uitgevoerd. Indien u niet in het desbetreffende centrum onder behandeling bent geweest, wordt uw lichaamsmateriaal voor de onderzoeksbepalingen naar het LUMC gebracht, opgeslagen en onderzocht. De gegevens en lichaamsmateriaal die vanuit een deelnemend ziekenhuis naar het LUMC worden gestuurd bevatten alleen de code, en niet uw naam of andere gegevens waarmee u kunt worden geïdentificeerd.

Intrekken toestemming

U kunt uw toestemming voor gebruik van uw persoonsgegevens en mogelijk lichaamsmateriaal altijd weer intrekken. Dit geldt voor dit onderzoek en ook voor het bewaren en het gebruik voor het toekomstige onderzoek. De onderzoeksgegevens die zijn verzameld tot het moment dat u uw toestemming intrekt worden nog wel gebruikt in het onderzoek. Uw lichaamsmateriaal wordt alleen op uw verzoek vernietigd. Als er al metingen met dat lichaamsmateriaal zijn gedaan, dan worden die gegevens nog wel gebruikt.

Meer informatie over uw rechten bij verwerking van gegevens

Voor algemene informatie over uw rechten bij verwerking van uw persoonsgegevens kunt u de website van de Autoriteit Persoonsgegevens raadplegen.

Bij vragen over uw rechten kunt u contact opnemen met de verantwoordelijke voor de verwerking van uw persoonsgegevens. Voor dit onderzoek is dat: het LUMC. Zie **bijlage A:** Contactgegevens.

Bij vragen of klachten over de verwerking van uw persoonsgegevens raden we u aan eerst contact op te nemen met de onderzoekslocatie. U kunt contact opnemen met de Functionaris voor de Gegevensbescherming van de instelling of de Autoriteit Persoonsgegevens. Zie **bijlage A:** Contactgegevens.

Registratie van het onderzoek

Informatie over dit onderzoek is ook opgenomen in een overzicht van medisch-wetenschappelijke onderzoeken, namelijk in het Nederlands Trial Register (http://www.trialregister.nl). Daarin zijn geen gegevens opgenomen die naar u herleidbaar zijn. Na het onderzoek kan de website een samenvatting van de resultaten van dit onderzoek tonen. U vindt dit onderzoek onder de naam: DEXA-CORT.

11. Verzekering voor proefpersonen

Voor iedereen die meedoet aan dit onderzoek is een verzekering afgesloten. De verzekering dekt schade door het onderzoek. Niet alle schade is gedekt. In **bijlage B:** Informatie over de verzekering, vindt u meer informatie over de verzekering en de uitzonderingen. Daar staat ook aan wie u de schade kunt melden.

12. Informeren huisarts en behandelend specialist

Wij zullen uw huisarts niet informeren dat u meedoet aan het onderzoek. Als u dit liever wel wilt, kunt u altijd zelf contact opnemen met uw huisarts om te vertellen dat u aan de studie meedoet. Bij vragen over uw medische geschiedenis of medicijngebruik, nemen we wel contact op met uw huisarts of behandeld arts. U kunt niet deelnemen aan het onderzoek als u geen huisarts heeft. Uw behandelend arts wordt wel geïnformeerd over uw deelname via uw dossier.

13. Geen vergoeding voor meedoen

De studiemedicatie voor het onderzoek kost u niets. U wordt niet betaald voor het meedoen aan dit onderzoek. Studie afspraken worden gepland op dagen dat u al een afspraak in het ziekenhuis heeft. Wanneer u voor het onderzoek extra naar het ziekenhuis moet komen, zullen uw reiskosten worden vergoed.

14. Heeft u vragen?

Bij vragen kunt u contact opnemen met de onderzoeker of het onderzoeksteam. Voor onafhankelijk advies over meedoen aan dit onderzoek kunt u terecht bij de onafhankelijke arts; dr. Stijn W. Genders. Hij weet veel over het onderzoek, maar hij is niet betrokken bij deze studie. Indien u klachten heeft over het onderzoek, kunt u dit bespreken met de onderzoeker of uw behandelend arts. Wilt u dit liever niet, dan kunt u zich wenden tot de klachtenfunctionaris van uw ziekenhuis. Alle gegevens vindt u in **bijlage A**: Contactgegevens.

15. Ondertekening toestemmingsformulier

Wanneer u voldoende bedenktijd van minimaal 1 week en maximaal 2 weken heeft gehad, wordt u gevraagd te beslissen over deelname aan dit onderzoek. Indien u toestemming geeft, zullen wij u vragen deze op de bijbehorende toestemmingsverklaring schriftelijk te bevestigen. Door uw schriftelijke toestemming geeft u aan dat u de informatie heeft begrepen en instemt met deelname aan het onderzoek. Zowel uzelf als de onderzoeker ontvangen een getekende versie van deze toestemmingsverklaring.

Dank voor uw aandacht.

Koning A-SCAM, *et al. BMJ Open* 2021; 11:e054405. doi: 10.1136/bmjopen-2021-054405

16. Bijlagen bij deze informatie

A. Contactgegevens
B. Informatie over de verzekering
C. Schema onderzoek handelingen
D. Extra achtergrond informatie
E. Toestemmingsformulier
F. Toestemmingsformulier voor partner (of familielid of andere naaste)
G. Brochure 'Medisch-wetenschappelijk onderzoek. Algemene informatie voor de proefpersoon'

Koning A-SCAM, *et al. BMJ Open* 2021; 11:e054405. doi: 10.1136/bmjopen-2021-054405

Versie 4, 14-02-20

Bijlage A: Contactgegevens

Coördinerend onderzoeker	Onafhankelijk arts
Anne-Sophie Koning, MSc	Dr. Stijn W. Genders
dexonderzoek@lumc.nl	oog.stafsecretariaat@lumc.nl
+31-71-5265303 / +31-71-5263082	+31-71-5262374
**LUMC contact gegevens**	
Dr. Wouter R. van Furth	
neurochirurgie@lumc.nl	
+31-71-5262109	
**Functionaris voor de Gegevensbescherming LUMC**	
Wanneer u vragen heeft over de bescherming van uw privacy kunt u contact opnemen de functionarissen gegevensbescherming van het LUMC (FG) via infoavg@lumc.nl	
**Klachtenfunctionaris van het LUMC**	
Bij klachten kunt u zich melden bij het patiëntenservicebureau in het LUMC, locatie H2-11 (routenummer 473, tegenover het Leidseplein). Hier kunt u melding maken van uw onvrede en het klachtenformulier invullen. Het patiëntenservicebureau informeert u zo snel mogelijk over een mogelijke oplossing en kan eventueel de klachtenfunctionaris inschakelen. U kunt ook het klachtenformulier digitaal invullen. Zie website LUMC pagina klacht indienen<br><br>**Contactgegevens patiëntenservicebureau**<br>LUMC Patiëntenservicebureau<br>Postbus 9600<br>2300 RC Leiden<br>Telefoon: +31 71-5262989	

Koning A-SCAM, et al. BMJ Open 2021; 11:e054405. doi: 10.1136/bmjopen-2021-054405

Bijlage B: Informatie over de verzekering

Voor iedereen die meedoet aan dit onderzoek heeft het LUMC een verzekering afgesloten. De verzekering dekt schade door deelname aan het onderzoek. Dit geldt voor schade tijdens het onderzoek of binnen vier jaar na het einde van uw deelname aan het onderzoek. Schade moet u binnen die vier jaar aan de verzekeraar hebben gemeld.

De verzekering dekt niet alle schade. Onderaan deze tekst staat in het kort welke schade niet wordt gedekt.
Deze bepalingen staan in het Besluit verplichte verzekering bij medisch-wetenschappelijk onderzoek met mensen. Dit besluit staat op www.ccmo.nl, de website van de Centrale Commissie Mensgebonden Onderzoek (zie 'Bibliotheek' en dan 'Wet- en regelgeving').

Bij schade kunt u direct contact leggen met de verzekeraar.

De verzekeraar van het onderzoek is:	
Naam:	Centramed
Adres:	Maria Montessorilaan 9, 2719 DB Zoetermeer
Telefoonnummer:	070-3017070
E-mail:	info@centramed.nl
Polisnummer:	624.530.305

De verzekering biedt een dekking van € 650.000,- per proefpersoon en € 5.000.000,- voor het hele onderzoek (en € 7.500.000,- per jaar voor alle onderzoeken van het Leids Universitair Medisch Centrum).

De verzekering dekt de volgende schade **niet**:

- schade door een risico waarover u in de schriftelijke informatie bent ingelicht. Dit geldt niet als het risico zich ernstiger voordoet dan was voorzien of als het risico heel onwaarschijnlijk was;
- schade aan uw gezondheid die ook zou zijn ontstaan als u niet aan het onderzoek had meegedaan;
- schade door het niet (volledig) opvolgen van aanwijzingen of instructies;
- schade aan uw nakomelingen, als gevolg van een negatief effect van het onderzoek op u of uw nakomelingen;
- schade door een bestaande behandelmethode bij onderzoek naar bestaande behandelmethoden.

Bijlage C: Schema onderzoek handelingen

Voor deze studie zijn geen extra bezoeken aan het ziekenhuis nodig. De testen die uitgevoerd moeten worden voor het onderzoek, worden gepland op de dagen dat u in het ziekenhuis bent.

Poli bezoek

Volgens reguliere zorg wordt er een poli afspraak gepland met de neurochirurg. Op de dag van deze poli afspraak willen wij u vragen 1 vragenlijst in te vullen, 1 interview af te laten nemen en een aantal testen voor het geheugen, de aandacht en de taal uit te voeren. Indien mogelijk, ook 1 vragenlijst voor uw partner (of familielid of andere naaste). Bij patiënten met een linkszijdige hersentumor willen wij nog een extra taalscreening doen. In totaal zal dit studiemoment ongeveer 80 (of 70) minuten duren.
Verder zullen wij via de email 5 vragenlijsten naar u sturen, die u thuis kunt invullen. Het invullen van alle vragenlijsten zal in totaal ongeveer 45 minuten in beslag nemen.

Dagen op verpleegafdeling

Op de dag van opname en op de dag van de operatie hoeft u niks te doen. Na de operatie zult u een aantal dagen in het ziekenhuis verblijven volgens reguliere zorg. Het aantal dagen dat u in het ziekenhuis ligt is afhankelijk van het oordeel van de artsen. Dit wordt volgens reguliere zorg bepaald. Op de dagen dat u op de verpleegafdeling verblijft, vragen wij u steeds 1 korte vragenlijst in te vullen. Ook vragen wij u een horloge te dragen waarmee we uw activiteit en slaap kwaliteit kunnen meten. Daarnaast willen wij u vragen in een slaapdagboek kort aan te geven hoe u geslapen heeft. De tijd die u deze dagen in het ziekenhuis kwijt bent aan het onderzoek is maximaal 10 minuten. Gedurende uw opname in het ziekenhuis zullen verpleegkundigen u psychisch welzijn in de gaten houden.

Dag van ontslag

Op de dag dat u naar huis mag, willen wij een interview bij u afnemen, vragen wij u 2 vragenlijsten in te vullen en we zullen een korte screening voor het geheugen, de aandacht en de taal uitvoeren. Ook willen wij uw partner (of familielid of andere naaste) vragen 1 vragenlijst in te vullen. Bij patiënten met een linkszijdige hersentumor willen wij nog een extra taalscreening doen. Dit studiemoment zal ongeveer 55 (of 45) minuten duren.

Na operatie

Ongeveer 2 weken na de operatie willen wij u vragen 4 vragenlijsten in te vullen. Dit kunt u gewoon thuis doen en het invullen van alle vragenlijsten zal in totaal ongeveer 30 minuten duren.
Rond 5 tot 8 weken na de operatie, wordt er volgens reguliere zorg een afspraak in het ziekenhuis gepland. Op deze dag in het ziekenhuis willen wij nog 1 keer het interview bij u afnemen, vragen wij u nog 2 vragenlijsten in te vullen en een aantal testen voor het geheugen, de aandacht en de taal uit te voeren. Indien mogelijk, ook 1 vragenlijst voor uw partner (of familielid of andere naaste). Dit studiemoment zal ongeveer 80 minuten duren.

Koning A-SCAM, *et al. BMJ Open* 2021; 11:e054405. doi: 10.1136/bmjopen-2021-054405

Ongeveer 3 maanden na operatie willen wij u vragen 5 vragenlijsten in te vullen. Dit kunt u gewoon thuis doen en het invullen van alle vragenlijsten zal in totaal ongeveer 45 minuten duren.

In totaal zal de studie ongeveer 5 tot 6 uur van u tijd kosten verspreid over 3 maanden.

**Studiemoment**	**Waar**	**Hoe lang**
Preoperatief	Poli bezoek	70 (tot 80) minuten
	Thuis	45 minuten
Gedurende ziekenhuisopname	Op de afdeling	30 minuten
Dag van ontslag	Op de afdeling	45 (tot 55) minuten
2 weken postoperatief	Thuis	30 minuten
5 – 8 weken postoperatief	Ziekenhuis	80 minuten
3 maanden postoperatief	Thuis	45 minuten
**Totaal**		**5 – 6 uur**

Bijlage D – Extra achtergrond informatie

Ons lichaam maakt het hormoon cortisol. Cortisol beïnvloedt veel dingen, zoals onze stemming en onze slaap. Cortisol wordt gemaakt in de bijnieren, die aangezet worden door een ander hormoon adrenocorticotroop hormoon (ACTH). Dat ACTH komt uit de hypofyse, een orgaan onder de hersenen. Als er genoeg cortisol is, krijgt de hypofyse een seintje om te stoppen met het maken van hormonen, waardoor de bijnieren dan ook geen cortisol meer maken.

Het medicijn dexamethason lijkt heel erg veel op cortisol dat het lichaam zelf kan maken. Maar het is veel sterker dan het lichaamseigen cortisol. Een kleine dosis dexamethason kan er al voor zorgen dat de hypofyse geen hormoon meer maakt. Daardoor stoppen de bijnieren met cortisol maken. Dus een bijwerking bij behandeling met dexamethason is dat ons eigen lichaam geen cortisol meer maakt.

Er is één groot verschil tussen cortisol en dexamethason. Dexamethason werkt via één aangrijpingspunt. Dat is via de glucocorticoïd receptor (GR). Cortisol werkt op twéé aangrijpingspunten: via de GR, maar ook via de mineralocorticoïd receptor (MR). Cortisol activeert dus MR en GR. Dexamethason activeert alleen de GR. Omdat ons lichaam na dexamethason geen cortisol meer maakt, kan cortisol ook niet meer de MR activeren. Het resultaat is dat alleen de GR actief is, terwijl de MR leeg blijft. Wij denken dat de bijwerkingen na dexamethason komen doordat de MR leeg is, omdat de werking van cortisol in de hersenen van de MR afhangt.

In deze studie voegen we cortisol toe aan de behandeling met dexamethason (als pilletje heeft cortisol de naam 'hydrocortison' gekregen). De hydrocortison kan binden aan de MR. Op deze manier hopen wij de normale werking van de MR weer terug te krijgen. En hopen we dat de bijwerkingen zullen verminderen.

Tijdens het onderzoek kan gevraagd worden of er bloed van u mag worden afgenomen en bewaard mag worden voor verder onderzoek. In het bloed willen wij kijken welk type van MR u heeft. Dit kunnen wij onderzoeken door in het DNA naar het gen van de MR te kijken. Het is namelijk bekend dat een bepaalde variant van het MR gen een lager risico geeft op het krijgen van depressie. Wij willen in dit onderzoek kijken of dit een onderliggende reden kan zijn voor de klachten die sommige patiënten van de dexamethason krijgen.

Met het weefsel dat van het verwijderde tumorweefsel gehaald wordt, willen wij de werking van de hydrocortison onderzoeken. Met de toegevoegde hydrocortison verwachten wij dat de MR weer actief wordt. Dat willen wij in dit weefsel meten.

Koning A-SCAM, *et al. BMJ Open* 2021; 11:e054405. doi: 10.1136/bmjopen-2021-054405

Versie 4, 14-02-20

Bijlage E: Toestemmingsformulier proefpersoon

Cortisol om psychische bijwerkingen van dexamethason te voorkomen

- Ik heb de informatiebrief gelezen. Ook kon ik vragen stellen. Mijn vragen zijn voldoende beantwoord. Ik had genoeg tijd om te beslissen of ik meedoe.

- Ik weet dat meedoen vrijwillig is. Ook weet ik dat ik op ieder moment kan beslissen om toch niet mee te doen of te stoppen met het onderzoek. Daarvoor hoef ik geen reden te geven.

- Ik geef toestemming voor het opvragen van informatie bij mijn huisarts/specialist(en) die mij behandelt indien er vragen zijn over uw medische geschiedenis of medicijngebruik.

- Ik weet dat voor de controle van het onderzoek sommige mensen toegang tot al mijn gegevens kunnen krijgen. Die mensen staan vermeld in deze informatiebrief. Ik geef toestemming voor die inzage door deze personen.

- Ik weet dat mijn partner (of familielid of andere naaste) ook een aantal keer een vragenlijst moet invullen en hij/zij gaat hiermee akkoord.

- Ik geef toestemming voor het verzamelen en gebruiken van mijn gegevens voor de beantwoording van de onderzoeksvraag in dit onderzoek.

- Ik geef □ **wel**
  □ **geen**
  toestemming voor bloedafname en dit te bewaren en te gebruiken voor toekomstig onderzoek op het gebied van onderzoek beschreven in de informatiebrief.

- Ik geef □ **wel**
  □ **geen**
  toestemming voor het bewaren van een stukje weefsel van het verwijderde tumorweefsel en dit te bewaren en te gebruiken voor toekomstig onderzoek op het gebied van onderzoek beschreven in de informatiebrief.

Koning A-SCAM, *et al. BMJ Open* 2021; 11:e054405. doi: 10.1136/bmjopen-2021-054405

Versie 4, 14-02-20

- Ik geef □ **wel**
  □ **geen**
  toestemming om mijn persoonsgegevens en lichaamsmateriaal langer te bewaren en te gebruiken voor toekomstig onderzoek op het gebied van onderzoek beschreven in de informatiebrief.

- Ik wil □ **wel**
  □ **niet**
  geïnformeerd worden over welke behandeling ik heb gehad/in welke groep ik zat.

- Ik wil meedoen aan dit onderzoek.

Naam proefpersoon: __________________

Handtekening:_______________________ Datum : __ / __ / __

Ik verklaar dat ik deze proefpersoon volledig heb geïnformeerd over het genoemde onderzoek.

Als er tijdens het onderzoek informatie bekend wordt die de toestemming van de proefpersoon zou kunnen beïnvloeden, dan breng ik hem/haar daarvan tijdig op de hoogte.

Naam onderzoeker (of diens vertegenwoordiger):______________________________

Handtekening:______________________ Datum: __ / __ / __

Koning A-SCAM, *et al. BMJ Open* 2021; 11:e054405. doi: 10.1136/bmjopen-2021-054405

Versie 4, 14-02-20

Bijlage F: Toestemmingsformulier voor partner (of familielid of andere naaste)

Cortisol om psychische bijwerkingen van dexamethason te voorkomen

- Ik ben op de hoogte dat mijn partner (of familielid of andere naaste) meedoet met het onderzoek. Ook kon ik vragen stellen. Mijn vragen zijn voldoende beantwoord. Ik had genoeg tijd om te beslissen of ik voor de studie 3x een vragenlijst wil invullen over mijn partner (of familielid of andere naaste).

- Ik weet dat meedoen vrijwillig is. Ook weet ik dat ik op ieder moment kan beslissen om toch niet mee te doen of te stoppen met het onderzoek. Daarvoor hoef ik geen reden te geven.

- Ik wil meedoen aan dit onderzoek.

Naam partner (of familielid of andere naaste):______________

Handtekening:_______________________ Datum : __ / __ / __

Ik verklaar dat ik de partner (of familielid of andere naaste) volledig heb geïnformeerd over het genoemde onderzoek.

Als er tijdens het onderzoek informatie bekend wordt die de toestemming van de partner (of familielid of andere naaste) zou kunnen beïnvloeden, dan breng ik hem/haar daarvan tijdig op de hoogte.

Naam onderzoeker (of diens vertegenwoordiger):______________________________

Handtekening:______________________ Datum: __ / __ / __

Koning A-SCAM, *et al. BMJ Open* 2021; 11:e054405. doi: 10.1136/bmjopen-2021-054405
