# Supplementary material for: The DEXA-CORT trial: study protocol of a randomised placebo-controlled trial of hydrocortisone in patients with brain tumour on the prevention of neuropsychiatric adverse effects caused by perioperative dexamethasone
Source: BMJ Open. 2021 Dec 28;11(12):e054405. doi: 10.1136/bmjopen-2021-054405 (PMC8719188; doi:10.1136/bmjopen-2021-054405)
Supplement: Supplementary data [file bmjopen-2021-054405supp003.pdf]

Version 4, 14-02-20

The

DEXA-CORT

Trial

Koning A-SCAM, *et al. BMJ Open* 2021; 11:e054405. doi: 10.1136/bmjopen-2021-054405

Version 4, 14-02-20

Study information for participation in medical research

Cortisol to prevent psychiatric adverse effects of dexamethasone

Hydrocortisone as co-medication to prevent adverse neuropsychiatric effects of dexamethasone

Introduction

Dear Sir/Madam,

You are kindly requested to take part in a medical scientific study. We have contacted you because you will undergo surgery for a brain tumor. Participation is voluntarily and requires your written consent. Before you decide whether you want to participate in this study, you will be given an explanation about what the study involves. Please read this information carefully and discuss it with your partner, friends and/or family. If you have any questions you can contact the (coordinating) investigator or independent expert for an explanation. General information about participating in a study can be found in the enclosed general brochure on medical research.

1. General information

This research was set up by the Leiden University Medical Center (LUMC) in collaboration with the Erasmus MC University Medical Center Rotterdam (EMC), the University Medical Center Utrecht (UMCU) and Haaglanden Medical Center (HMC). 180 participants are needed for this study. The medical ethics review committee of the LUMC has approved this research. General information about the assessment of research can be found in the brochure 'Medical scientific research'.

2. Purpose of the study

The aim of this study is to find out whether the psychological (mental) side effects of the drug dexamethasone can be reduced. Dexamethasone is commonly used in brain surgery to prevent swelling and inflammation. However, it is known that dexamethasone can cause psychological side effects, for example depression, anxiety and disturbed thoughts. We think that these side effects are caused because dexamethasone inhibits the production of the body's own hormone cortisol (= hormone made in the adrenal gland). By now adding this body's hormone cortisol (= hydrocortisone) in tablet form to the dexamethasone, we think that these side effects will occur less often.

3. Background of the study

Dexamethasone is prescribed by doctors to different groups of patients. About 1 in 5 patients suffer from psychological side effects or sleeping problems. Whether these complaints actually

Koning A-SCAM, *et al. BMJ Open* 2021; 11:e054405. doi: 10.1136/bmjopen-2021-054405

occur and their severity may depend on the dose and the duration of the treatment. Because these complaints can be very bothersome and can lead to a reduced quality of life, we want to use this study to test a treatment to counteract these kinds of side effects. More detailed information about the study can be found in **Appendix** D: Additional background information.

4. What does participation involve?

If you participate, you will be given an extra tablet twice a day in addition to the normal treatment during the hospital stay. This tablet contains either the body's own hormone hydrocortisone, which you no longer produce yourself, or an inactive substance (placebo). During the admission and up to 3 months afterwards, you will be examined at various times during which you have to fill in questionnaires that map the presence of the psychological side effects and your overall functioning. The test moments for the study are coinciding with standard appointments at the hospital. You will receive the study medication during your hospital stay and perhaps at home, this will be up to 15 days, depending on how long you need to take the dexamethasone. In the weeks following your discharge from hospital, the questionnaires will be presented to you on the day you are expected to return to the hospital for a check-up. In addition, you have to complete questionnaires at home at 2 other times. Because the last measurement moment is planned 3 months after surgery, the total duration of the study is 3 months. You will spend a total of about 5 to 6 hours filling in the questionnaires spread over 3 months.

Screening

If you are interested in participating we first will evaluate whether you may participate. This is determined on the basis of a number of conditions that you must meet, including your age and the use of other medicines. When you decide to participate in the study, you will be asked to sign a consent form. After signing, your participation in the study becomes official. The researcher will then ask about your medical history, such as psychological background, medication and complaints.

Treatment

We will treat you with the study medication for as long as you are administered the drug dexamethasone according to standard care. Half of the people participating in this study will be given the body's own hormone hydrocortisone, the other half will be given a tablet that does not contain an active ingredient (placebo). The treatment you will receive is determined by draw. You, the researcher, nurse and doctor do not know which group you are in. If it is important for your health, this can be looked up. General information about this can be found in the brochure 'Medical-scientific research'.

Visits and measurements

No additional hospital visits are required for this study. The tests to be performed for the examination are scheduled on the days that you are already in the hospital.

The following will then happen:

- At the first meeting we ask about your medical history (psychological and medication) and complaints.
- We let you fill in questionnaires at various times about your sleep quality, quality of life, how you feel and your psychological well-being.
- We measure your psychological well-being at various times through an interview with one of the researchers. The nurses will also monitor your well-being.
- We let you perform several short tests for memory, attention and language.
- We let you wear a watch that can measure sleep quality.

You can complete many of the questionnaires (digitally) at home. **Appendix C**: Schedule of investigation actions, lists the measurements that take place during each of the visits. And how long this will approximately take.

During the study, you may be asked to donate a tube of blood. In this blood sample we want to look at genetic variation. We use this to investigate whether the response to medication is partly determined by the composition of your DNA.

In addition, you may be asked whether a piece of the removed tumor tissue may be kept for further examination. We want to investigate the effect of the hydrocortisone in this tissue.

The samples of blood and/or tissue are first stored at the local study facility and examined only after the study is completed. Research on the body material is carried out in the LUMC. If you have not been treated in this center, your body material will be taken to the LUMC, stored and examined. More detailed information about this part of the study can be found in **Appendix D**: Additional background information. If you prefer not to participate in this part of the study, you can indicate this in the consent form.

5. What is expected of you?

In order for the study to run smoothly, it is important that you adhere to the following agreements.

The agreements are that you:

- Take the study medication according to the instructions.
- Do not participate in any other medical scientific research.
- Fulfill appointments for study visits.

It is important that you contact the researcher:

- before taking any other medicines. Even if they are homeopathic medicines, natural medicines, vitamins and/or medicines from the drugstore.
- if you are hospitalized or treated in the months following the operation.
- if you suddenly develop health problems.
- if you no longer wish to participate in the study.
- if your contact details change.

Partner, family member or other close relative

Before the study, we would also like to ask your partner (or family member or other close relative) to complete a questionnaire about your psychiatric well-being three times.

6. Possible side effects

We cannot predict in advance whether you will suffer from the dexamethasone. We do not expect any additional side effects from the study medication, because the study medication is almost equal to the amount of the body's own cortisol that would normally be produced. We refill a shortage. In our opinion, participating in the study will therefore not entail any additional risks in addition to the already existing risks of your treatment.

7. Possible advantages and disadvantages

It is important that you carefully consider the possible advantages and disadvantages before you decide to participate.

<u>Benefits</u> of participating:

- The study medication could reduce the possible psychiatric side effects of dexamethasone. Especially feelings of fear and restlessness could occur less, but this is not certain.
- The study medication could improve your sleep quality, but this is not certain.

<u>Disadvantages</u> of participating:

- It is important that you take the medicines regularly and according to the instructions.
- The test moments where questionnaires have to be completed take time and can be tiring.
- You have appointment that you must fulfill.
- The questionnaires can be confronting, because they ask about how you feel and how you are doing in daily life.
- Memory, attention and language tests will reveal your strengths and weaknesses. This can sometimes be confronting.

8. If you do not wish to participate or wish to stop participating

It is up to you to decide whether or not to participate in the study. Participation is voluntary. If you do not want to participate, you will continue your usual treatment as before. You do not have to sign anything and you do not have to explain why you do not want to participate.

If you do participate in the study, you can always change your mind. You may stop participation at any time during the study. This is not harmful and will not affect your usual treatment. You do not have to say why you are stopping, but you do need to tell the investigator immediately. The data collected until that time can still be used for the study.

Koning A-SCAM, *et al. BMJ Open* 2021; 11:e054405. doi: 10.1136/bmjopen-2021-054405

If you wish, and if applicable, collected body material can be destroyed. You must indicate this yourself. If you do not report this, it will be used for the investigation.

If there is new information about the study that is important to you, the researcher will let you know. You will then be asked if you want to continue participating.

9. End of the study

Your participation in the study will end if:

- all test moments are done and the questionnaires have been submitted
- you choose to stop yourself
- the doctor thinks it's better for you to stop
- the government or the assessing medical-ethical review committee, decides to stop the research.

The entire study ends when all participants completed the study.
The medication you took during the study will not be available after the study.

After processing all the data, the researcher will inform you about the most important results of the study. This happens approximately 2 to 3 years after your participation.
The researcher can also tell you what treatment you received. If you do not want this, you can say so to the researcher. The researcher is not allowed to tell you.

10. Use and storage of your data and body material

For this research, your personal data and, if applicable, body material will be collected, used and stored. This concerns data such as your name, address, date of birth and data about your health. The collection, use and storage of your data and possibly your body material is necessary to answer the questions posed in this study and to publish the results. We ask your permission for the use of your data and possible body material.

Confidentiality of your data and possible body material

To protect your privacy, we give a code to your data and, if applicable, your body material. Your name and other information that can directly identify you are omitted. Data can only be traced back to you with the key of the code. The key to the code remains stored in the local research facility. Also in reports and publications about the research, the data cannot be traced back to you.

Access your data

Some individuals may have access to all of your data at the study site. Also to the data without code. This is necessary to be able to check whether the research has been carried out properly and reliably. Persons who can access your data for inspection are: the committee that monitors

the safety of the research, a monitor who works for the LUMC and the Health and Youth Care Inspectorate. They keep your details secret. We ask you to give permission for this inspection.

Storing period of the data and body material

Your data must be kept at the study location for 15 years. If applicable, your body material will be stored indefinitely at the local research facility. We intend further research with these materials after this study. Research into the body material is carried out in the LUMC. If you have not been treated in the center in question, your body material will be brought to the LUMC for the examination, storage and examination. The data and body material sent to the LUMC from a participating hospital only contain the code, and not your name or other data with which you can be identified.

Withdraw permission

You can always withdraw your consent for the use of your personal data and possible body material. This applies to this research and also to the storage and use for future research. The research data collected up to the moment you withdraw your consent will still be used in the research. Your body material will only be destroyed at your request. If measurements have already been taken with the body material, those data will still be used.

More information about your rights when processing data

For general information about your rights when processing your personal data, you can consult the website of the Dutch Data Protection Authority.

If you have any questions about your rights, please contact the person responsible for processing your personal data. For this study, that is: the LUMC. See **Appendix A**: Contact Details.

If you have any questions or complaints about the processing of your personal data, we recommend that you first contact the research location. You can contact the Data Protection Officer of the institution or the Dutch Data Protection Authority. See **Appendix A**: Contact Details.

Registration of the study

Information about this study is also included in an overview of medical scientific studies, namely in the Dutch Trial Register (http://www.trialregister.nl). It does not contain any data that can be traced back to you. After finishing the study, the website may display a summary of the results of this survey. You will find this study under the name: DEXA-CORT.

11. Insurance for study participants

Insurance has been taken out for everyone participating in this study. The insurance covers damage caused by the study. Not all damage is covered. In **Appendix B**: Information about the insurance, you will find more information about the insurance and the exceptions. It also states who you can report the damage to.

12. Informing the general practitioner and treating specialist

We will not inform your GP that you are participating in the study. If you prefer, you can always contact your GP yourself to let them know that you are participating in the study. If we have any questions about your medical history or medication use, we will contact your GP or treating physician. You cannot participate in the study if you do not have a GP. Your treating physician will be informed about your participation.

13. No compensation for participating

The study medication for the study are free of charge for you. You will not be paid for participating in this study. Study appointments are scheduled on days when you already have a hospital appointment. If we cannot schedule coincide appointments and you have to visit the hospital only for study appointments, you will be reimbursed for your travel costs.

14. Any questions?

If you have any questions, please contact the researcher or the research team. For independent advice about participating in this study, you can contact the independent doctor; Dr. Stijn W. Genders. He knows a lot about the research, but he is not involved in this study. If you have any complaints about the study, you can discuss this with the researcher or your attending physician. If you prefer not to do this, you can contact the complaints officer of your hospital. All details can be found in **Appendix A**: Contact details.

15. Signing the consent

You do not have to decide right away whether you want to participate in the study. When you have had sufficient reflection time of at least 1 week and a maximum of 2 weeks, you will be asked to decide whether to participate in this study. If you give permission, we will ask you to confirm this in writing on the accompanying statement of consent. By your written consent, you indicate that you have understood the information and agree to participate in the study. Both you and the researcher will receive a signed version of this consent form.

Thank you for your attention.

16. Appendices

A. Contact details

B. Insurance information

C. Schedule of investigation actions

D. Additional background information

E. Informed Consent Form

F. Informed Consent Form (for family or other relative)

G. Brochure 'Medical research. General information for research participants'

Appendix A: Contact details

**Coordinating investigator**	**Independent expert**
Anne-Sophie Koning, MSc	Dr. S.W. Genders
dexonderzoek@lumc.nl	oog.stafsecretariaat@lumc.nl
+31-71-5265303 / +31-71-5263082	+31-71-5262374
**LUMC contact details**	
Dr. W.R. van Furth	
neurochirurgie@lumc.nl	
+31-71-5262109	
**Data protection officers of the LUMC**	
If you have any questions about the protection of your privacy, you can contact the data protection officers of the LUMC (FG) via infoavg@lumc.nl	
**Complaints officer of the LUMC**	
In case of complaints, you can report to the patient service desk in the LUMC, location H2-11 (route number 473, opposite Leidseplein). Here you can report your dissatisfaction and fill in the complaint form. The patient service bureau will inform you as soon as possible about a possible solution and may call in the complaints officer if necessary. You can also fill in the complaint form digitally. See website LUMC page submit a complaint.<br><br>**Contact details patient service desk**<br>LUMC Patient service desk<br>Postbus 9600<br>2300 RC Leiden<br>Phone: +31 71 5262989	

Appendix B: Insurance information

The LUMC has taken out insurance for everyone who participates in this study. The insurance covers damage caused by participating in the study. This applies to damage during the study or within four years of the end of your participation in the study. You must have reported damage to the insurer within those four years.

The insurance does not cover all damage. At the bottom of this text is a brief description of which damage is not covered.
These provisions are contained in the Decree on compulsory insurance for medical research involving humans. This decision can be found on www.ccmo.nl, the website of the Central Committee for Research on Human Subjects (see 'Library' and then 'Laws and regulations').

In the event of damage, you can contact the insurer directly.

The insurance company is:	
Name:	Onderlinge Waarborgmaatschappij Centramed B.A.
Address:	Maria Montessorilaan 9, 2719 DB Zoetermeer
Phone:	070-3017070
Email:	info@centramed.nl
Policy number:	624.530.305

The insurance offers cover of € 650,000 per test participant and € 5,000,000 for the entire study (and € 7,500,000 per year for all studies of the Leiden University Medical Center).

The insurance does **not** cover the following damage::

- damage from a risk of which you have been informed in the written information. This does not apply if the risk is more serious than anticipated or if the risk was very unlikely;
- damage to your health that would also have occurred if you had not taken part in the study;
- damage caused by not (fully) following directions or instructions;
- damage to your descendants, as a result of a negative effect of the research on you or your descendants;
- damage caused by an existing treatment method when researching existing treatment methods.

Appendix C: Schedule of investigation actions

No additional hospital visits are required for this study. The examinations to be performed are scheduled on the days you are in the hospital.

Outpatient clinic visit

According to regular care, an outpatient clinic appointment is scheduled with the neurosurgeon. On the day of this outpatient clinic appointment, we would like to ask you to fill in 1 questionnaire, to have 1 interview and to perform a number of tests for memory, attention and language. If possible, also 1 questionnaire for your partner (or family member or other relative). We want to do an extra language screening for patients with a left-sided brain tumor. In total, this study moment will last approximately 80 (or 70) minutes.
We will also send you 5 questionnaires via email, which you can complete at home. It will take approximately 45 minutes to complete all questionnaires.

Days on the ward

You do not need to do anything on the day of admission and on the day of surgery. After the surgery you will stay in the hospital for a few days according to regular care. The number of days you will be in the hospital depends on the judgment of the doctors. This is determined according to regular care. On the days that you stay on the nursing ward, we always ask you to fill in 1 short questionnaire. We also ask you to wear a watch with which we can measure your activity and sleep quality. In addition, we would like to ask you to briefly indicate in a sleep diary how you slept. The time you spend in the hospital on the examination these days is a maximum of 10 minutes. During your hospital stay, nurses will monitor your psychiatric well-being.

Day of discharge

On the day you are allowed to go home, we would like to conduct an interview with you, we will ask you to complete 2 questionnaires and we will perform a short screening for memory, attention and language. We would also like to ask your partner (or family member or other close relative) to complete 1 questionnaire. A language screening is conducted for patients with a left-sided brain tumor (mostly according to regular care). This study moment will last approximately 55 (or 45) minutes.

After the surgery

About 2 weeks after the surgery, we would like to ask you to fill in 4 questionnaires. You can do this at home and completing all questionnaires will take approximately 30 minutes in total. According to regular care, an appointment in the hospital is scheduled around 5 to 8 weeks after surgery. On this day in the hospital, we would like to conduct the interview one more time, we ask you to fill in 2 more questionnaires and to perform a number of tests for memory, attention and language. If possible, also 1 questionnaire for your partner (or family member or other close relative). This study moment will last approximately 80 minutes.

About 3 months after surgery, we would like to ask you to fill in 5 questionnaires. You can do this at home and completing all the questionnaires will take approximately 45 minutes in total.

In total, the study will take about 5 to 6 hours of your time spread over 3 months.

**Study moment**	**Where**	**How long**
Preoperative	Outpatient clinic	70 (tot 80) minutes
	Home	45 minutes
During admission	Ward	30 minutes
Day of discharge	Ward	45 (tot 55) minutes
2 weeks postoperative	Home	30 minutes
5 – 8 weeks postoperative	Outpatient clinic	80 minutes
3 months postoperative	Home	45 minutes
**Total**		**5 – 6 hr**

Version 4, 14-02-20

Appendix D – Additional background information

Our body produces the hormone cortisol. Cortisol affects many things, such as our mood and our sleep. Cortisol is produced in the adrenal glands, which are regulated by another hormone called adrenocorticotropic hormone (ACTH). The ACTH is made in the pituitary gland, an organ under the brain. When there is enough cortisol, the pituitary gland receives a signal to stop making hormones, this way the adrenal glands no longer make cortisol.

The drug dexamethasone is very similar to cortisol that the body can make itself. But it is much stronger than the body's own cortisol. A small dose of dexamethasone can already ensure that the pituitary gland no longer makes a hormone. This causes the adrenal glands to stop making cortisol. So a side effect of treatment with dexamethasone is that our own bodies no longer make cortisol.

There is one big difference between cortisol and dexamethasone. Dexamethasone works through a single target. This is through the glucocorticoid receptor (GR). Cortisol acts on two targets: via the GR, but also via the mineralocorticoid receptor (MR). Cortisol thus activates MR and GR. Dexamethasone only activates the GR. Because our body no longer makes cortisol after dexamethasone, cortisol can no longer activate the MR. The result is that only the GR is active, while the MR remains empty. We think that the side effects after dexamethasone are because the MR is empty, because the functioning of cortisol in the brain depends on the MR.

In this study we add cortisol to the treatment with dexamethasone (as a pill, cortisol has been given the name 'hydrocortisone'). The hydrocortisone can bind to the MR. In this way we hope to get the normal functioning of the MR again. And we hope that the side effects will decrease.

During the study, you may be asked whether blood may be taken from you and stored for further examination. We want to check in the blood which type of MR you have. We can investigate this by looking at the gene of the MR in the DNA. It is known that a certain variant of the MR gene gives a lower risk of getting depression. In this study we want to see whether this can be an underlying reason for the complaints that some patients get from the dexamethasone.

We want to investigate the effect of the hydrocortisone within the sample tissue that is removed from the removed tumor tissue. We expect the MR to become active again with the added hydrocortisone. We want to measure that in this tissue.

Koning A-SCAM, *et al. BMJ Open* 2021; 11:e054405. doi: 10.1136/bmjopen-2021-054405

Version 4, 14-02-20

Appendix E: Informed Consent form participant

Cortisol to prevent psychiatric adverse effects of dexamethasone

- I have read the study information letter. I was also able to ask questions. My questions have been answered to my satisfaction. I had enough time to decide whether to participate.
- I know that participation is voluntary. I know that I may decide at any time not to participate after all or to withdraw from the study. I do not need to give a reason for this.
- I give permission to request information from my general practitioner if there are any questions about your medical history or medication use.
- I know that for the purpose of auditing and monitoring the study, some people may have access to all my data. These people are mentioned in this information letter. I give permission for that access by these persons.
- I know that my partner (or family member or other relative) also has to fill in a questionnaire several times and he/she agrees to this.
- I give permission for the collection and use of my data to answer the research question in this study.
- I □ **do**
  □ **do not**
  consent for blood collection and to keep and use this for future research in the field of research described in the information letter.
- I □ **do**
  □ **do not**
  consent to keep a sample of the removed tumor tissue and to keep it and use it for future research in the field of research described in the information letter.
- I □ **do**
  □ **do not**
  consent to keep my personal data and body material longer and use it for future research in the field of research described in the information letter.

- I □ **do**
  □ **do not**
  want to be informed on the treatment I received (placebo or hydrocortisone)

- I want to participate in this study.

Name study participant: ___________________

Signature:________________________ Date : __ / __ / __

I hereby declare that I have fully informed this study participant about this study.

If information comes to light during the course of the study that could affect the study participant's consent, I will inform him/her of this in a timely fashion.

Name investigator (or its representative):_______________________________

Signature:_______________________ Date: __ / __ / __

Version 4, 14-02-20

Appendix F: Informed Consent form partner/other relative

Cortisol to prevent psychiatric adverse effects of dexamethasone

- I am aware that my partner (or family member or other close relative) is participating in the study. I was also able to ask questions. My questions have been answered to my satisfaction. I have had enough time to decide whether I want to fill in a questionnaire about my partner (or relative or other close relative) 3 times for the study.

- I know that participation is voluntary. I know that I may decide at any time not to participate after all or to withdraw from the study. I do not need to give a reason for this.

- I want to participate in this study.

Name partner (or family member or other relative):______________

Signature:_______________________ Date : __ / __ / __

I hereby declare that I have fully informed this partner (or other close relative) about this study.

If information comes to light during the course of the study that could affect the partner's (or other close relative's) consent, I will inform him/her of this in a timely fashion.

Name investigator (or its representative):______________________________

Signature:______________________ Date: __ / __ / __

Koning A-SCAM, *et al. BMJ Open* 2021; 11:e054405. doi: 10.1136/bmjopen-2021-054405
