# Supplementary material for: The DEXA-CORT trial: study protocol of a randomised placebo-controlled trial of hydrocortisone in patients with brain tumour on the prevention of neuropsychiatric adverse effects caused by perioperative dexamethasone
Source: BMJ Open. 2021 Dec 28;11(12):e054405. doi: 10.1136/bmjopen-2021-054405 (PMC8719188; doi:10.1136/bmjopen-2021-054405)
Supplement: Author's manuscript [file bmjopen-2021-054405.draft_revisions.pdf]

BMJ Open is committed to open peer review. As part of this commitment we make the peer review history of every article we publish publicly available.

When an article is published we post the peer reviewers' comments and the authors' responses online. We also post the versions of the paper that were used during peer review. These are the versions that the peer review comments apply to.

The versions of the paper that follow are the versions that were submitted during the peer review process. They are not the versions of record or the final published versions. They should not be cited or distributed as the published version of this manuscript.

BMJ Open is an open access journal and the full, final, typeset and author-corrected version of record of the manuscript is available on our site with no access controls, subscription charges or pay-per-view fees (http://bmjopen.bmj.com).

If you have any questions on BMJ Open's open peer review process please email info.bmjopen@bmj.com

BMJ Open

The DEXA-CORT trial: study protocol of a randomized placebo-controlled trial of hydrocortisone in brain tumour patients on the prevention of neuropsychiatric adverse effects caused by perioperative dexamethasone

Journal:	*BMJ Open*
Manuscript ID	bmjopen-2021-054405
Article Type:	Protocol
Date Submitted by the Author:	16-Jun-2021

Complete List of Authors:	Koning, Anne-Sophie; Leiden University Medical Center, Department of Medicine, Division of Endocrinology<br>Satoer, Djaina; Erasmus Medical Center, Department of Neurosurgery<br>Vinkers, C; Amsterdam UMC Locatie VUmc, Department of Psychiatry and Department of Anatomy and Neurosciences; GGZ inGeest, Academic Working Place Depression<br>Zamanipoor Najafabadi, Amir H.; University Neurosurgical Center Holland, Leiden University Medical Center, Haaglanden Medical Center and Haga Teaching Hospitals<br>Biermasz, Nienke; Leiden University Medical Center, Department of Medicine, Division of Endocrinology, and Centre for Endocrine Tumors Leiden (CETL)<br>Nandoe Tewarie, Rishi D.S.; University Neurosurgical Center Holland, Leiden University Medical Center, Haaglanden Medical Center and Haga Teaching Hospitals<br>Moojen, Wouter A.; University Neurosurgical Center Holland, Leiden University Medical Center, Haaglanden Medical Center and Haga Teaching Hospitals<br>van Rossum, Elisabeth; Erasmus Medical Center, Department of Internal Medicine, Division of Endocrinology<br>Dirven, Clemens; Erasmus Medical Center, Department of Neurosurgery<br>Pereira, Alberto; Leiden University Medical Center, Department of Medicine, Division of Endocrinology<br>van Furth, W.R.; University Neurosurgical Center Holland, Leiden University Medical Center, Haaglanden Medical Center and Haga Teaching Hospitals, Department of neurosurgery<br>Meijer, Onno C.; Leiden University Medical Center, Department of Medicine, Division of Endocrinology

Keywords:	NEUROSURGERY, PSYCHIATRY, Clinical trials < THERAPEUTICS, CLINICAL PHARMACOLOGY, General endocrinology < DIABETES & ENDOCRINOLOGY

I, the Submitting Author has the right to grant and does grant on behalf of all authors of the Work (as defined in the below author licence), an exclusive licence and/or a non-exclusive licence for contributions from authors who are: i) UK Crown employees; ii) where BMJ has agreed a CC-BY licence shall apply, and/or iii) in accordance with the terms applicable for US Federal Government officers or employees acting as part of their official duties; on a worldwide, perpetual, irrevocable, royalty-free basis to BMJ Publishing Group Ltd ("BMJ") its licensees and where the relevant Journal is co-owned by BMJ to the co-owners of the Journal, to publish the Work in this journal and any other BMJ products and to exploit all rights, as set out in our licence.

The Submitting Author accepts and understands that any supply made under these terms is made by BMJ to the Submitting Author unless you are acting as an employee on behalf of your employer or a postgraduate student of an affiliated institution which is paying any applicable article publishing charge ("APC") for Open Access articles. Where the Submitting Author wishes to make the Work available on an Open Access basis (and intends to pay the relevant APC), the terms of reuse of such Open Access shall be governed by a Creative Commons licence – details of these licences and which Creative Commons licence will apply to this Work are set out in our licence referred to above.

Other than as permitted in any relevant BMJ Author's Self Archiving Policies, I confirm this Work has not been accepted for publication elsewhere, is not being considered for publication elsewhere and does not duplicate material already published. I confirm all authors consent to publication of this Work and authorise the granting of this licence.

For peer review only

The DEXA-CORT trial: study protocol of a randomized placebo-controlled trial of hydrocortisone in brain tumour patients on the prevention of neuropsychiatric adverse effects caused by perioperative dexamethasone

Anne-Sophie C.A.M. Koning[1], Djaina D. Satoer[2], Christiaan H. Vinkers[3,4], Amir H. Zamanipoor Najafabadi[5], Nienke R. Biermasz[1], Rishi D.S. Nandoe Tewarie[5], Wouter A. Moojen[5], Elisabeth F.C. van Rossum[6], Clemens M.F. Dirven[2], Alberto M. Pereira[1]*, Wouter R. van Furth[5]* and Onno C. Meijer[1]

*equal contribution

Affiliations:

[1] Department of Medicine, Division of Endocrinology, Leiden University Medical Center, Leiden, The Netherlands

[2] Department of Neurosurgery, Erasmus MC - University Medical Center, Rotterdam, The Netherlands

[3] Department of Psychiatry and Department of Anatomy and Neurosciences, Amsterdam University Medical Center, location VUMC, Amsterdam, The Netherlands

[4] Academic Working Place Depression, GGZ InGeest, Amsterdam, The Netherlands

[5] University Neurosurgical Center Holland, Leiden University Medical Center, Haaglanden Medical Center and Haga Teaching Hospitals, Leiden and The Hague, The Netherlands

[6] Department of Internal Medicine, Division of Endocrinology, Erasmus MC, University Medical Center Rotterdam, Rotterdam, The Netherlands

Contributors: OCM was main applicant in the funding acquisition. ACAM, DDS, AHZN, NRB, EFCR, CHV, CMFD, AMP, WRF and OCM participated in the design of the study. CMFD, DDS, AMP, WRF and RDSN are principal investigators at the participating study centres. CMFD, DDS, RDSN, WAM, ACAM initiated and implemented the study design at the participating study centre. ACAM is the coordinating investigator with DDS as daily supervisor. ACAM drafted the manuscript. All authors reviewed and approved the final manuscript.

Funding: This work is supported by The Netherlands Organisation for Health Research and Development (ZonMw) and Dutch Brain Foundation grant project number 95105005.

Competing interests: Authors have nothing to disclose.

Corresponding author: Anne-Sophie C.A.M. Koning, a.c.a.koning@lumc.nl

Word count: 4000

For peer review only - http://bmjopen.bmj.com/site/about/guidelines.xhtml

ABSTRACT

Introduction: The synthetic glucocorticoid dexamethasone can induce serious neuropsychiatric adverse effects. Dexamethasone activates the glucocorticoid receptor (GR) but, unlike endogenous cortisol, not the mineralocorticoid receptor (MR). Moreover, dexamethasone suppresses cortisol production, thereby eliminating its MR binding. Consequently, GR overactivation combined with MR underactivation may contribute to the neuropsychiatric adverse effects of dexamethasone. The DEXA-CORT trial aims to re-activate the MR using cortisol to reduce neuropsychiatric adverse effects of dexamethasone treatment.

Methods and analysis: The DEXA-CORT study is a multi-centre, randomised, double-blind, placebo-controlled trial in adult patients who undergo elective brain tumour resection treated perioperatively with high doses of dexamethasone to minimize cerebral oedema. Hundred-eighty patients are randomised between treatment with either twice daily 10mg hydrocortisone or placebo during dexamethasone treatment. The primary study outcome is the difference in proportion of patients scoring ≥3 points on at least one of the Brief Psychiatric Rating Scale (BPRS) questions five days postoperatively, or earlier at discharge. Secondary outcomes are neuropsychiatric symptoms, quality of sleep, health-related quality of life (HRQoL) and neurocognitive functioning at several time points postoperatively. Furthermore, neuropsychiatric history, (serious) adverse events (S(AEs)), prescribed (psychiatric) medication and referrals or evaluations of psychiatrist/psychologist, and laboratory measurements are assessed.

Ethics and dissemination: The study protocol has been approved by the Medical Research Ethics Committee of the Leiden University Medical Center and by the Dutch competent authority. It is an investigator-initiated study with financial support by The Netherlands Organisation for Health Research and Development (ZonMw) and the Dutch Brain Foundation.

Trial registration number: NL6726 (Netherlands Trial Register); open for patient inclusion. EudraCT number 2017-003705-17.

Strengths and limitations of this study

- The DEXA-CORT study is the first double-blind, placebo-controlled intervention trial in brain tumour patients aiming to reduce neuropsychiatric adverse effects of perioperative dexamethasone using medication, which is biochemically identical to our body’s naturally produced hormone cortisol.

For peer review only - http://bmjopen.bmj.com/site/about/guidelines.xhtml

- The DEXA-CORT trial has an elegant hypothesis-driven approach to restore the physiological balance of MR and GR activation.
- Dexamethasone is used in many medical fields, and our trial has great potential for many patients worldwide that experience adverse effects of dexamethasone.
- Not all patients experience adverse effects following dexamethasone treatment, and patients without adverse effects are included.

For peer review only

INTRODUCTION

Synthetic glucocorticoids (GCs) are widely used across many different clinical disciplines, in particular immunology, pulmonology, neurosurgery, and oncology. Their potent anti-inflammatory and immunosuppressive effects and ability to control cerebral oedema ensures effective treatment (1-3). However, moderate to high doses of synthetic GCs frequently cause severe side effects (4). These include serious neuropsychiatric conditions such as sleep disturbances, delirium, depression, mania, psychosis and suicidality (5). Despite these mentioned problems, dexamethasone is a standard treatment in brain tumour patients for more than 50 years since no better alternatives are present (6-8). Evidently, these adverse effects negatively affect well-being and quality of life. In particular, depressive symptoms, a common problem in brain tumour patients, appeared to be an independent predictor for quality of life rating (9). The risks of glucocorticoid induced neuropsychiatric adverse effects in this and other patient groups is therefore serious (10). At present, there is no preventive treatment for these unwanted and serious neuropsychiatric adverse effects.

The naturally produced hormone cortisol acts on both the glucocorticoid receptor (GR) and mineralocorticoid receptor (MR), whereas the synthetic GC dexamethasone mainly activates GR with low or absent binding to the MR *in vivo* (11). The increased GR activity is mediating the therapeutic goal of the treatment, i.e., reduction of oedema. However, dexamethasone strongly suppresses endogenous cortisol production through GR-mediated negative feedback mechanisms in the hypothalamus-pituitary-adrenal (HPA) axis. (12, 13). The MR is also a prominent cortisol receptor in the brain, and its activation by cortisol binding strongly decreases after the HPA axis suppression (14). While low concentrations of dexamethasone poorly penetrate the blood-brain-barrier (15), higher clinically used doses do reach the brain. This has led to the hypothesis that, in addition to the strongly stimulated GR, reduced brain MR activity may underlie the neuropsychiatric adverse effects during dexamethasone treatment (16).

MR activity has indeed been related to psychopathology. Correlatively, decreased MR expression has been observed in several psychiatric disorders (17). Functionally, both pharmacological intervention studies and a genetic gain-of-function variant of MR seem to have protective effects (18-20). Therefore, we hypothesize that increasing MR activity with physiological doses of cortisol (= hydrocortisone) may prevent or substantially diminish the disrupting neuropsychiatric effects in brain tumour patients that receive high doses of perioperative dexamethasone, see Figure 1. Such a positive effect of adding hydrocortisone was previously found in a subgroup of children who experienced severe adverse effects after dexamethasone treatment for childhood leukaemia (21). Therefore, the aim of the present study is to investigate the effects of hydrocortisone replacement in adult brain

For peer review only - http://bmjopen.bmj.com/site/about/guidelines.xhtml

tumour patients, compared to placebo, as add-on to the treatment with dexamethasone, on the development of neuropsychiatric adverse effects that can be caused by dexamethasone.

For peer review only

For peer review only - http://bmjopen.bmj.com/site/about/guidelines.xhtml

METHODS ANDS ANALYSIS

Study design and setting

The DEXA-CORT study is a multicentre, randomized, double-blind, placebo-controlled trial investigating the effect of hydrocortisone replacement as co-treatment to reduce perioperative dexamethasone related neuropsychiatric adverse effects in patients with brain tumours undergoing elective neurosurgery. A total of hundred-eighty patients will be randomised in a 1:1 ratio, stratified per study centre and on tumour type. The participating centres are Leiden University Medical Center (LUMC), Erasmus University Medical Center (Erasmus MC), Haaglanden Medical Center (HMC), and University Medical Center Utrecht (UMCU). The primary outcome measure is the proportion of patients with neuropsychiatric symptoms 5 days after surgery – or earlier if discharge is within 5 days – compared between the hydrocortisone and placebo treated group. The study is investigator-initiated with the LUMC as sponsor, and is funded by ZonMw and the Dutch Brain Foundation.

Eligibility criteria

Adult patients (≥18y) diagnosed with a brain tumour (glioma or meningioma) and undergoing elective surgical resection are eligible for inclusion. All patients receive high dose dexamethasone according to institutional protocols. Since peri-operative dexamethasone dosing schemes differ among the participating centres a minimal peri-operative cumulative dexamethasone dose of 24mg or more in 6 days is required. Initially, preoperative dexamethasone of more than one day was considered an exclusion criterium. During the course of the study it appeared that many patients, especially those with malignant tumours and severe oedema, had dexamethasone prescribed several days before the planned surgery. Consequently many patients were not eligible for study participation, threatening completion of inclusion. Hence this exclusion criterium was discarded with consent of the Data Safety Monitoring Board (DSMB). Detailed inclusion and exclusion criteria are presented in Figure 2.

Study outline

A timeline of the study is shown in Figure 3. Eligible patients are approached in an outpatient setting by specialized nurses in consultation with the treating neurosurgeon. The study is explained and written information is provided. After written informed consent, patient characteristics (age, gender, educational level, medical background, medication use, tumour type and tumour location) are recorded. Before start of intervention, a baseline study visit is planned that includes a structured psychiatric interview, different self-report questionnaires, and neurocognitive tests. A selection of the questionnaires can be filled out online via the web-based database program Castor Electronic Data

For peer review only - http://bmjopen.bmj.com/site/about/guidelines.xhtml

Capture (EDC; Castor, Amsterdam, The Netherlands). The intervention starts on the day of hospital admission, concurrent with the dexamethasone treatment one day prior to planned surgery. Five days postoperatively – or earlier at discharge – a structured psychiatric interview and neurocognitive screening are conducted, and questionnaires are filled out. The study medication is provided to the patients according to clinical care and is continued as long as dexamethasone is prescribed. A follow-up visit is planned five to eight weeks postoperatively coinciding with the regular care assessment, at which a structured psychiatric interview and neurocognitive tests are performed and different questionnaires are filled out. Study visits take place at the medical centre of treatment. Two weeks and three months postoperatively questionnaires can be filled out online via Castor EDC. Study duration from start of intervention until completion of the last questionnaires is three months. The study schedule is shown in Table 1. In case the baseline study visit is planned the day before surgery, no neurocognitive tests will be performed – instead a neurocognitive screening may be performed – and presentation of the questionnaires is depend on the patients' psychological and physical condition.

Intervention

Patients will be treated according to routine clinical care, based on local treatment protocols. The investigational product is hydrocortisone with placebo as comparator, both produced by Tiofarma B.V., Oud-Beijerland, The Netherlands. The Clinical Pharmacy and Toxicology department of the LUMC will repack, label and randomize the study medication. Hundred-eighty patients are randomised between treatment with either twice daily 10mg hydrocortisone or placebo, both orally administered concurrent with the full course of perioperative dexamethasone treatment; mostly with a dexamethasone taper schedule. Study medication is available for twenty days; assuming the slow dexamethasone taper schedule is used, treatment will continue on average for fifteen days. In the event that dexamethasone treatment re-starts after completion of its taper schedule, study medication will not be available. The study medication is prescribed by the physician and administration details are recorded. Treatment dosage is targeted towards physiological cortisol replacement.

Allocation, randomisation and blinding

Randomisation is controlled by the Clinical Pharmacy and Toxicology department of LUMC using block randomisation in a 1:1 ratio stratified for each medical centre participating in the study, and for tumour type (glioma or meningioma) with block size of ten. The randomisation list is stored at the department and will not be disclosed until all participants have completed the trial. Only in case of

For peer review only - http://bmjopen.bmj.com/site/about/guidelines.xhtml

emergency and the knowledge of the assigned treatment is required the randomisation code can be broken. The study is blinded for everyone involved, except the pharmacy who allocates participants to treatment. In addition, hydrocortisone and placebo pills are identical in terms of appearance and taste ensuring blinding.

Assessments during the study

Assessments during the study include neuropsychiatric symptoms, quality of sleep, health-related quality of life (HRQoL), neurocognitive assessment, registration of (serious) adverse events ((S)AEs), prescribed medication, and referrals to psychiatrist/psychologist. Furthermore, blood and residual tissue from tumour specimens will be collected to verify MR targeting and molecular effects of hydrocortisone co-treatment.

Neuropsychiatric symptoms

Brief Psychiatric Rating Scale (BPRS)

Neuropsychiatric symptoms will be measured with the BPRS version 4.0 (22). The BPRS is an interview, which provides a description of major symptom characteristics in psychiatric patients. The BPRS covers the presence and severity of 24 symptoms scored on a 7 point scale (1 = not present, 7 = extremely severe). Possible scores vary from 24 to 168. The BPRS will be used for our primary outcome.

Mini-screen

A mini-screen will be used to determine the patient's neuropsychiatric history. This mini-screen comprises 22 'Yes' or 'No' questions about different neuropsychiatric symptoms (23).

Hospital Anxiety and Depression Scale (HADS)

To determine the levels of anxiety and depression in patients, the HADS questionnaire will be used (24). The HADS comprises 14 items; seven related to anxiety and seven to depression. Each question has four different answer options, ranging from 0 to 3. Patients scoring 8-10 on the anxiety or depression domain are classified as possibly suffering from anxiety or depression, respectively, and a score ≥11 as definite anxiety or depression.

Positive and Negative Affect Schedule (PANAS)

To determine the positive and negative affect of patients the PANAS is used. The PANAS comprises a 20-item mood scale yielding two subscales; positive affect (PA) and negative affect (NA) (25). Patients rate how they felt 'in general' on a 5-point scale; 1 = very slightly or not at all, 2 = a little, 3 =

For peer review only

For peer review only - http://bmjopen.bmj.com/site/about/guidelines.xhtml

moderately, 4 = quite a bit and 5 = very much. PA scores range from 10-50, with higher scores representing higher levels of positive affect. NA scores range form 10-50, with lower scores representing lower levels of negative affect.

<u>The Altman Self-Rating Mania Scale (ASRM)</u>

The ASRM is used to assess the presence and/or severity of manic symptoms (26). The ASRM consists of 5-items consisting of 5 statements. At the 5 items, the patient chooses one statement that best describes the way he or she has been feeling for the past week.

<u>Neuropsychiatric Inventory Question (NPI-Q)</u>

The NPI-Q is a questionnaire to be filled out by a family member or care-taker of the patient, that specifically assesses neuropsychiatric symptoms of the patient in the last month (27-29). The NPI-Q consist of 12 domains of symptoms. Every domain needs to be answered with 'yes' (is present) or 'no' (is absent). When the symptom is present, the partner rates the severity of the symptom (3-point scale) and the psychiatric burden (6-point scale) for him/herself. The NPI-Q gives a total score for the severity (range 0 – 36) and a total score for emotional burden (range 0 – 60).

<u>Delirium Observation Screening (DOS)</u>

The DOS scale is used to determine the presence of delirium in patients and includes 13 observation items of behaviour that show symptoms of delirium (30). Nurses measure the different items during the day, evening and night. Each item has three answer options; 'never', 'sometimes – always' and 'not known'. These three scores are then scored with 0, 1 or no points. The total DOS scale score is the sum of the total DOS score at day, evening and night divided by 3. Patients scoring ≥3 are probably suffering from delirium.

Quality of sleep

To determine the sleep quality the Leeds Sleep Evaluation Questionnaire (LSEQ) is used (31, 32). LSEQ contact information and permission to use is available from Mapi Research Trust, Lyon, France, https://eprovide.mapi-trust.org. LSEQ is a reliable instrument to assess sleep quality changes during psycho-pharmacological treatment intervention. This questionnaire is a self-report measure with 10-items consisting of a 10-cm-line visual analogue rating scale that evaluates four domains; ease of initiating sleep, quality of sleep, ease of waking and behaviour following wakefulness. The LSEQs 10-cm-horizontal line has two extreme states defined at the end of the line (e.g. Tired = score of 0, Alert

For peer review only

For peer review only - http://bmjopen.bmj.com/site/about/guidelines.xhtml

= score of 10). Patients respond by placing a vertical mark on the line. Each question is scored out of 10 (e.g. 0 cm = score 0,5 cm = score of 5).

HRQoL

HRQoL will be measured with the EORTC core quality of life questionnaire (QLQ-C30) complemented with the QLQ-BN20 (33, 34). The QLQ-C30 is specific in assessing the HRQoL of cancer patients participating in clinical trials. It consists of 30 items, of which 28 have a four-point scale (1 = not at all, 4 = very much). Two questions have a seven-point scale (1 = very poor, 7 = excellent). A high score represents a higher response level; high/healthy level of functioning, high QoL and a high level of symptoms. The QLQ-BN20 is a validated module specific for patients with brain neoplasms. It includes 20 items with a four-point scale (1 = not at all, 4 = very much) and the higher the score the more symptoms or problems.

Neurocognitive assessment

To assess the cognitive domains verbal memory, executive functioning and attention, we used the following standardized neurocognitive tests: HVLT-R – Free Recall (35), Trail Making Test A and B (36), Controlled Oral Word Association (37) and MOS Scale (38). Raw scores can be transformed into z-scores in order to compare patients' performance to control participants (and can be corrected for demographic variables, such as age and education).

The Montreal Cognitive Assessment (MoCA) is a cognitive screening instrument for mild cognitive dysfunction (39). It measures different cognitive domains; attention and concentration, executive functions, memory, language, visuo-constructional skills, conceptual thinking, calculations and orientation. The total score is 30 points; a score of ≥26 is a normal score.

The Aphasia Bedside Check (ABC) is a 10-min screening tool that globally determines the presence of aphasia with seven items for language comprehension and seven items for language production (40). This screening will only be performed in patients who have a left sided brain tumour with (hetero-)anamnestic language problems. With ≥3 mistakes an aphasia may be present and the patient should not be included in the study.

(Serious) Adverse Events ((S)AEs)– Medication – Referral to psychiatrist/psychologist

(S)AEs, prescribed medication (especially psychiatric medication) and referrals or evaluations of psychiatrist/psychologist will be recorded.

For peer review only - http://bmjopen.bmj.com/site/about/guidelines.xhtml

Laboratory measures

We will collect one tube of blood taken during anaesthetic procedures for surgery. This will be used for DNA analyses to determine MR haplotype. Additionally, cortisol and glucose levels will be measured in blood.

If possible during the surgical procedure tumour tissue and peritumoral infiltrated brain tissue will be collected to investigate MR and GR and the effect of cortisol on these receptors.

Primary outcome

The primary study outcome is the difference in proportion of patients scoring ≥3 points on at least one of the BPRS questions five days postoperatively – or earlier at discharge – between the hydrocortisone and placebo group. A better outcome on the BPRS in the hydrocortisone group would be considered a positive result, which would indicate that addition of hydrocortisone to dexamethasone is beneficial to reduce neuropsychiatric adverse effects of dexamethasone.

Secondary outcomes

Secondary outcomes are neuropsychiatric symptoms, quality of sleep, HRQoL, and neurocognitive functioning five days postoperatively – or earlier at discharge – , two weeks, five to eight weeks, and three months postoperatively. Furthermore, neuropsychiatric history, SAEs, prescribed (psychiatric) medication and referrals or evaluations of psychiatrist/psychologist will be collected. In addition, the laboratory measurements are considered secondary outcomes.

Sample size

The sample size calculation is based on the results of a pilot study in which we tested the BPRS in our study population. We determined postoperative BPRS scores to evaluate the presence of psychiatric symptoms in brain tumour patients who underwent surgery and were treated with dexamethasone. We interviewed 18 patients on the day of hospital discharge. According to the BPRS scores in this group we considered a score of ≥3 on at least one of the BPRS items as clinically relevant for patients. We found that 7 out of 18 patients (≈40%) scored at least ≥3 on one of the BPRS items. Based on expert clinical judgement a 50% reduction in the patients with a score ≥3 is perceived as relevant. This equals an absolute difference of 20%. If we consider an $\alpha = 0.05$ and a power of 80%, and we take the 20% absolute difference between the two treatment arms, a sample size of 82 patients in each group would be needed. Based on an expected drop-out rate of ≈10%, 90 patients in each treatment arm (in total 180 patients) will be considered as a satisfactory sample size.

For peer review only - http://bmjopen.bmj.com/site/about/guidelines.xhtml

Planned statistical analyses

This study is a randomized controlled trial. All data will be analysed on an intention-to-treat basis.

BPRS

The primary study parameter is the proportion of patients scoring ≥3 points on at least one of the BPRS questions five days postoperatively – or earlier at discharge. The difference in these proportions between the placebo group and the hydrocortisone group will be calculated and statistically compared with the Chi-Square test. Any difference with a $p<0.05$ will be considered significant.

As secondary analyses, both treatment arms will be compared over time using analysis of repeated measures, generalized estimating equation (GEE) for the dichotomous outcomes as described in the primary outcome and the mixed linear model for the BPRS as continuous outcome. Additionally, we will look at the differences between the treatment arms on the separate items of the BRPS.

Mini-screen, HADS, PANAS, ASRM, NPI-Q, DOS, LSEQ and HRQoL

Both treatment arms will be compared over time using analysis of repeated measures, GEE when outcome is dichotomous and mixed linear models for continuous outcomes.

Explanatory univariable and multivariable regression analysis will be used to identify (baseline) predictors of treatment response. Sensitivity analysis will be performed using multiple imputation to handle missing data. Any difference with a $p<0.05$ will be considered significant. A better outcome on the different scales in the hydrocortisone group would be considered a positive result.

Neurocognitive functioning

The neurocognitive tests are a combination of neuropsychological tests for which normative data were published, so that patient's performance can be corrected according to age, gender and education. We will compare the raw data between the treatment arms with a t-test. Test scores can be transformed into z-values using normative data and compared with a one sample t-test. With no-normal distributions the appropriate statistical tests will be used.

Subgroup analyses

Subgroup analyses will be performed to explore differences in our primary and secondary parameters for type of tumour, gender, age and dosage of dexamethasone.

As the study remains blinded until completion, no interim analyses are planned. In addition, the statistical plan will be finalized before starting the analyses.

For peer review only - http://bmjopen.bmj.com/site/about/guidelines.xhtml

Data management

All data will be collected, stored, and analysed in a coded manner, and in accordance with the General Data Protection Regulation (GDPR). Data are collected and stored in the web-based database management system Castor EDC using electronic case report forms (CRFs). Source documents include medical records, paper CRFs, paper interviews, questionnaires and neuropsychological tests. All data is pseudonymised with a study code and will be stored for 15 years.

Safety

Participating in the trial is very unlikely to bring additional risks for patients, since hydrocortisone is given in a low dose to restore the physiological cortisol level in the body.

DSMB

This study established a DSMB that protects and guarantees patients safety during the trial. The DSMB includes three members with different study backgrounds: a statistician, psychiatrist and an internist/clinical epidemiologist. The role and responsibilities of the DSMB is described in the DSMB charter document.

AEs and SAEs

The study population has a high risk of complications, which are inherent to their vulnerable condition (neurological deficits caused by a brain tumour, surgery, high dose dexamethasone) and these are considered unrelated to the intervention. The patient population is expected to encounter many AEs not related to the experimental design, therefore not all AE will be registered. AEs that will be registered are: psychiatric complaints of which psychiatric consultation and medication is needed, brain oedema for which increased dexamethasone medication is given, or for which the dexamethasone reduction scheme is extended, all infections for which antibiotics are needed, cerebrospinal fluid (CSF) leakage that requires surgical intervention, hyperglycaemia for which medication is needed, cardiovascular events requiring intervention, epileptic seizures requiring intervention and secondary adrenal insufficiency that requires hydrocortisone suppletion. The abovementioned AEs will be registered from the start of the intervention until completion of the trial.

SAEs that we will record are any untoward medical occurrence or effect that results in death, is life threatening (at the time of the event), requires hospitalisation or prolongation of existing inpatients' hospitalisation – including intensive care – when a patient has unexpected longer IC admission or unexpected >10 days hospitalisation, results in persistent or significant disability or incapacity – like neurological dysfunction – lasting for more than 3 days postoperatively, results in any other important

For peer review only - http://bmjopen.bmj.com/site/about/guidelines.xhtml

medical event not mentioned above due to medical or surgical intervention and judged as such by the investigators and post-operative haemorrhage for which re-craniotomy is needed. An elective hospital admission will not be considered a SAE. Whether related to the study medication or not, all these SAEs occurring during the intervention period will be reported through the web portal governed and issued by the Dutch National Ethics Committee (CCMO) called 'ToetsingOnline'. SAEs occurring after the intervention period will be line-listed and not reported in ToetsingsOnline.

Monitoring

According to Good Research Practice (GRP) independent on-site monitoring visits will be performed to ensure the conduct of the trial is consistent with the approved protocol(s), the rights and well-being of the subjects are protected, source documents are verified and data is accurately reported from the source documents. Monitoring visits will take place according to the study specific monitoring plan.

Patients and public involvement

Patients and public were not involved in the study design. The ZonMw committee that granted the funding includes a representative of the patients interests. The committee is kept informed annually.

For peer review only

For peer review only - http://bmjopen.bmj.com/site/about/guidelines.xhtml

ETHICS AND DISSEMINATION

The study protocol has been approved by the accredited Medical Research Ethics Committee of the LUMC and by the Dutch competent authority. Permission for execution of the study is obtained at each participating site. Amendments of the protocol will first be sent for approval and upon consent the participating centres will be informed. The study will be conducted according to the principles of the Declaration of Helsinki, Good Clinical Practice guidelines – including centre specific requirements – and in accordance with the Dutch Medical Research Involving Human Subjects Act (WMO). This trial is registered in the Dutch Trial Register (NTR). Trial results will be published and presented at conferences.

Trial status

The first patient was included on 10 September 2019 and recruitment is ongoing. Inclusion rate has slowed down due to the COVID-19 pandemic from March 2020 onwards. End of recruitment is expected to be 2022. The current study protocol version is V3, August 28. 2020.

For peer review only

For peer review only - http://bmjopen.bmj.com/site/about/guidelines.xhtml

DISCUSSION

The DEXA-CORT trial is the first double-blind, placebo-controlled trial in adult brain tumour patients, in which we investigate the possible prevention of neuropsychiatric adverse effects of perioperative dexamethasone treatment. Dexamethasone is a well-known very potent activator of the GR and consequently suppresses endogenous adrenocortical cortisol production via negative feedback on the HPA-axis, depleting brain MR from its ligand (12-14). The resultant disturbed balance in receptor activation in the brain may predispose for psychopathology. In this study, we test the hypothesis that re-instating brain MR activity via co-treatment with a physiological dose of cortisol (=hydrocortisone) will improve (specific features of) neuropsychological well-being. Because of the high dexamethasone doses and high affinity of dexamethasone for GR, cortisol treatment will only affect MR occupancy and not interfere with the therapeutic goal of dexamethasone treatment, which is GR activation.

This cortisol re-fill approach was already performed in childhood leukaemia patients. Patients who experienced severe adverse effects of dexamethasone seemed to benefit from the additional hydrocortisone medication (21). To establish this potential positive effect in an adult population, the DEXA-CORT trial is investigating this principle in adult brain tumour patients. Dexamethasone is a standard clinical treatment in brain tumour patients perioperatively and adverse effects are also present in this patient group, which leads to reduced HRQoL. If additional hydrocortisone to dexamethasone proves effective in reducing its neuropsychiatric adverse effects, it would offer a new treatment option for this patient group and maybe even for many other patients that receive long-term treatment with dexamethasone, and other synthetic GCs.

For peer review only - http://bmjopen.bmj.com/site/about/guidelines.xhtml

REFERENCES

1. Ismail MF, Lavelle C, Cassidy EM. Steroid-induced mental disorders in cancer patients: a systematic review. Future Oncol. 2017;13(29):2719-31.
2. Hockey B, Leslie K, Williams D. Dexamethasone for intracranial neurosurgery and anaesthesia. J Clin Neurosci. 2009;16(11):1389-93.
3. Arvold ND, Armstrong TS, Warren KE, Chang SM, DeAngelis LM, Blakeley J, et al. Corticosteroid use endpoints in neuro-oncology: Response Assessment in Neuro-Oncology Working Group. Neuro Oncol. 2018;20(7):897-906.
4. Strehl C, Bijlsma JW, de Wit M, Boers M, Caeyers N, Cutolo M, et al. Defining conditions where long-term glucocorticoid treatment has an acceptably low level of harm to facilitate implementation of existing recommendations: viewpoints from an EULAR task force. Ann Rheum Dis. 2016;75(6):952-7.
5. Judd LL, Schettler PJ, Brown ES, Wolkowitz OM, Sternberg EM, Bender BG, et al. Adverse consequences of glucocorticoid medication: psychological, cognitive, and behavioral effects. Am J Psychiatry. 2014;171(10):1045-51.
6. Wen PY, Schiff D, Kesari S, Drappatz J, Gigas DC, Doherty L. Medical management of patients with brain tumors. J Neurooncol. 2006;80(3):313-32.
7. Galicich JH, French LA, Melby JC. Use of dexamethasone in treatment of cerebral edema associated with brain tumors. J Lancet. 1961;81:46-53.
8. Dietrich J, Rao K, Pastorino S, Kesari S. Corticosteroids in brain cancer patients: benefits and pitfalls. Expert Rev Clin Pharmacol. 2011;4(2):233-42.
9. Pelletier G, Verhoef MJ, Khatri N, Hagen N. Quality of life in brain tumor patients: the relative contributions of depression, fatigue, emotional distress, and existential issues. J Neurooncol. 2002;57(1):41-9.
10. Fardet L, Petersen I, Nazareth I. Suicidal behavior and severe neuropsychiatric disorders following glucocorticoid therapy in primary care. Am J Psychiatry. 2012;169(5):491-7.
11. Grossmann C, Scholz T, Rochel M, Bumke-Vogt C, Oelkers W, Pfeiffer AF, et al. Transactivation via the human glucocorticoid and mineralocorticoid receptor by therapeutically used steroids in CV-1 cells: a comparison of their glucocorticoid and mineralocorticoid properties. Eur J Endocrinol. 2004;151(3):397-406.
12. Spencer RL, Deak T. A users guide to HPA axis research. Physiol Behav. 2017;178:43-65.
13. De Kloet R, Wallach G, McEwen BS. Differences in corticosterone and dexamethasone binding to rat brain and pituitary. Endocrinology. 1975;96(3):598-609.
14. Karssen AM, Meijer OC, Berry A, Sanjuan Pinol R, de Kloet ER. Low doses of dexamethasone can produce a hypocorticosteroid state in the brain. Endocrinology. 2005;146(12):5587-95.
15. Meijer OC, de Lange EC, Breimer DD, de Boer AG, Workel JO, de Kloet ER. Penetration of dexamethasone into brain glucocorticoid targets is enhanced in mdr1A P-glycoprotein knockout mice. Endocrinology. 1998;139(4):1789-93.
16. Meijer OC, de Kloet ER. A Refill for the Brain Mineralocorticoid Receptor: The Benefit of Cortisol Add-On to Dexamethasone Therapy. Endocrinology. 2017;158(3):448-54.
17. ter Heegde F, De Rijk RH, Vinkers CH. The brain mineralocorticoid receptor and stress resilience. Psychoneuroendocrinology. 2015;52:92-110.
18. Klok MD, Giltay EJ, Van der Does AJ, Geleijnse JM, Antypa N, Penninx BW, et al. A common and functional mineralocorticoid receptor haplotype enhances optimism and protects against depression in females. Transl Psychiatry. 2011;1:e62.
19. Otte C, Hinkelmann K, Moritz S, Yassouridis A, Jahn H, Wiedemann K, et al. Modulation of the mineralocorticoid receptor as add-on treatment in depression: a randomized, double-blind, placebo-controlled proof-of-concept study. J Psychiatr Res. 2010;44(6):339-46.

For peer review only

For peer review only - http://bmjopen.bmj.com/site/about/guidelines.xhtml

20. Otte C, Wingenfeld K, Kuehl LK, Kaczmarczyk M, Richter S, Quante A, et al. Mineralocorticoid receptor stimulation improves cognitive function and decreases cortisol secretion in depressed patients and healthy individuals. Neuropsychopharmacology. 2015;40(2):386-93.
21. Warris LT, van den Heuvel-Eibrink MM, Aarsen FK, Pluijm SM, Bierings MB, van den Bos C, et al. Hydrocortisone as an Intervention for Dexamethasone-Induced Adverse Effects in Pediatric Patients With Acute Lymphoblastic Leukemia: Results of a Double-Blind, Randomized Controlled Trial. J Clin Oncol. 2016;34(19):2287-93.
22. Ventura J, Lukoff D, Nuechterlein KH. Brief Psychiatric Rating Scale (BPRS) Expanded Version (4.0): Scales, Anchor Points and Administration Manual. . International Journal of Methods in Psychiatric Research. 1993;3:227-44.
23. Sheehan DV, Lecrubier Y, Sheehan KH, Amorim P, Janavs J, Weiller E, et al. The Mini-International Neuropsychiatric Interview (M.I.N.I.): the development and validation of a structured diagnostic psychiatric interview for DSM-IV and ICD-10. J Clin Psychiatry. 1998;59 Suppl 20:22-33;quiz 4-57.
24. Zigmond AS, Snaith RP. The hospital anxiety and depression scale. Acta Psychiatr Scand. 1983;67(6):361-70.
25. Watson D, Clark LA, Tellegen A. Development and validation of brief measures of positive and negative affect: the PANAS scales. J Pers Soc Psychol. 1988;54(6):1063-70.
26. Altman EG, Hedeker D, Peterson JL, Davis JM. The Altman Self-Rating Mania Scale. Biol Psychiatry. 1997;42(10):948-55.
27. Cummings JL, Mega M, Gray K, Rosenberg-Thompson S, Carusi DA, Gornbein J. The Neuropsychiatric Inventory: comprehensive assessment of psychopathology in dementia. Neurology. 1994;44(12):2308-14.
28. Kaufer DI, Cummings JL, Ketchel P, Smith V, MacMillan A, Shelley T, et al. Validation of the NPI-Q, a brief clinical form of the Neuropsychiatric Inventory. J Neuropsychiatry Clin Neurosci. 2000;12(2):233-9.
29. de Jonghe JF, Kat MG, Kalisvaart CJ, Boelaarts L. [Neuropsychiatric inventory questionnaire (NPI-Q): A validity study of the Dutch form]. Tijdschr Gerontol Geriatr. 2003;34(2):74-7.
30. Schuurmans MJ, Shortridge-Baggett LM, Duursma SA. The Delirium Observation Screening Scale: a screening instrument for delirium. Res Theory Nurs Pract. 2003;17(1):31-50.
31. Parrott AC, Hindmarch I. Factor analysis of a sleep evaluation questionnaire. Psychol Med. 1978;8(2):325-9.
32. Hindmarch I. A 1,4-benzodiazepine, temazepam (K 3917), its effect on some psychological parameters of sleep and behaviour. Arzneimittelforschung. 1975;25(11):1836-9.
33. Aaronson NK, Ahmedzai S, Bergman B, Bullinger M, Cull A, Duez NJ, et al. The European Organization for Research and Treatment of Cancer QLQ-C30: a quality-of-life instrument for use in international clinical trials in oncology. J Natl Cancer Inst. 1993;85(5):365-76.
34. Taphoorn MJ, Claassens L, Aaronson NK, Coens C, Mauer M, Osoba D, et al. An international validation study of the EORTC brain cancer module (EORTC QLQ-BN20) for assessing health-related quality of life and symptoms in brain cancer patients. Eur J Cancer. 2010;46(6):1033-40.
35. Brandt J, Benedict RHB. HVLT-R: Hopkins Verbal Learning Test-Revised. Copyright 1991, 1998, 2001 by PAR, Inc
36. Tombaugh TN. Trail Making Test A and B: normative data stratified by age and education. Arch Clin Neuropsychol. 2004;19(2):203-14.
37. Benton AL, Hamsher KdS. Multilingual Aphasia Examination. Copyright 1978 by PAR, Inc.
38. Stewart AL, Greenfield S, Hays RD, Wells K, Rogers WH, Berry SD, et al. Functional Status and Well-Being of Patients with Chronic Conditions - Results from the Medical Outcomes Study. Jama-J Am Med Assoc. 1989;262(7):907-13.
39. Nasreddine ZS, Phillips NA, Bedirian V, Charbonneau S, Whitehead V, Collin I, et al. The Montreal Cognitive Assessment, MoCA: a brief screening tool for mild cognitive impairment. J Am Geriatr Soc. 2005;53(4):695-9.

For peer review only

For peer review only - http://bmjopen.bmj.com/site/about/guidelines.xhtml

40. Visch-Brink E, El Hachioui H. *Afasie Bedside Check*. Erasmus MC, Rotterdam, Nederland. 2013.

For peer review only

For peer review only - http://bmjopen.bmj.com/site/about/guidelines.xhtml

TABLES

Table 1. The DEXA-CORT study schedule of enrolment, intervention and assessments.

	**STUDY PERIOD**								
	**Outpatient clinic**	**Baseline**	**Treatment**				**Follow-up**	**Follow-up**	**Close-out**
			**admission**	**surgery**	**ward**	**discharge**			
**Days**		-14 – -1	-1	0	1 – 4	3 – 5	14	35 – 56	84
**Visits**	V_0	V_1	V_1/V_2					V_2/V_3	
**Enrolment:**									
Eligibility screen	X								
Informed consent		X	X						
Baseline characteristics		X	X						
Allocation		X	X						
**Interventions:**									
Hydrocortisone			X →	→	→	→	→		
Placebo			X →	→	→	→	→		
**Assessments:**									
BPRS		X	X			X		X	
Mini-screen		X	X						
HADS		X	(X)				X		X
PANAS		X	(X)		X	X	X	X	X
ASRM		X	(X)				X		X
NPI-Q		X	X			X		X	
DOS					X				
LSEQ		X	(X)			X	X	X	X
QoL-C30/BN20		X	(X)						X
Cognitive tests		X						X	
MoCA			(X)			X			
ABC		X	X			X			
SAEs			X →	→	→	→	→		

For peer review only

AEs		X							X
Medication		X							X
Psychiatric referrals		X							X
**Laboratory measures:**									
Tube of blood				X					
Residual tumour tissue				X					

BPRS: Brief Psychiatric Rating Scale, HADS: Hospital Anxiety and Depression Scale, PANAS: Positive And Negative Affect Schedule, ASRM: Altman Self-Rating Mania Scale, NPI-Q: Neuropsychiatric Inventory Question, DOS: Delirium Observation Screening, LSEQ: Leeds Sleep Evaluation Questionnaire, QoL: Quality of Life questionnaires, MoCA: Montreal Cognitive Assessment, ABC: Aphasia Bedside Check, (S)AEs: (Serious) Adverse Events.

For peer review only - http://bmjopen.bmj.com/site/about/guidelines.xhtml

FIGURE LEGENDS

Figure 1: Preventing neuropsychiatric adverse effects of dexamethasone with co-administration of cortisol. Dexamethasone suppresses the HPA-axis and thereby the adrenocortical cortisol production, leaving cortisol-preferring MRs unoccupied. Re-activating the MR using cortisol might reduce or prevent neuropsychiatric adverse effects of dexamethasone treatment via activation of brain MR.

Figure 2: Eligibility criteria of the DEXA-CORT study.

Figure 3: The DEXA-CORT study timeline.

For peer review only

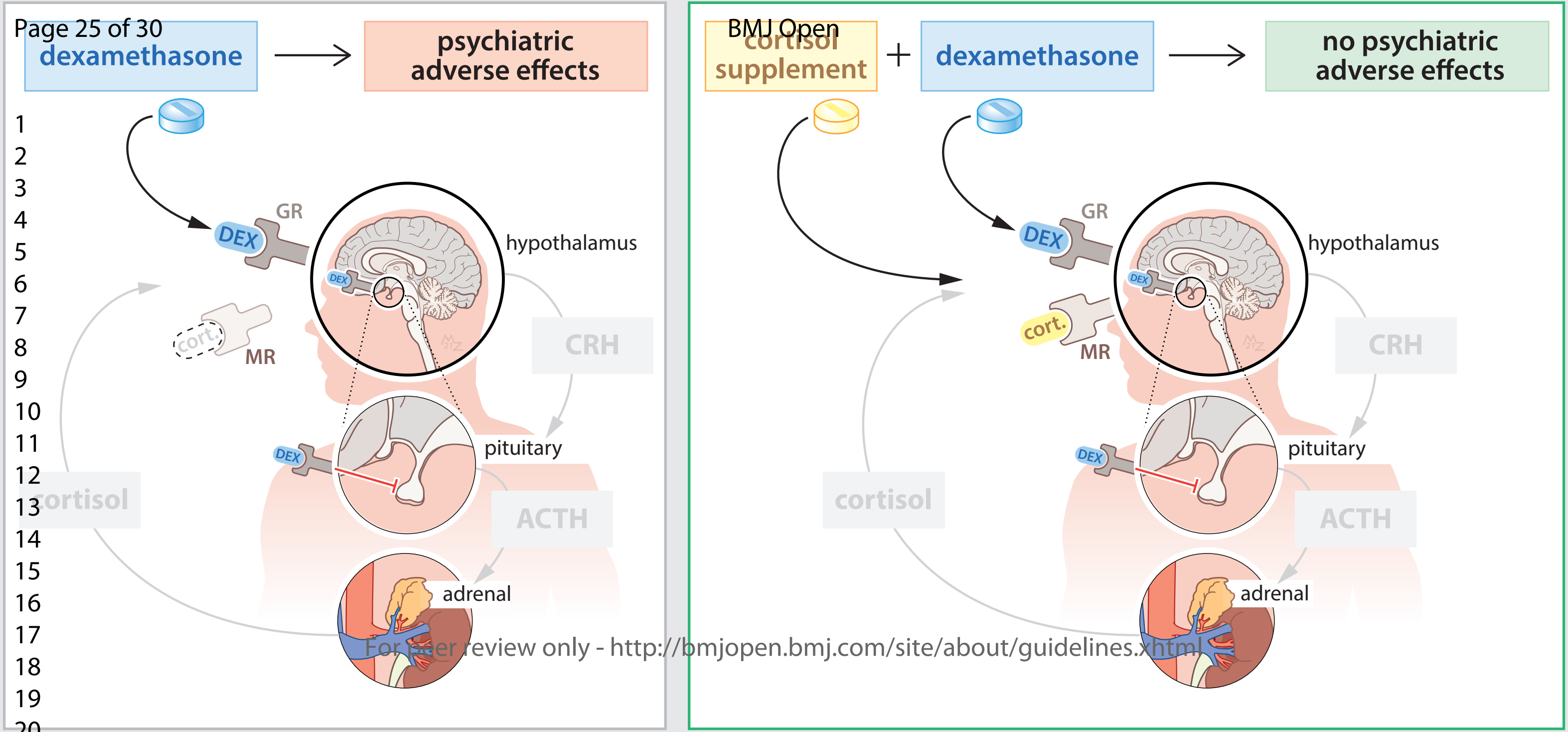

For peer review only - http://bmjopen.bmj.com/site/about/guidelines.xhtml

Inclusion criteria:

- Age = ≥18 years
- Cranial glioma or meningioma patients scheduled to undergo surgery (resection)
- Minimal dose of peri-operative cumulative dexamethasone exposure of 24mg or more in 6 days
- Good clinical condition; KPS≥70
- Life expectancy ≥6 months

Exclusion criteria:

- Non-native speakers of Dutch or insufficient command of the Dutch language
- Patients that are unable to overview consequences of trial participation
- Patients with severe aphasia
- Patients that are not able to fill in the questionnaires because of cognitive impairments at the discretion of the physician
- Patients with psychiatric diseases or neurological deficits that interfere with the study to the judgement of treating physician

For peer review only - http://bmjopen.bmj.com/site/about/guidelines.xhtml

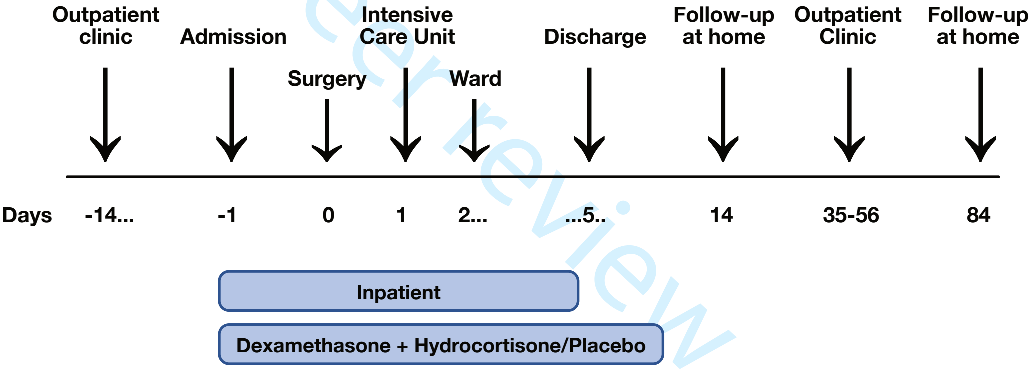

For peer review only

SPIRIT

STANDARD PROTOCOL ITEMS: RECOMMENDATIONS FOR INTERVENTIONAL TRIALS

SPIRIT 2013 Checklist: Recommended items to address in a clinical trial protocol and related documents*

Section/item	Item No	Description
**Administrative information**		
Title	1	Descriptive title identifying the study design, population, interventions, and, if applicable, trial acronym
Trial registration	2a	Trial identifier and registry name. If not yet registered, name of intended registry
	2b	All items from the World Health Organization Trial Registration Data Set
Protocol version	3	Date and version identifier
Funding	4	Sources and types of financial, material, and other support
Roles and responsibilities	5a	Names, affiliations, and roles of protocol contributors
	5b	Name and contact information for the trial sponsor
	5c	Role of study sponsor and funders, if any, in study design; collection, management, analysis, and interpretation of data; writing of the report; and the decision to submit the report for publication, including whether they will have ultimate authority over any of these activities
	5d	Composition, roles, and responsibilities of the coordinating centre, steering committee, endpoint adjudication committee, data management team, and other individuals or groups overseeing the trial, if applicable (see Item 21a for data monitoring committee)
**Introduction**		
Background and rationale	6a	Description of research question and justification for undertaking the trial, including summary of relevant studies (published and unpublished) examining benefits and harms for each intervention
	6b	Explanation for choice of comparators
Objectives	7	Specific objectives or hypotheses
Trial design	8	Description of trial design including type of trial (eg, parallel group, crossover, factorial, single group), allocation ratio, and framework (eg, superiority, equivalence, noninferiority, exploratory)

For peer review only

Methods: Participants, interventions, and outcomes

Study setting	9	Description of study settings (eg, community clinic, academic hospital) and list of countries where data will be collected. Reference to where list of study sites can be obtained
Eligibility criteria	10	Inclusion and exclusion criteria for participants. If applicable, eligibility criteria for study centres and individuals who will perform the interventions (eg, surgeons, psychotherapists)
Interventions	11a	Interventions for each group with sufficient detail to allow replication, including how and when they will be administered
	11b	Criteria for discontinuing or modifying allocated interventions for a given trial participant (eg, drug dose change in response to harms, participant request, or improving/worsening disease)
	11c	Strategies to improve adherence to intervention protocols, and any procedures for monitoring adherence (eg, drug tablet return, laboratory tests)
	11d	Relevant concomitant care and interventions that are permitted or prohibited during the trial
Outcomes	12	Primary, secondary, and other outcomes, including the specific measurement variable (eg, systolic blood pressure), analysis metric (eg, change from baseline, final value, time to event), method of aggregation (eg, median, proportion), and time point for each outcome. Explanation of the clinical relevance of chosen efficacy and harm outcomes is strongly recommended
Participant timeline	13	Time schedule of enrolment, interventions (including any run-ins and washouts), assessments, and visits for participants. A schematic diagram is highly recommended (see Figure)
Sample size	14	Estimated number of participants needed to achieve study objectives and how it was determined, including clinical and statistical assumptions supporting any sample size calculations
Recruitment	15	Strategies for achieving adequate participant enrolment to reach target sample size

Methods: Assignment of interventions (for controlled trials)

Allocation:

Sequence generation	16a	Method of generating the allocation sequence (eg, computer-generated random numbers), and list of any factors for stratification. To reduce predictability of a random sequence, details of any planned restriction (eg, blocking) should be provided in a separate document that is unavailable to those who enrol participants or assign interventions

For peer review only - http://bmjopen.bmj.com/site/about/guidelines.xhtml

Allocation concealment mechanism	16b	Mechanism of implementing the allocation sequence (eg, central telephone; sequentially numbered, opaque, sealed envelopes), describing any steps to conceal the sequence until interventions are assigned
Implementation	16c	Who will generate the allocation sequence, who will enrol participants, and who will assign participants to interventions
Blinding (masking)	17a	Who will be blinded after assignment to interventions (eg, trial participants, care providers, outcome assessors, data analysts), and how
	17b	If blinded, circumstances under which unblinding is permissible, and procedure for revealing a participant's allocated intervention during the trial

✓ **Methods: Data collection, management, and analysis**

Data collection methods	18a	Plans for assessment and collection of outcome, baseline, and other trial data, including any related processes to promote data quality (eg, duplicate measurements, training of assessors) and a description of study instruments (eg, questionnaires, laboratory tests) along with their reliability and validity, if known. Reference to where data collection forms can be found, if not in the protocol
	18b	Plans to promote participant retention and complete follow-up, including list of any outcome data to be collected for participants who discontinue or deviate from intervention protocols
Data management	19	Plans for data entry, coding, security, and storage, including any related processes to promote data quality (eg, double data entry; range checks for data values). Reference to where details of data management procedures can be found, if not in the protocol
Statistical methods	20a	Statistical methods for analysing primary and secondary outcomes. Reference to where other details of the statistical analysis plan can be found, if not in the protocol
	20b	Methods for any additional analyses (eg, subgroup and adjusted analyses)
	20c	Definition of analysis population relating to protocol non-adherence (eg, as randomised analysis), and any statistical methods to handle missing data (eg, multiple imputation)

✓ **Methods: Monitoring**

Data monitoring	21a	Composition of data monitoring committee (DMC); summary of its role and reporting structure; statement of whether it is independent from the sponsor and competing interests; and reference to where further details about its charter can be found, if not in the protocol. Alternatively, an explanation of why a DMC is not needed

	21b	Description of any interim analyses and stopping guidelines, including who will have access to these interim results and make the final decision to terminate the trial
Harms	22	Plans for collecting, assessing, reporting, and managing solicited and spontaneously reported adverse events and other unintended effects of trial interventions or trial conduct
Auditing	23	Frequency and procedures for auditing trial conduct, if any, and whether the process will be independent from investigators and the sponsor
✓ **Ethics and dissemination**		
Research ethics approval	24	Plans for seeking research ethics committee/institutional review board (REC/IRB) approval
Protocol amendments	25	Plans for communicating important protocol modifications (eg, changes to eligibility criteria, outcomes, analyses) to relevant parties (eg, investigators, REC/IRBs, trial participants, trial registries, journals, regulators)
Consent or assent	26a	Who will obtain informed consent or assent from potential trial participants or authorised surrogates, and how (see Item 32)
	26b	Additional consent provisions for collection and use of participant data and biological specimens in ancillary studies, if applicable
Confidentiality	27	How personal information about potential and enrolled participants will be collected, shared, and maintained in order to protect confidentiality before, during, and after the trial
Declaration of interests	28	Financial and other competing interests for principal investigators for the overall trial and each study site
Access to data	29	Statement of who will have access to the final trial dataset, and disclosure of contractual agreements that limit such access for investigators
Ancillary and post-trial care	30	Provisions, if any, for ancillary and post-trial care, and for compensation to those who suffer harm from trial participation
Dissemination policy	31a	Plans for investigators and sponsor to communicate trial results to participants, healthcare professionals, the public, and other relevant groups (eg, via publication, reporting in results databases, or other data sharing arrangements), including any publication restrictions
	31b	Authorship eligibility guidelines and any intended use of professional writers
	31c	Plans, if any, for granting public access to the full protocol, participant-level dataset, and statistical code

For peer review only - http://bmjopen.bmj.com/site/about/guidelines.xhtml

Appendices

Informed consent materials	32	Model consent form and other related documentation given to participants and authorised surrogates
Biological specimens	33	Plans for collection, laboratory evaluation, and storage of biological specimens for genetic or molecular analysis in the current trial and for future use in ancillary studies, if applicable

*It is strongly recommended that this checklist be read in conjunction with the SPIRIT 2013 Explanation & Elaboration for important clarification on the items. Amendments to the protocol should be tracked and dated. The SPIRIT checklist is copyrighted by the SPIRIT Group under the Creative Commons "Attribution-NonCommercial-NoDerivs 3.0 Unported" license.

For peer review only

BMJ Open

The DEXA-CORT trial: study protocol of a randomized placebo-controlled trial of hydrocortisone in brain tumour patients on the prevention of neuropsychiatric adverse effects caused by perioperative dexamethasone

Journal:	*BMJ Open*
Manuscript ID	bmjopen-2021-054405.R1
Article Type:	Protocol
Date Submitted by the Author:	16-Oct-2021

Complete List of Authors:	Koning, Anne-Sophie; Leiden University Medical Center, Department of Medicine, Division of Endocrinology<br>Satoer, Djaina; Erasmus Medical Center, Department of Neurosurgery<br>Vinkers, C; Amsterdam UMC Locatie VUmc, Department of Psychiatry and Department of Anatomy and Neurosciences; GGZ inGeest, Academic Working Place Depression<br>Zamanipoor Najafabadi, Amir H.; University Neurosurgical Center Holland, Leiden University Medical Center, Haaglanden Medical Center and Haga Teaching Hospitals<br>Biermasz, Nienke; Leiden University Medical Center, Department of Medicine, Division of Endocrinology, and Centre for Endocrine Tumors Leiden (CETL)<br>Nandoe Tewarie, Rishi D.S.; University Neurosurgical Center Holland, Leiden University Medical Center, Haaglanden Medical Center and Haga Teaching Hospitals<br>Moojen, Wouter A.; University Neurosurgical Center Holland, Leiden University Medical Center, Haaglanden Medical Center and Haga Teaching Hospitals<br>van Rossum, Elisabeth; Erasmus Medical Center, Department of Internal Medicine, Division of Endocrinology<br>Dirven, Clemens; Erasmus Medical Center, Department of Neurosurgery<br>Pereira, Alberto; Leiden University Medical Center, Department of Medicine, Division of Endocrinology<br>van Furth, W.R.; University Neurosurgical Center Holland, Leiden University Medical Center, Haaglanden Medical Center and Haga Teaching Hospitals, Department of neurosurgery<br>Meijer, Onno C.; Leiden University Medical Center, Department of Medicine, Division of Endocrinology

**Primary Subject Heading**:	Neurology
Secondary Subject Heading:	Diabetes and endocrinology
Keywords:	NEUROSURGERY, PSYCHIATRY, Clinical trials < THERAPEUTICS, CLINICAL PHARMACOLOGY, General endocrinology < DIABETES & ENDOCRINOLOGY

I, the Submitting Author has the right to grant and does grant on behalf of all authors of the Work (as defined in the below author licence), an exclusive licence and/or a non-exclusive licence for contributions from authors who are: i) UK Crown employees; ii) where BMJ has agreed a CC-BY licence shall apply, and/or iii) in accordance with the terms applicable for US Federal Government officers or employees acting as part of their official duties; on a worldwide, perpetual, irrevocable, royalty-free basis to BMJ Publishing Group Ltd ("BMJ") its licensees and where the relevant Journal is co-owned by BMJ to the co-owners of the Journal, to publish the Work in this journal and any other BMJ products and to exploit all rights, as set out in our licence.

The Submitting Author accepts and understands that any supply made under these terms is made by BMJ to the Submitting Author unless you are acting as an employee on behalf of your employer or a postgraduate student of an affiliated institution which is paying any applicable article publishing charge ("APC") for Open Access articles. Where the Submitting Author wishes to make the Work available on an Open Access basis (and intends to pay the relevant APC), the terms of reuse of such Open Access shall be governed by a Creative Commons licence – details of these licences and which Creative Commons licence will apply to this Work are set out in our licence referred to above.

Other than as permitted in any relevant BMJ Author's Self Archiving Policies, I confirm this Work has not been accepted for publication elsewhere, is not being considered for publication elsewhere and does not duplicate material already published. I confirm all authors consent to publication of this Work and authorise the granting of this licence.

For peer review only

The DEXA-CORT trial: study protocol of a randomized placebo-controlled trial of hydrocortisone in brain tumour patients on the prevention of neuropsychiatric adverse effects caused by perioperative dexamethasone

Anne-Sophie C.A.M. Koning[1], Djaina D. Satoer[2], Christiaan H. Vinkers[3,4], Amir H. Zamanipoor Najafabadi[5], Nienke R. Biermasz[1], Rishi D.S. Nandoe Tewarie[5], Wouter A. Moojen[5], Elisabeth F.C. van Rossum[6], Clemens M.F. Dirven[2], Alberto M. Pereira[1]*, Wouter R. van Furth[5]* and Onno C. Meijer[1]

*equal contribution

Affiliations:

[1] Department of Medicine, Division of Endocrinology, Leiden University Medical Center, Leiden, The Netherlands

[2] Department of Neurosurgery, Erasmus MC - University Medical Center, Rotterdam, The Netherlands

[3] Department of Psychiatry and Department of Anatomy and Neurosciences, Amsterdam University Medical Center, location VUMC, Amsterdam, The Netherlands

[4] Academic Working Place Depression, GGZ InGeest, Amsterdam, The Netherlands

[5] University Neurosurgical Center Holland, Leiden University Medical Center, Haaglanden Medical Center and Haga Teaching Hospitals, Leiden and The Hague, The Netherlands

[6] Department of Internal Medicine, Division of Endocrinology, Erasmus MC, University Medical Center Rotterdam, Rotterdam, The Netherlands

Corresponding author: Anne-Sophie C.A.M. Koning, a.c.a.koning@lumc.nl

Word count: Abstract: 273, Main text: 4747

For peer review only

ABSTRACT

Introduction: The synthetic glucocorticoid dexamethasone can induce serious neuropsychiatric adverse effects. Dexamethasone activates the glucocorticoid receptor (GR) but, unlike endogenous cortisol, not the mineralocorticoid receptor (MR). Moreover, dexamethasone suppresses cortisol production, thereby eliminating its MR binding. Consequently, GR overactivation combined with MR underactivation may contribute to the neuropsychiatric adverse effects of dexamethasone. The DEXA-CORT trial aims to re-activate the MR using cortisol to reduce neuropsychiatric adverse effects of dexamethasone treatment.

Methods and analysis: The DEXA-CORT study is a multi-centre, randomised, double-blind, placebo-controlled trial in adult patients who undergo elective brain tumour resection treated perioperatively with high doses of dexamethasone to minimize cerebral oedema. 180 patients are randomised between treatment with either twice daily 10mg hydrocortisone or placebo during dexamethasone treatment. The primary study outcome is the difference in proportion of patients scoring ≥3 points on at least one of the Brief Psychiatric Rating Scale (BPRS) questions 5 days postoperatively, or earlier at discharge. Secondary outcomes are neuropsychiatric symptoms, quality of sleep, health-related quality of life (HRQoL) and neurocognitive functioning at several time points postoperatively. Furthermore, neuropsychiatric history, (serious) adverse events (S(AEs)), prescribed (psychiatric) medication and referrals or evaluations of psychiatrist/psychologist, and laboratory measurements are assessed.

Ethics and dissemination: The study protocol has been approved by the Medical Research Ethics Committee of the Leiden University Medical Center and by the Dutch competent authority. It is an investigator-initiated study with financial support by The Netherlands Organisation for Health Research and Development (ZonMw) and the Dutch Brain Foundation. Results of the study will be submitted for publication in a peer-reviewed journal.

Trial registration number: NL6726 (Netherlands Trial Register); open for patient inclusion. EudraCT number 2017-003705-17.

Strengths and limitations of this study

- The DEXA-CORT study is the first double-blind, placebo-controlled intervention trial in brain tumour patients aiming to reduce neuropsychiatric adverse effects of perioperative dexamethasone.

For peer review only - http://bmjopen.bmj.com/site/about/guidelines.xhtml

- Use of validated outcome measurements.
- Evaluation of acute and potential long-term effects of the intervention.
- Duration of dexamethasone treatment differs between participants.
- Not all patients experience adverse effects following dexamethasone treatment, and patients without adverse effects are included.

For peer review only

For peer review only - http://bmjopen.bmj.com/site/about/guidelines.xhtml

INTRODUCTION

Synthetic glucocorticoids (GCs) are widely used across many different clinical disciplines, in particular immunology, pulmonology, neurosurgery, and oncology. Their potent anti-inflammatory and immunosuppressive effects and ability to control cerebral oedema ensures effective treatment (1-3). However, moderate to high doses of synthetic GCs frequently cause severe side effects (4). These include serious neuropsychiatric conditions such as sleep disturbances, delirium, depression, mania, psychosis and suicidality (5). Despite these mentioned problems, dexamethasone is a standard treatment in brain tumour patients for more than 50 years since no better alternatives are present (6-8). Evidently, these adverse effects negatively affect well-being and quality of life. In particular, depressive symptoms, a common problem in brain tumour patients, appeared to be an independent predictor for quality of life rating (9). The risks of glucocorticoid induced neuropsychiatric adverse effects in this and other patient groups is therefore serious (10). At present, there is no preventive treatment for these unwanted and serious neuropsychiatric adverse effects.

The naturally produced hormone cortisol acts on both the glucocorticoid receptor (GR) and mineralocorticoid receptor (MR), whereas the synthetic GC dexamethasone mainly activates GR with low or absent binding to the MR *in vivo* (11). The increased GR activity is mediating the therapeutic goal of the treatment, i.e., reduction of oedema. However, dexamethasone strongly suppresses endogenous cortisol production through GR-mediated negative feedback mechanisms in the hypothalamus-pituitary-adrenal (HPA) axis. (12, 13). The MR is also a prominent cortisol receptor in the brain, and its activation by cortisol binding strongly decreases after the HPA axis suppression (14). While low concentrations of dexamethasone poorly penetrate the blood-brain-barrier (15), higher clinically used doses do reach the brain. This has led to the hypothesis that, in addition to the strongly stimulated GR, reduced brain MR activity may underlie the neuropsychiatric adverse effects during dexamethasone treatment (16).

MR activity has indeed been related to psychopathology. Correlatively, decreased MR expression has been observed in several psychiatric disorders (17). Functionally, both pharmacological intervention studies and a genetic gain-of-function variant of MR seem to have protective effects (18-20). Therefore, we hypothesize that increasing MR activity with physiological doses of cortisol (= hydrocortisone) may prevent or substantially diminish the disrupting neuropsychiatric effects in brain tumour patients that receive high doses of perioperative dexamethasone, see Figure 1. Such a positive effect of adding hydrocortisone was previously found in a subgroup of children who experienced severe adverse effects after dexamethasone treatment for childhood leukaemia (21). Therefore, the aim of the present study is to investigate the effects of hydrocortisone replacement in adult brain

For peer review only - http://bmjopen.bmj.com/site/about/guidelines.xhtml

tumour patients, compared to placebo, as add-on to the treatment with dexamethasone, on the development of neuropsychiatric adverse effects that can be caused by dexamethasone.

For peer review only

For peer review only - http://bmjopen.bmj.com/site/about/guidelines.xhtml

METHODS ANDS ANALYSIS

This manuscript is written according to SPIRIT Standard Protocol Items: Recommendations for Interventional Trials (supplementary file S1) (22, 23).

Study design and setting

The DEXA-CORT study is a multicentre, randomized, double-blind, placebo-controlled trial investigating the effect of hydrocortisone replacement as co-treatment to reduce perioperative dexamethasone related neuropsychiatric adverse effects in patients with brain tumours undergoing elective neurosurgery. A total of 180 patients will be randomised in a 1:1 ratio, stratified per study centre and on tumour type. With randomisation we intend to equally distribute factors that can have an influence on our main outcome; neuropsychiatric effects. For example, the underlying pathology, the location of the tumour, extent of the surgery, and psychiatric history. The participating centres are Leiden University Medical Center (LUMC), Erasmus University Medical Center (Erasmus MC), Haaglanden Medical Center (HMC), and University Medical Center Utrecht (UMCU). The primary outcome measure is the proportion of patients with neuropsychiatric symptoms 5 days after surgery – or earlier if discharge is within 5 days – compared between the hydrocortisone and placebo treated group. The study is investigator-initiated with the LUMC as sponsor, and is funded by ZonMw and the Dutch Brain Foundation.

Eligibility criteria

Adult patients (≥18y) diagnosed with a brain tumour (glioma or meningioma) and undergoing elective surgical resection are eligible for inclusion. All patients receive high dose dexamethasone according to institutional protocols. Since peri-operative dexamethasone dosing schemes differ among the participating centres a minimal peri-operative cumulative dexamethasone dose of 24mg or more in 6 days is required. Initially, preoperative dexamethasone of more than one day was considered an exclusion criterium. During the course of the study it appeared that many patients, especially those with malignant tumours and severe oedema, had dexamethasone prescribed several days before the planned surgery. Consequently many patients were not eligible for study participation, threatening completion of inclusion. Hence this exclusion criterium was discarded with consent of the Data Safety Monitoring Board (DSMB). Detailed inclusion and exclusion criteria are presented in Figure 2.

Study outline

A timeline of the study is shown in Figure 3. Eligible patients are approached in an outpatient setting by specialized nurses in consultation with the treating neurosurgeon. The study is explained and

For peer review only - http://bmjopen.bmj.com/site/about/guidelines.xhtml

written information is provided (supplementary file S2 and S3). After written informed consent by the coordinating investigator or principle investigators, patient characteristics (age, gender, educational level, medical background, medication use, tumour type and tumour location) are recorded. The informed consent form includes consent for collection for blood sample and residual tumour tissue. Before start of intervention, a baseline study visit is planned that includes a structured psychiatric interview, different self-report questionnaires, and neurocognitive tests. A selection of the questionnaires can be filled out online via the web-based database program Castor Electronic Data Capture (EDC; Castor, Amsterdam, The Netherlands). The intervention starts on the day of hospital admission, concurrent with the dexamethasone treatment one day prior to planned surgery. 5 days postoperatively – or earlier at discharge – a structured psychiatric interview and neurocognitive screening are conducted, and questionnaires are filled out. The study medication is provided to the patients according to clinical care and is continued as long as dexamethasone is prescribed. A follow-up visit is planned 5 to 8 weeks postoperatively coinciding with the regular care assessment, at which a structured psychiatric interview and neurocognitive tests are performed and different questionnaires are filled out. Study visits take place at the medical centre of treatment. 2 weeks and 3 months postoperatively questionnaires can be filled out online via Castor EDC. Study duration from start of intervention until completion of the last questionnaires is 3 months. The study schedule is shown in Table 1. In case the baseline study visit is planned the day before surgery, no neurocognitive tests will be performed – instead a neurocognitive screening may be performed – and presentation of the questionnaires is depend on the patients' psychological and physical condition. To improve adherence the follow-up visits are coinciding with the regular care assessments.

Intervention

Patients will be treated according to routine clinical care, based on local treatment protocols. The investigational product is hydrocortisone with placebo as comparator, both produced by Tiofarma B.V., Oud-Beijerland, The Netherlands. The Clinical Pharmacy and Toxicology department of the LUMC will repack, label and randomize the study medication. 180 patients are randomised between treatment with either twice daily 10mg hydrocortisone or placebo, both orally administered concurrent with the full course of perioperative dexamethasone treatment; mostly with a dexamethasone taper schedule. Study medication is available for 20 days; assuming the slow dexamethasone taper schedule is used, treatment will continue on average for 15 days. In the event that dexamethasone treatment re-starts after completion of its taper schedule, study medication will not be available. The study medication is prescribed by the physician and administration details are recorded. Treatment dosage is targeted towards physiological cortisol replacement. During

For peer review only

For peer review only - http://bmjopen.bmj.com/site/about/guidelines.xhtml

hospitalisation the hydrocortisone or placebo is administered in the morning at 08:00hr and in the evening between 18:00hr and 20:00hr, because at these times dexamethasone is administered according to local standard procedures. To minimise the number of errors in medicine administrations we decided to adhere to the existing logistics. When participants go home and have to taper the dexamethasone, they receive a taper schedule from the nurses. Depending on the schedule, they receive further instructions on the hydrocortisone/placebo administration (both verbally and on paper). For manageability reasons we decided to always take the hydrocortisone/placebo at the first and the last time point of the dexamethasone administration. Study medication and information is handed out to the patients by the nurses, in consultation with the coordinating investigator. Patients will receive the amount of study medication they need to minimise medication errors. Drug accountability is checked by the coordinating investigator.

Allocation, randomisation and blinding

Randomisation is controlled by the Clinical Pharmacy and Toxicology department of LUMC using block randomisation in a 1:1 ratio stratified for each medical centre participating in the study, and for tumour type (glioma or meningioma) with block size of 10. New study participants are assigned to a randomisation number by the coordinating investigator. The coordinating investigator will contact the local pharmacy about the patient and its randomisation number. The local pharmacy assigns the randomisation number to a medication number and then the pharmacists know the treatment allocation. The randomisation list is stored at the department and will not be disclosed until all participants have completed the trial. Only in case of emergency and the knowledge of the assigned treatment is required the randomisation code can be broken. The study is blinded for everyone involved, except the pharmacy who allocates participants to treatment. In addition, hydrocortisone and placebo pills are identical in terms of appearance and taste ensuring blinding.

Assessments during the study

Assessments during the study include neuropsychiatric symptoms, quality of sleep, neurocognitive assessment, registration of (serious) adverse events ((S)AEs), prescribed medication, and referrals to psychiatrist/psychologist. We plan to evaluate potential long term follow-up effects on health-related quality of life (HRQoL). However, we did not include a specific measure for PTSD, even if a 'planned critical illness' like elective brain surgery may induce this disease (24). Furthermore, blood and residual tissue from tumour specimens will be collected to verify MR targeting and molecular effects of hydrocortisone co-treatment.

For peer review only - http://bmjopen.bmj.com/site/about/guidelines.xhtml

Neuropsychiatric symptoms

Brief Psychiatric Rating Scale (BPRS)

Neuropsychiatric symptoms will be measured with the BPRS version 4.0 (25). The BPRS is an interview, which provides a description of major symptom characteristics in psychiatric patients. The BPRS covers the presence and severity of 24 symptoms scored on a 7 point scale (1 = not present, 7 = extremely severe). Possible scores vary from 24 to 168. The BPRS will be used for our primary outcome.

Mini-screen

A mini-screen will be used to determine the patient's neuropsychiatric history. This mini-screen comprises 22 'Yes' or 'No' questions about different neuropsychiatric symptoms (26).

Hospital Anxiety and Depression Scale (HADS)

To determine the levels of anxiety and depression in patients, the HADS questionnaire will be used (27). The HADS comprises 14 items; seven related to anxiety and seven to depression. Each question has four different answer options, ranging from 0 to 3. Patients scoring 8-10 on the anxiety or depression domain are classified as possibly suffering from anxiety or depression, respectively, and a score ≥11 as definite anxiety or depression.

Positive and Negative Affect Schedule (PANAS)

To determine the positive and negative affect of patients the PANAS is used. The PANAS comprises a 20-item mood scale yielding 2 subscales; positive affect (PA) and negative affect (NA) (28). Patients rate how they felt 'in general' on a 5-point scale; 1 = very slightly or not at all, 2 = a little, 3 = moderately, 4 = quite a bit and 5 = very much. PA scores range from 10-50, with higher scores representing higher levels of positive affect. NA scores range form 10-50, with lower scores representing lower levels of negative affect.

The Altman Self-Rating Mania Scale (ASRM)

The ASRM is used to assess the presence and/or severity of manic symptoms (29). The ASRM consists of 5-items consisting of 5 statements. At the 5 items, the patient chooses one statement that best describes the way he or she has been feeling for the past week.

Neuropsychiatric Inventory Question (NPI-Q)

For peer review only - http://bmjopen.bmj.com/site/about/guidelines.xhtml

The NPI-Q is a questionnaire to be filled out by a family member or care-taker of the patient, that specifically assesses neuropsychiatric symptoms of the patient in the last month (30-32). The NPI-Q consist of 12 domains of symptoms. Every domain needs to be answered with 'yes' (is present) or 'no' (is absent). When the symptom is present, the partner rates the severity of the symptom (3-point scale) and the psychiatric burden (6-point scale) for him/herself. The NPI-Q gives a total score for the severity (range 0 – 36) and a total score for emotional burden (range 0 – 60).

<u>Delirium Observation Screening (DOS)</u>

The DOS scale is used to determine the presence of delirium in patients and includes 13 observation items of behaviour that show symptoms of delirium (33). Nurses measure the different items during the day, evening and night. Each item has 3 answer options; 'never', 'sometimes – always' and 'not known'. These 3 scores are then scored with 0, 1 or no points. The total DOS scale score is the sum of the total DOS score at day, evening and night divided by 3. Patients scoring $\geq$3 are probably suffering from delirium.

Quality of sleep

To determine the sleep quality the Leeds Sleep Evaluation Questionnaire (LSEQ) is used (34, 35). LSEQ contact information and permission to use is available from Mapi Research Trust, Lyon, France, https://eprovide.mapi-trust.org. LSEQ is a reliable instrument to assess sleep quality changes during psycho-pharmacological treatment intervention. This questionnaire is a self-report measure with 10-items consisting of a 10-cm-line visual analogue rating scale that evaluates four domains; ease of initiating sleep, quality of sleep, ease of waking and behaviour following wakefulness. The LSEQs 10-cm-horizontal line has 2 extreme states defined at the end of the line (e.g. Tired = score of 0, Alert = score of 10). Patients respond by placing a vertical mark on the line. Each question is scored out of 10 (e.g. 0 cm = score 0,5 cm = score of 5).

HRQoL

HRQoL will be measured with the EORTC core quality of life questionnaire (QLQ-C30) complemented with the QLQ-BN20 (36, 37). The QLQ-C30 is specific in assessing the HRQoL of cancer patients participating in clinical trials. It consists of 30 items, of which 28 have a four-point scale (1 = not at all, 4 = very much). 2 questions have a seven-point scale (1 = very poor, 7 = excellent). A high score represents a higher response level; high/healthy level of functioning, high QoL and a high level of symptoms. The QLQ-BN20 is a validated module specific for patients with brain neoplasms. It includes

For peer review only

For peer review only - http://bmjopen.bmj.com/site/about/guidelines.xhtml

20 items with a four-point scale (1 = not at all, 4 = very much) and the higher the score the more symptoms or problems.

Neurocognitive assessment

To assess the cognitive domains verbal memory, executive functioning and attention, we used the following standardized neurocognitive tests: HVLT-R – Free Recall (38), Trail Making Test A and B (39), Controlled Oral Word Association (40) and MOS Scale (41). Raw scores can be transformed into z-scores in order to compare patients' performance to control participants (and can be corrected for demographic variables, such as age and education).

The Montreal Cognitive Assessment (MoCA) is a cognitive screening instrument for mild cognitive dysfunction (42). It measures different cognitive domains; attention and concentration, executive functions, memory, language, visuo-constructional skills, conceptual thinking, calculations and orientation. The total score is 30 points; a score of ≥26 is a normal score.

The Aphasia Bedside Check (ABC) is a 10-min screening tool that globally determines the presence of aphasia with seven items for language comprehension and seven items for language production (43). This screening will only be performed in patients who have a left sided brain tumour with (hetero-) anamnestic language problems. With ≥3 mistakes an aphasia may be present and the patient should not be included in the study.

(Serious) Adverse Events ((S)AEs)– Medication – Referral to psychiatrist/psychologist

(S)AEs, prescribed medication (especially psychiatric medication) and referrals or evaluations of psychiatrist/psychologist will be recorded.

Laboratory measures

We try to collect 1 tube of blood taken during anaesthetic procedures for surgery. This will be used for DNA analyses to determine MR haplotype. Additionally, cortisol and glucose levels will be measured in blood.

If possible during the surgical procedure tumour tissue and peritumoral infiltrated brain tissue will be collected to investigate MR and GR and the effect of cortisol on these receptors.

Concomitant care and trial follow-up

Patient care is continued as usual. Participants are allowed to participate in other trials if they are not interfering with our intervention period. Participants may withdraw from the study for any reason at

For peer review only - http://bmjopen.bmj.com/site/about/guidelines.xhtml

any time. Premature study withdrawal will have no effect on regular patient care. All data collected up to withdrawal will be used for analysis. All participants will be followed until completion of the trial and will be notified of the results.

Primary outcome

The primary study outcome is the difference in proportion of patients scoring ≥3 points on at least one of the BPRS questions 5 days postoperatively – or earlier at discharge – between the hydrocortisone and placebo group. A better outcome on the BPRS in the hydrocortisone group would be considered a positive result, which would indicate that addition of hydrocortisone to dexamethasone is beneficial to reduce neuropsychiatric adverse effects of dexamethasone.

Secondary outcomes

Secondary outcomes are neuropsychiatric symptoms, quality of sleep, HRQoL, and neurocognitive functioning 5 days postoperatively – or earlier at discharge – , 2 weeks, 5 to 8 weeks, and 3 months postoperatively. Furthermore, neuropsychiatric history, SAEs, prescribed (psychiatric) medication and referrals or evaluations of psychiatrist/psychologist will be collected. In addition, the laboratory measurements are considered secondary outcomes.

Sample size

The sample size calculation is based on the results of a pilot study in which we tested the BPRS in our study population. We determined postoperative BPRS scores to evaluate the presence of psychiatric symptoms in brain tumour patients who underwent surgery and were treated with dexamethasone. We interviewed 18 patients on the day of hospital discharge. According to the BPRS scores in this group we considered a score of ≥3 on at least one of the BPRS items as clinically relevant for patients. We found that 7 out of 18 patients (≈40%) scored at least ≥3 on one of the BPRS items. Based on expert clinical judgement a 50% reduction in the patients with a score ≥3 is perceived as relevant. This equals an absolute difference of 20%. If we consider an $\alpha = 0.05$ and a power of 80%, and we take the 20% absolute difference between the 2 treatment arms, a sample size of 82 patients in each group would be needed. Based on an expected drop-out rate of ≈10%, 90 patients in each treatment arm (in total 180 patients) will be considered as a satisfactory sample size.

Planned statistical analyses

This study is a randomized controlled trial. All data will be analysed on an intention-to-treat basis.

BPRS

For peer review only - http://bmjopen.bmj.com/site/about/guidelines.xhtml

The primary study parameter is the proportion of patients scoring ≥3 points on at least one of the BPRS questions 5 days postoperatively – or earlier at discharge. The difference in these proportions between the placebo group and the hydrocortisone group will be calculated and statistically compared with the Chi-Square test. Any difference with a $p<0.05$ will be considered significant.

As secondary analyses, both treatment arms will be compared over time using analysis of repeated measures, generalized estimating equation (GEE) for the dichotomous outcomes as described in the primary outcome and the mixed linear model for the BPRS as continuous outcome. Additionally, we will look at the differences between the treatment arms on the separate items of the BRPS.

Mini-screen, HADS, PANAS, ASRM, NPI-Q, DOS, LSEQ and HRQoL

Both treatment arms will be compared over time using analysis of repeated measures, GEE when outcome is dichotomous and mixed linear models for continuous outcomes.

Explanatory univariable and multivariable regression analysis will be used to identify (baseline) predictors of treatment response. Sensitivity analysis will be performed using multiple imputation to handle missing data. Any difference with a $p<0.05$ will be considered significant. A better outcome on the different scales in the hydrocortisone group would be considered a positive result.

Neurocognitive functioning

The neurocognitive tests are a combination of neuropsychological tests for which normative data were published, so that patient's performance can be corrected according to age, gender and education. We will compare the raw data between the treatment arms with a t-test. Test scores can be transformed into z-values using normative data and compared with a one sample t-test. With no-normal distributions the appropriate statistical tests will be used.

Subgroup analyses

Subgroup analyses will be performed to explore differences in our primary and secondary parameters for type of tumour, gender, age and dosage of dexamethasone.

As the study remains blinded until completion, no interim analyses are planned. In addition, the statistical plan will be finalized before starting the analyses.

Data management

All data will be collected, stored, and analysed in a coded manner, and in accordance with the General Data Protection Regulation (GDPR). Data are collected and stored in the web-based database

For peer review only

For peer review only - http://bmjopen.bmj.com/site/about/guidelines.xhtml

management system Castor EDC using electronic case report forms (CRFs). The CRFs have built-in data validity and value range checks. Source documents include medical records, paper CRFs, paper interviews, questionnaires and neuropsychological tests. All data is pseudonymised with a study code and will be stored for 15 years. The subject identification log which links subjects to the code is kept in a trial file only accessible to study personnel. Possible blood samples and residual tumour tissue obtained for standard care and obtained following local consent forms during the study will be stored according to local regulatory guidelines.

Safety

Participating in the trial is very unlikely to bring additional risks for patients, since hydrocortisone is given in a low dose to restore the physiological cortisol level in the body.

DSMB

This study established a DSMB that protects and guarantees patients safety during the trial. The DSMB includes 3 members with different study backgrounds: a statistician, psychiatrist and an internist/clinical epidemiologist. The role and responsibilities of the DSMB is described in the DSMB charter document.

AEs and SAEs

The study population has a high risk of complications, which are inherent to their vulnerable condition (neurological deficits caused by a brain tumour, surgery, high dose dexamethasone) and these are considered unrelated to the intervention. The patient population is expected to encounter many AEs not related to the experimental design, therefore not all AE will be registered. AEs that will be registered are: psychiatric complaints of which psychiatric consultation and medication is needed, brain oedema for which increased dexamethasone medication is given, or for which the dexamethasone reduction scheme is extended, all infections for which antibiotics are needed, cerebrospinal fluid (CSF) leakage that requires surgical intervention, hyperglycaemia for which medication is needed, cardiovascular events requiring intervention, epileptic seizures requiring intervention and secondary adrenal insufficiency that requires hydrocortisone suppletion. The abovementioned AEs will be registered from the start of the intervention until completion of the trial.

SAEs that we will record are any untoward medical occurrence or effect that results in death, is life threatening (at the time of the event), requires hospitalisation or prolongation of existing inpatients' hospitalisation – including intensive care – when a patient has unexpected longer IC admission or unexpected >10 days hospitalisation, results in persistent or significant disability or incapacity – like

For peer review only - http://bmjopen.bmj.com/site/about/guidelines.xhtml

For peer review only

366 neurological dysfunction – lasting for more than 3 days postoperatively, results in any other important medical event not mentioned above due to medical or surgical intervention and judged as such by the investigators and post-operative haemorrhage for which re-craniotomy is needed. An elective hospital admission will not be considered a SAE. Whether related to the study medication or not, all these SAEs occurring during the intervention period will be reported through the web portal governed and issued by the Dutch National Ethics Committee (CCMO) called 'ToetsingOnline'. SAEs occurring after the intervention period will be line-listed and not reported in ToetsingsOnline.

Monitoring

According to Good Research Practice (GRP) independent on-site monitoring visits will be performed to ensure the conduct of the trial is consistent with the approved protocol(s), the rights and well-being of the subjects are protected, source documents are verified and data is accurately reported from the source documents. Monitoring visits will take place according to the study specific monitoring plan.

Patients and public involvement

Patients and public were not involved in the study design. The ZonMw committee that granted the funding includes a representative of the patients interests. The committee is kept informed annually.

For peer review only

ETHICS AND DISSEMINATION

The study protocol has been approved by the accredited Medical Research Ethics Committee of the LUMC and by the Dutch competent authority. Permission for execution of the study is obtained at each participating site. Substantial amendments to the protocol will first be sent for approval and upon consent the participating centres and trial registries will be informed. The study will be conducted according to the principles of the Declaration of Helsinki, Good Clinical Practice guidelines – including centre specific requirements – and in accordance with the Dutch Medical Research Involving Human Subjects Act (WMO). Participant WMO subject insurance is provided by the LUMC for all participating subjects and liability insurances are provided by all participating centres. This trial is registered in the Dutch Trial Register (NTR). Regardless of the outcome, trial results will be submitted for publication in a peer-reviewed journal and presented at conferences. Publication rights and ownership of data is secured in a clinical trial agreement with participating centres. Full protocol, data sets and statistical analyses will be available in a repository with a persistent identifier after publication of the final results.

Trial status

The first patient was included on 10 September 2019 and recruitment is ongoing. Inclusion rate has slowed down due to the COVID-19 pandemic from March 2020 onwards. End of recruitment is expected to be 2022. The current study protocol version is V3, August 28. 2020.

For peer review only

For peer review only - http://bmjopen.bmj.com/site/about/guidelines.xhtml

DISCUSSION

The DEXA-CORT trial is the first double-blind, placebo-controlled trial in adult brain tumour patients, in which we investigate the possible prevention of neuropsychiatric adverse effects of perioperative dexamethasone treatment. Dexamethasone is a well-known very potent activator of the GR and consequently suppresses endogenous adrenocortical cortisol production via negative feedback on the HPA-axis, depleting brain MR from its ligand (12-14). The resultant disturbed balance in receptor activation in the brain may predispose for psychopathology. In this study, we test the hypothesis that re-instating brain MR activity via co-treatment with a physiological dose of cortisol (=hydrocortisone) will improve (specific features of) neuropsychological well-being. Because of the high dexamethasone doses and high affinity of dexamethasone for GR, cortisol treatment will only affect MR occupancy and not interfere with the therapeutic goal of dexamethasone treatment, which is GR activation.

Adverse neuropsychiatric effects can also be present with the use of other GCs, like prednisone and prednisolone. Like dexamethasone, prednisone and prednisolone suppress the HPA-axis, which would subsequently deplete MR from its ligand. In addition, compared to the naturally produced cortisol, the synthetic GCs dexamethasone, prednisone, and prednisolone all have much lower MR binding potency (11). An important difference between dexamethasone and the other mentioned GCs is its higher GR potency, its long half-life and lower MR potency. The potency differences could be of influence on the adverse effects; higher GR potency could maybe suppress the HPA-axis faster and longer half-life might extend its effects. Lower MR potency might accentuate the effect of a suppressed HPA-axis. It would be interesting to investigate the difference in neuropsychiatric effects between different GCs, and (eventually) benefit of hydrocortisone add on.

This cortisol re-fill approach was already performed in childhood leukaemia patients. Patients who experienced severe adverse effects of dexamethasone seemed to benefit from the additional hydrocortisone medication (21). To establish this potential positive effect in an adult population, the DEXA-CORT trial is investigating this principle in adult brain tumour patients. Dexamethasone is a standard clinical treatment in brain tumour patients perioperatively and adverse effects are also present in this patient group, which leads to reduced HRQoL. A methodological drawback of our study population is the lack of a cross-over design. Rather, the high doses, but brief perioperative use of dexamethasone formed the rational for testing our hypothesis in this patient group. If additional hydrocortisone to dexamethasone proves effective in reducing its neuropsychiatric adverse effects, it would offer a new treatment option for this patient group and maybe even for many other patients that receive long-term treatment with dexamethasone, and other synthetic GCs. An interesting question that will follow is how long to continue the hydrocortisone replacement. After

For peer review only - http://bmjopen.bmj.com/site/about/guidelines.xhtml

dexamethasone treatment, the endogenous HPA-axis will need a recovery period. It might be beneficial to continue the additional hydrocortisone for at least some days after dexamethasone is stopped.

For peer review only

For peer review only - http://bmjopen.bmj.com/site/about/guidelines.xhtml

Supplementary files

Supplementary file S1: Completed SPIRIT Checklist.

Supplementary file S2: Patient information letter and informed consent, Dutch LUMC version.

Supplementary file S3: Patient information letter and informed consent, English LUMC version.

Footnotes

Contributors: OCM was main applicant in the funding acquisition. ACAM, DDS, AHZN, NRB, EFCR, CHV, CMFD, AMP, WRF and OCM participated in the design of the study. CMFD, DDS, AMP, WRF and RDSN are principal investigators at the participating study centres. CMFD, DDS, RDSN, WAM, ACAM initiated and implemented the study design at the participating study centre. ACAM is the coordinating investigator with DDS as daily supervisor. ACAM drafted the manuscript. All authors reviewed and approved the final manuscript.

Funding: This work is supported by The Netherlands Organisation for Health Research and Development (ZonMw) and Dutch Brain Foundation grant project number 95105005. The DEXA-CORT study is investigator-initiated; Leiden University Medical Center (LUMC) is the sponsor of the study. The funding party did play no role in the final study design or study conduct nor in the manuscript preparation of decision to submit for publication.

Competing interests: Authors have nothing to disclose.

Ethics approval: Medical Research Ethics Committee of the LUMC (reference: NL63350.058.18)

Trial registration: EudraCT: 2017-003705-17, registered 12 September 2017; Netherlands Trial Register (NTR): NL6726, registered 18 January 2018. Registration took place before start of recruitment.

Protocol versions:

- V2.0, February 28 2019: first approved protocol version
- V3.0, August 28 2020: study medication use changed from 10 to 15 days, addition of informed consent form for the partner/family member of the participant. Small protocol changes: better description of tracking of AE and SAE, acceptance of 1 mistake in study medication administration and replacing excluded study participants. Also extension of study duration.

Data sharing statement: The datasets generated and/or analysed during the current study will be available in a repository with a persistent identifier after publication of the final results.

For peer review only - http://bmjopen.bmj.com/site/about/guidelines.xhtml

REFERENCES

1. Ismail MF, Lavelle C, Cassidy EM. Steroid-induced mental disorders in cancer patients: a systematic review. Future Oncol. 2017;13(29):2719-31.

2. Hockey B, Leslie K, Williams D. Dexamethasone for intracranial neurosurgery and anaesthesia. J Clin Neurosci. 2009;16(11):1389-93.

3. Arvold ND, Armstrong TS, Warren KE, Chang SM, DeAngelis LM, Blakeley J, et al. Corticosteroid use endpoints in neuro-oncology: Response Assessment in Neuro-Oncology Working Group. Neuro Oncol. 2018;20(7):897-906.

4. Strehl C, Bijlsma JW, de Wit M, Boers M, Caeyers N, Cutolo M, et al. Defining conditions where long-term glucocorticoid treatment has an acceptably low level of harm to facilitate implementation of existing recommendations: viewpoints from an EULAR task force. Ann Rheum Dis. 2016;75(6):952-7.

5. Judd LL, Schettler PJ, Brown ES, Wolkowitz OM, Sternberg EM, Bender BG, et al. Adverse consequences of glucocorticoid medication: psychological, cognitive, and behavioral effects. Am J Psychiatry. 2014;171(10):1045-51.

6. Wen PY, Schiff D, Kesari S, Drappatz J, Gigas DC, Doherty L. Medical management of patients with brain tumors. J Neurooncol. 2006;80(3):313-32.

7. Galicich JH, French LA, Melby JC. Use of dexamethasone in treatment of cerebral edema associated with brain tumors. J Lancet. 1961;81:46-53.

8. Dietrich J, Rao K, Pastorino S, Kesari S. Corticosteroids in brain cancer patients: benefits and pitfalls. Expert Rev Clin Pharmacol. 2011;4(2):233-42.

9. Pelletier G, Verhoef MJ, Khatri N, Hagen N. Quality of life in brain tumor patients: the relative contributions of depression, fatigue, emotional distress, and existential issues. J Neurooncol. 2002;57(1):41-9.

10. Fardet L, Petersen I, Nazareth I. Suicidal behavior and severe neuropsychiatric disorders following glucocorticoid therapy in primary care. Am J Psychiatry. 2012;169(5):491-7.

11. Grossmann C, Scholz T, Rochel M, Bumke-Vogt C, Oelkers W, Pfeiffer AF, et al. Transactivation via the human glucocorticoid and mineralocorticoid receptor by therapeutically used steroids in CV-1 cells: a comparison of their glucocorticoid and mineralocorticoid properties. Eur J Endocrinol. 2004;151(3):397-406.

12. Spencer RL, Deak T. A users guide to HPA axis research. Physiol Behav. 2017;178:43-65.

13. De Kloet R, Wallach G, McEwen BS. Differences in corticosterone and dexamethasone binding to rat brain and pituitary. Endocrinology. 1975;96(3):598-609.

14. Karssen AM, Meijer OC, Berry A, Sanjuan Pinol R, de Kloet ER. Low doses of dexamethasone can produce a hypocorticosteroid state in the brain. Endocrinology. 2005;146(12):5587-95.

15. Meijer OC, de Lange EC, Breimer DD, de Boer AG, Workel JO, de Kloet ER. Penetration of dexamethasone into brain glucocorticoid targets is enhanced in mdr1A P-glycoprotein knockout mice. Endocrinology. 1998;139(4):1789-93.

16. Meijer OC, de Kloet ER. A Refill for the Brain Mineralocorticoid Receptor: The Benefit of Cortisol Add-On to Dexamethasone Therapy. Endocrinology. 2017;158(3):448-54.

17. ter Heegde F, De Rijk RH, Vinkers CH. The brain mineralocorticoid receptor and stress resilience. Psychoneuroendocrinology. 2015;52:92-110.

18. Klok MD, Giltay EJ, Van der Does AJ, Geleijnse JM, Antypa N, Penninx BW, et al. A common and functional mineralocorticoid receptor haplotype enhances optimism and protects against depression in females. Transl Psychiatry. 2011;1:e62.

19. Otte C, Hinkelmann K, Moritz S, Yassouridis A, Jahn H, Wiedemann K, et al. Modulation of the mineralocorticoid receptor as add-on treatment in depression: a randomized, double-blind, placebo-controlled proof-of-concept study. J Psychiatr Res. 2010;44(6):339-46.

For peer review only - http://bmjopen.bmj.com/site/about/guidelines.xhtml

20. Otte C, Wingenfeld K, Kuehl LK, Kaczmarczyk M, Richter S, Quante A, et al. Mineralocorticoid receptor stimulation improves cognitive function and decreases cortisol secretion in depressed patients and healthy individuals. Neuropsychopharmacology. 2015;40(2):386-93.
21. Warris LT, van den Heuvel-Eibrink MM, Aarsen FK, Pluijm SM, Bierings MB, van den Bos C, et al. Hydrocortisone as an Intervention for Dexamethasone-Induced Adverse Effects in Pediatric Patients With Acute Lymphoblastic Leukemia: Results of a Double-Blind, Randomized Controlled Trial. J Clin Oncol. 2016;34(19):2287-93.
22. Chan AW, Tetzlaff JM, Altman DG, Laupacis A, Gotzsche PC, Krleza-Jeric K, et al. SPIRIT 2013 Statement: Defining Standard Protocol Items for Clinical Trials. Ann Intern Med. 2013;158(3):200-+.
23. Chan AW, Tetzlaff JM, Gotzsche PC, Altman DG, Mann H, Berlin JA, et al. SPIRIT 2013 explanation and elaboration: guidance for protocols of clinical trials. Bmj-Brit Med J. 2013;346.
24. Franks ZM, Alcock JA, Lam T, Haines KJ, Arora N, Ramanan M. Physical Restraints and Post-Traumatic Stress Disorder in Survivors of Critical Illness A Systematic Review and Meta-analysis. Ann Am Thorac Soc. 2021;18(4):689-97.
25. Ventura J, Lukoff D, Nuechterlein KH. Brief Psychiatric Rating Scale (BPRS) Expanded Version (4.0): Scales, Anchor Points and Administration Manual. . International Journal of Methods in Psychiatric Research. 1993;3:227-44.
26. Sheehan DV, Lecrubier Y, Sheehan KH, Amorim P, Janavs J, Weiller E, et al. The Mini-International Neuropsychiatric Interview (M.I.N.I.): the development and validation of a structured diagnostic psychiatric interview for DSM-IV and ICD-10. J Clin Psychiatry. 1998;59 Suppl 20:22-33;quiz 4-57.
27. Zigmond AS, Snaith RP. The hospital anxiety and depression scale. Acta Psychiatr Scand. 1983;67(6):361-70.
28. Watson D, Clark LA, Tellegen A. Development and validation of brief measures of positive and negative affect: the PANAS scales. J Pers Soc Psychol. 1988;54(6):1063-70.
29. Altman EG, Hedeker D, Peterson JL, Davis JM. The Altman Self-Rating Mania Scale. Biol Psychiatry. 1997;42(10):948-55.
30. Cummings JL, Mega M, Gray K, Rosenberg-Thompson S, Carusi DA, Gornbein J. The Neuropsychiatric Inventory: comprehensive assessment of psychopathology in dementia. Neurology. 1994;44(12):2308-14.
31. Kaufer DI, Cummings JL, Ketchel P, Smith V, MacMillan A, Shelley T, et al. Validation of the NPI-Q, a brief clinical form of the Neuropsychiatric Inventory. J Neuropsychiatry Clin Neurosci. 2000;12(2):233-9.
32. de Jonghe JF, Kat MG, Kalisvaart CJ, Boelaarts L. [Neuropsychiatric inventory questionnaire (NPI-Q): A validity study of the Dutch form]. Tijdschr Gerontol Geriatr. 2003;34(2):74-7.
33. Schuurmans MJ, Shortridge-Baggett LM, Duursma SA. The Delirium Observation Screening Scale: a screening instrument for delirium. Res Theory Nurs Pract. 2003;17(1):31-50.
34. Parrott AC, Hindmarch I. Factor analysis of a sleep evaluation questionnaire. Psychol Med. 1978;8(2):325-9.
35. Hindmarch I. A 1,4-benzodiazepine, temazepam (K 3917), its effect on some psychological parameters of sleep and behaviour. Arzneimittelforschung. 1975;25(11):1836-9.
36. Aaronson NK, Ahmedzai S, Bergman B, Bullinger M, Cull A, Duez NJ, et al. The European Organization for Research and Treatment of Cancer QLQ-C30: a quality-of-life instrument for use in international clinical trials in oncology. J Natl Cancer Inst. 1993;85(5):365-76.
37. Taphoorn MJ, Claassens L, Aaronson NK, Coens C, Mauer M, Osoba D, et al. An international validation study of the EORTC brain cancer module (EORTC QLQ-BN20) for assessing health-related quality of life and symptoms in brain cancer patients. Eur J Cancer. 2010;46(6):1033-40.
38. Brandt J, Benedict RHB. HVLT-R: Hopkins Verbal Learning Test-Revised. Copyright 1991, 1998, 2001 by PAR, Inc
39. Tombaugh TN. Trail Making Test A and B: normative data stratified by age and education. Arch Clin Neuropsychol. 2004;19(2):203-14.

For peer review only - http://bmjopen.bmj.com/site/about/guidelines.xhtml

40. Benton AL, Hamsher KdS. Multilingual Aphasia Examination. Copyright 1978 by PAR, Inc.
41. Stewart AL, Greenfield S, Hays RD, Wells K, Rogers WH, Berry SD, et al. Functional Status and Well-Being of Patients with Chronic Conditions - Results from the Medical Outcomes Study. Jama-J Am Med Assoc. 1989;262(7):907-13.
42. Nasreddine ZS, Phillips NA, Bedirian V, Charbonneau S, Whitehead V, Collin I, et al. The Montreal Cognitive Assessment, MoCA: a brief screening tool for mild cognitive impairment. J Am Geriatr Soc. 2005;53(4):695-9.
43. Visch-Brink E, El Hachioui H. *Afasie Bedside Check*. Erasmus MC, Rotterdam, Nederland. 2013.

For peer review only

For peer review only - http://bmjopen.bmj.com/site/about/guidelines.xhtml

TABLES

Table 1. The DEXA-CORT study schedule of enrolment, intervention and assessments.

	**STUDY PERIOD**								
	**Outpatient clinic**	**Baseline**	**Treatment**				**Follow-up**	**Follow-up**	**Close-out**
			**admission**	**surgery**	**ward**	**discharge**			
**Days**		-14 – -1	-1	0	1 – 4	3 – 5	14	35 – 56	84
**Visits**	V_0	V_1	V_1/V_2					V_2/V_3	
**Enrolment:**									
Eligibility screen	X								
Informed consent		X	X						
Baseline characteristics		X	X						
Allocation		X	X						
**Interventions:**									
Hydrocortisone			X	→	→	→	→		
Placebo			X	→	→	→	→		
**Assessments:**									
BPRS		X	X			X		X	
Mini-screen		X	X						
HADS		X	(X)				X		X
PANAS		X	(X)		X	X	X	X	X
ASRM		X	(X)				X		X
NPI-Q		X	X			X		X	
DOS					X				
LSEQ		X	(X)			X	X	X	X
QoL-C30/BN20		X	(X)						X
Cognitive tests		X						X	
MoCA			(X)			X			
ABC		X	X			X			
SAEs			X	→	→	→	→		

For peer review only

AEs		X							X
Medication		X							X
Psychiatric referrals		X							X
**Laboratory measures:**									
Tube of blood				X					
Residual tumour tissue				X					

BPRS: Brief Psychiatric Rating Scale, HADS: Hospital Anxiety and Depression Scale, PANAS: Positive And Negative Affect Schedule, ASRM: Altman Self-Rating Mania Scale, NPI-Q: Neuropsychiatric Inventory Question, DOS: Delirium Observation Screening, LSEQ: Leeds Sleep Evaluation Questionnaire, QoL: Quality of Life questionnaires, MoCA: Montreal Cognitive Assessment, ABC: Aphasia Bedside Check, (S)AEs: (Serious) Adverse Events.

For peer review only - http://bmjopen.bmj.com/site/about/guidelines.xhtml

FIGURE LEGENDS

Figure 1: Preventing neuropsychiatric adverse effects of dexamethasone with co-administration of cortisol. Dexamethasone suppresses the HPA-axis and thereby the adrenocortical cortisol production, leaving cortisol-preferring MRs unoccupied. Re-activating the MR using cortisol might reduce or prevent neuropsychiatric adverse effects of dexamethasone treatment via activation of brain MR.

Figure 2: Eligibility criteria of the DEXA-CORT study.

Figure 3: The DEXA-CORT study timeline.

For peer review only

For peer review only - http://bmjopen.bmj.com/site/about/guidelines.xhtml

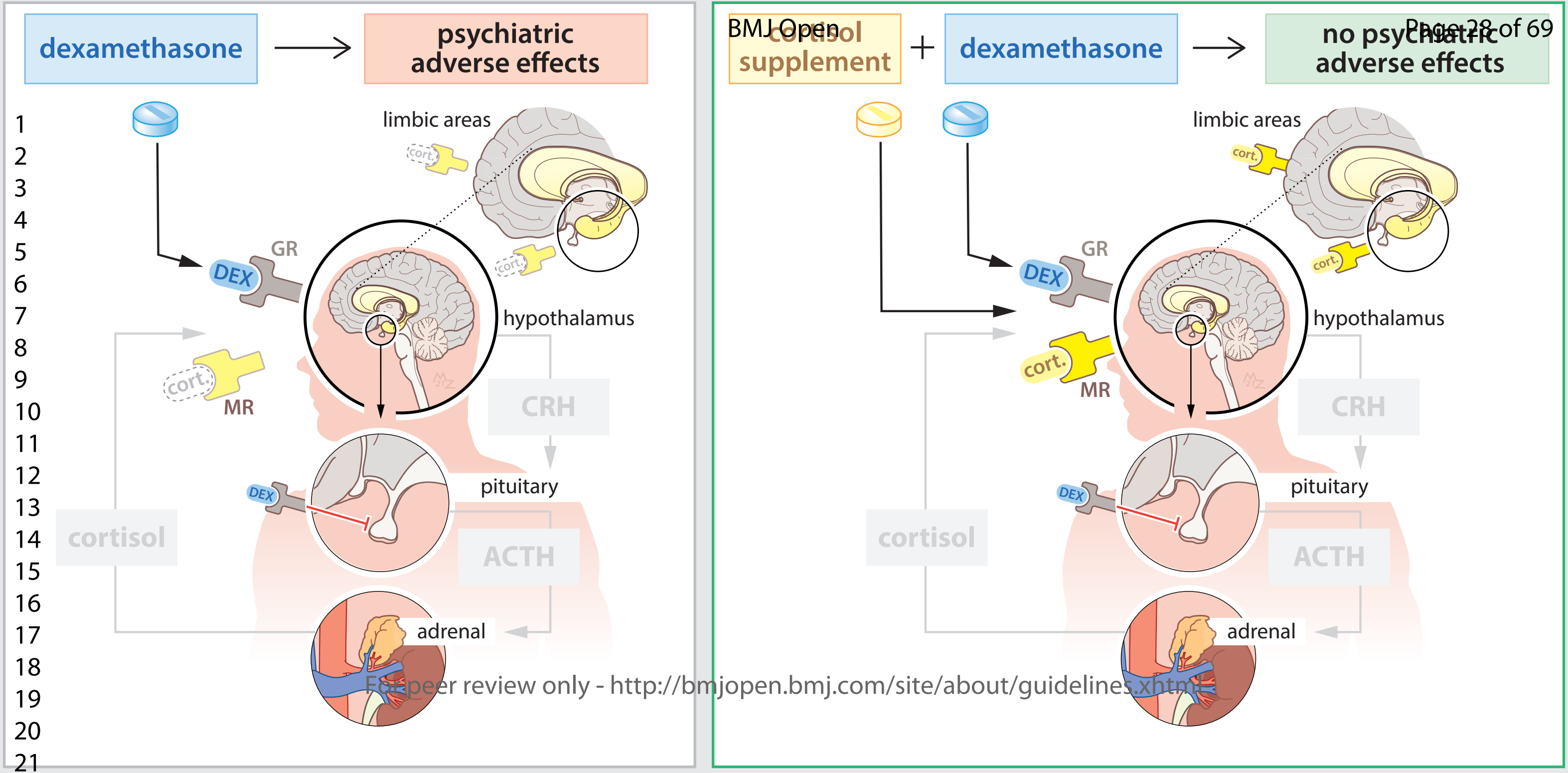
dexamethasone
psychiatric adverse effects
limbic areas
cort.
GR
DEX
hypothalamus
cort.
MR
CRH
pituitary
DEX
cortisol
ACTH
adrenal
cortisol supplement
+
dexamethasone
no psychiatric adverse effects
limbic areas
cort.
GR
DEX
hypothalamus
cort.
MR
CRH
pituitary
DEX
cortisol
ACTH
adrenal

Inclusion criteria:

- Age = ≥18 years
- Cranial glioma or meningioma patients scheduled to undergo surgery (resection)
- Minimal dose of peri-operative cumulative dexamethasone exposure of 24mg or more in 6 days
- Good clinical condition; KPS ≥70
- Life expectancy ≥ 6 months

Exclusion criteria:

- Non-native speakers of Dutch or insufficient command of the Dutch language
- Patients that are unable to overview consequences of trial participation
- Patients with severe aphasia
- Patients that are not able to fill in the questionnaires because of cognitive impairments at the discretion of the physician
- Patients with psychiatric diseases or neurological deficits that interfere with the study to the judgement of treating physician
- Patients with prior diagnosis of secondary adrenal insufficiency/already taking hydrocortisone

For peer review only

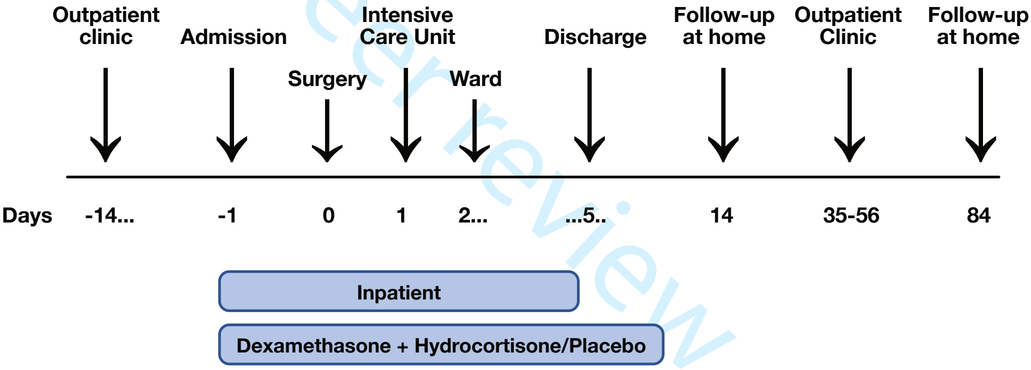

For peer review only - http://bmjopen.bmj.com/site/about/guidelines.xhtml

SPIRIT

STANDARD PROTOCOL ITEMS: RECOMMENDATIONS FOR INTERVENTIONAL TRIALS

Manuscript Koning *et al.* 'The DEXA-CORT trial: study protocol of a randomized placebo-controlled trial of hydrocortisone in brain tumour patients on the prevention of neuropsychiatric adverse effects caused by perioperative dexamethasone.'

For peer review only

SPIRIT 2013 Checklist: Recommended items to address in a clinical trial protocol and related documents*

Section/item	Item No	Description	Addressed on page number
**Administrative information**			
Title	1	Descriptive title identifying the study design, population, interventions, and, if applicable, trial acronym	Title page, p1
Trial registration	2a	Trial identifier and registry name. If not yet registered, name of intended registry	Abstract, p2
	2b	All items from the World Health Organization Trial Registration Data Set	N/A (see registry)
Protocol version	3	Date and version identifier	Footnotes, p19
Funding	4	Sources and types of financial, material, and other support	Footnotes, p19
Roles and responsibilities	5a	Names, affiliations, and roles of protocol contributors	Title page, p1; Footnotes, p19
	5b	Name and contact information for the trial sponsor	Title page, p1
	5c	Role of study sponsor and funders, if any, in study design; collection, management, analysis, and interpretation of data; writing of the report; and the decision to submit the report for publication, including whether they will have ultimate authority over any of these activities	Ethics and Dissemination, p16; Footnotes, p19

For peer review only - http://bmjopen.bmj.com/site/about/guidelines.xhtml

	5d	Composition, roles, and responsibilities of the coordinating centre, steering committee, endpoint adjudication committee, data management team, and other individuals or groups overseeing the trial, if applicable (see Item 21a for data monitoring committee)	N/A
**Introduction**			
Background and rationale	6a	Description of research question and justification for undertaking the trial, including summary of relevant studies (published and unpublished) examining benefits and harms for each intervention	Introduction, p4
	6b	Explanation for choice of comparators	Intervention, p7-8
Objectives	7	Specific objectives or hypotheses	Introduction, p4
Trial design	8	Description of trial design including type of trial (eg, parallel group, crossover, factorial, single group), allocation ratio, and framework (eg, superiority, equivalence, noninferiority, exploratory)	Study design and setting, p6
**Methods: Participants, interventions, and outcomes**			
Study setting	9	Description of study settings (eg, community clinic, academic hospital) and list of countries where data will be collected. Reference to where list of study sites can be obtained	Study design and setting, p6
Eligibility criteria	10	Inclusion and exclusion criteria for participants. If applicable, eligibility criteria for study centres and individuals who will perform the interventions (eg, surgeons, psychotherapists)	Eligibility criteria, p6; Figure 2
Interventions	11a	Interventions for each group with sufficient detail to allow replication, including how and when they will be administered	Intervention, p7-8;
	11b	Criteria for discontinuing or modifying allocated interventions for a given trial participant (eg, drug dose change in response to harms, participant request, or improving/worsening disease)	Concomitant care and trial follow-up, p11
	11c	Strategies to improve adherence to intervention protocols, and any procedures for monitoring adherence (eg, drug tablet return, laboratory tests)	Study outline, p6-7; Intervention, p7-8

For peer review only - http://bmjopen.bmj.com/site/about/guidelines.xhtml

	11d	Relevant concomitant care and interventions that are permitted or prohibited during the trial	Concomitant care and trial follow-up, p11
Outcomes	12	Primary, secondary, and other outcomes, including the specific measurement variable (eg, systolic blood pressure), analysis metric (eg, change from baseline, final value, time to event), method of aggregation (eg, median, proportion), and time point for each outcome. Explanation of the clinical relevance of chosen efficacy and harm outcomes is strongly recommended	Assessments during study, p8; Outcomes, p12
Participant timeline	13	Time schedule of enrolment, interventions (including any run-ins and washouts), assessments, and visits for participants. A schematic diagram is highly recommended (see Figure)	Study outline, p6-7; Figure 3;Table 1
Sample size	14	Estimated number of participants needed to achieve study objectives and how it was determined, including clinical and statistical assumptions supporting any sample size calculations	Sample size, p12
Recruitment	15	Strategies for achieving adequate participant enrolment to reach target sample size	Study outline, p6-7
**Methods: Assignment of interventions (for controlled trials)**			
Allocation:			
Sequence generation	16a	Method of generating the allocation sequence (eg, computer-generated random numbers), and list of any factors for stratification. To reduce predictability of a random sequence, details of any planned restriction (eg, blocking) should be provided in a separate document that is unavailable to those who enrol participants or assign interventions	Allocation, randomisation and blinding, p8
Allocation concealment mechanism	16b	Mechanism of implementing the allocation sequence (eg, central telephone; sequentially numbered, opaque, sealed envelopes), describing any steps to conceal the sequence until interventions are assigned	Allocation, randomisation and blinding, p8
Implementation	16c	Who will generate the allocation sequence, who will enrol participants, and who will assign participants to interventions	Allocation, randomisation and blinding, p8
Blinding (masking)	17a	Who will be blinded after assignment to interventions (eg, trial participants, care providers, outcome assessors, data analysts), and how	Allocation, randomisation and blinding, p8

For peer review only - http://bmjopen.bmj.com/site/about/guidelines.xhtml

	17b	If blinded, circumstances under which unblinding is permissible, and procedure for revealing a participant's allocated intervention during the trial	Allocation, randomisation and blinding, p8

Methods: Data collection, management, and analysis

Data collection methods	18a	Plans for assessment and collection of outcome, baseline, and other trial data, including any related processes to promote data quality (eg, duplicate measurements, training of assessors) and a description of study instruments (eg, questionnaires, laboratory tests) along with their reliability and validity, if known. Reference to where data collection forms can be found, if not in the protocol	Study outline, p6-7; Assessments, p8-11; Outcomes, p12; Data management, p13-14
	18b	Plans to promote participant retention and complete follow-up, including list of any outcome data to be collected for participants who discontinue or deviate from intervention protocols	Concomitant care and trial follow-up, p11; Study outline, p6-7; Intervention, p7-8
Data management	19	Plans for data entry, coding, security, and storage, including any related processes to promote data quality (eg, double data entry; range checks for data values). Reference to where details of data management procedures can be found, if not in the protocol	Data management, p13-14
Statistical methods	20a	Statistical methods for analysing primary and secondary outcomes. Reference to where other details of the statistical analysis plan can be found, if not in the protocol	Planned statistical analyses, p12-13
	20b	Methods for any additional analyses (eg, subgroup and adjusted analyses)	Planned statistical analyses, p12-13
	20c	Definition of analysis population relating to protocol non-adherence (eg, as randomised analysis), and any statistical methods to handle missing data (eg, multiple imputation)	Planned statistical analyses, p12-13

Methods: Monitoring

Data monitoring	21a	Composition of data monitoring committee (DMC); summary of its role and reporting structure; statement of whether it is independent from the sponsor and competing interests; and reference to where further details about its charter can be found, if not in the protocol. Alternatively, an explanation of why a DMC is not needed	N/A (no DMC), Monitoring, p15
	21b	Description of any interim analyses and stopping guidelines, including who will have access to these interim results and make the final decision to terminate the trial	N/A (no interim analyses)
Harms	22	Plans for collecting, assessing, reporting, and managing solicited and spontaneously reported adverse events and other unintended effects of trial interventions or trial conduct	Assessments, p11; Safety, p14
Auditing	23	Frequency and procedures for auditing trial conduct, if any, and whether the process will be independent from investigators and the sponsor	Monitoring, p15

Ethics and dissemination

Research ethics approval	24	Plans for seeking research ethics committee/institutional review board (REC/IRB) approval	Ethics and Dissemination, p16
Protocol amendments	25	Plans for communicating important protocol modifications (eg, changes to eligibility criteria, outcomes, analyses) to relevant parties (eg, investigators, REC/IRBs, trial participants, trial registries, journals, regulators)	Ethics and Dissemination, p16
Consent or assent	26a	Who will obtain informed consent or assent from potential trial participants or authorised surrogates, and how (see Item 32)	Study outline, p6-7
	26b	Additional consent provisions for collection and use of participant data and biological specimens in ancillary studies, if applicable	Study outline, p6-7
Confidentiality	27	How personal information about potential and enrolled participants will be collected, shared, and maintained in order to protect confidentiality before, during, and after the trial	Data management, p13-14
Declaration of interests	28	Financial and other competing interests for principal investigators for the overall trial and each study site	Footnotes, p19

peer review only

Access to data	29	Statement of who will have access to the final trial dataset, and disclosure of contractual agreements that limit such access for investigators	Ethics and Dissemination, p16; Footnotes, p19
Ancillary and post-trial care	30	Provisions, if any, for ancillary and post-trial care, and for compensation to those who suffer harm from trial participation	Ethics and Dissemination, p16
Dissemination policy	31a	Plans for investigators and sponsor to communicate trial results to participants, healthcare professionals, the public, and other relevant groups (eg, via publication, reporting in results databases, or other data sharing arrangements), including any publication restrictions	Concomitant care and trial follow-up, p11; Ethics and Dissemination, p16
	31b	Authorship eligibility guidelines and any intended use of professional writers	Ethics and Dissemination, p16
	31c	Plans, if any, for granting public access to the full protocol, participant-level dataset, and statistical code	Ethics and Dissemination, p16
**Appendices**			
Informed consent materials	32	Model consent form and other related documentation given to participants and authorised surrogates	Supplementary file S2 and S3
Biological specimens	33	Plans for collection, laboratory evaluation, and storage of biological specimens for genetic or molecular analysis in the current trial and for future use in ancillary studies, if applicable	Data management, p13-14; Assessments, p11

*It is strongly recommended that this checklist be read in conjunction with the SPIRIT 2013 Explanation & Elaboration for important clarification on the items. Amendments to the protocol should be tracked and dated. The SPIRIT checklist is copyrighted by the SPIRIT Group under the Creative Commons "Attribution-NonCommercial-NoDerivs 3.0 Unported" license.

For peer review only

The

DEXA-CORT

Trial

For peer review only

For peer review only - http://bmjopen.bmj.com/site/about/guidelines.xhtml

Proefpersoneninformatie voor deelname aan medisch-wetenschappelijk onderzoek

Cortisol om psychische bijwerkingen van dexamethason te voorkomen

Hydrocortison als co-medicatie om nadelige neuropsychiatrische effecten van dexamethason te voorkomen.

Inleiding

Geachte heer/mevrouw,

Wij vragen u om mee te doen aan een medisch-wetenschappelijk onderzoek.
Meedoen is vrijwillig. Om mee te doen is wel uw schriftelijke toestemming nodig. U ontvangt deze brief omdat bij u een hersentumor is gevonden waaraan u binnenkort wordt geopereerd. Voordat u beslist of u wilt meedoen aan dit onderzoek, is het belangrijk om meer te weten over het onderzoek. Lees deze informatie rustig door. Bespreek het met uw partner, vrienden of familie. Heeft u na het lezen van de informatie nog vragen? Dan kunt u terecht bij de onderzoeker of de onafhankelijk deskundige, die aan het eind van deze brief genoemd wordt. Verdere informatie over meedoen aan zo'n onderzoek staat in de bijgevoegde brochure 'Medisch-wetenschappelijk onderzoek'.

1. Algemene informatie

Dit onderzoek is opgezet door het Leids Universitair Medisch Centrum (LUMC) in samenwerking met het Erasmus MC Universitair Medisch Centrum Rotterdam (EMC), het Universitair Medisch Centrum Utrecht (UMCU) en Haaglanden Medisch Centrum (HMC). Voor dit onderzoek zijn 180 proefpersonen nodig. De medisch-ethische toetsingscommissie van het LUMC heeft dit onderzoek goedgekeurd. Algemene informatie over de toetsing van onderzoek vindt u in de brochure 'Medisch-wetenschappelijk onderzoek'.

2. Doel van het onderzoek

Het doel van dit onderzoek is uitzoeken of de psychische (geestelijke) bijwerkingen van het medicijn dexamethason verminderd kunnen worden. Dexamethason wordt standaard gebruikt bij hersenoperaties om zwelling en ontsteking te voorkomen. Echter, het is bekend dat dexamethason psychische bijwerkingen kan veroorzaken, bijvoorbeeld depressie, angst en verstoorde gedachten. Wij denken dat deze bijwerkingen worden veroorzaakt omdat dexamethason de aanmaak van het lichaamseigen hormoon cortisol (= hormoon gemaakt in de bijnier) afremt. Door nu dit lichaamseigen hormoon cortisol (= hydrocortison) in tabletvorm aan de dexamethason toe te voegen, denken wij dat deze bijwerkingen minder vaak zullen optreden.

For peer review only - http://bmjopen.bmj.com/site/about/guidelines.xhtml

3. Achtergrond van het onderzoek

Dexamethason wordt door dokters aan verschillende groepen patiënten voorgeschreven. Ongeveer 1 op de 5 patiënten krijgt last van psychische bijwerkingen of slaapproblemen. Of deze klachten daadwerkelijk optreden en de ernst hiervan hangen af van de dosis en de duur van de behandeling. Omdat deze klachten zeer hinderlijk kunnen zijn en kunnen leiden tot een verminderde kwaliteit van leven, willen wij met deze studie een behandeling testen om dit soort bijwerkingen tegen te gaan. Meer gedetailleerde informatie over het onderzoek kunt u vinden in **bijlage D:** Extra achtergrond informatie.

4. Wat meedoen inhoudt

Als u meedoet, krijgt u rondom de operatie, tijdens het verblijf in het ziekenhuis twee keer daags een extra tablet toegediend naast de normale behandeling. Deze tablet bevat ofwel het lichaamseigen hormoon hydrocortison, dat bij u niet meer aangemaakt wordt, ofwel een niet werkzame stof (placebo). Tijdens de opname en tot 3 maanden erna wordt u op verschillende momenten onderzocht waarbij u vragenlijsten moet invullen die de aanwezigheid van de psychische bijwerkingen en uw algehele functioneren in kaart brengen. De testmomenten voor het onderzoek worden gekoppeld aan de zorg die u al krijgt. De studiemedicatie zult u gedurende uw opname in het ziekenhuis krijgen en misschien thuis, dit zal maximaal 15 dagen zijn, afhankelijk van hoe lang u de dexamethason moet gebruiken. In de weken na uw ontslag uit het ziekenhuis zullen de vragenlijsten aan u voorgelegd worden op de dag dat u voor controle terug verwacht wordt in het ziekenhuis. Daarnaast moet u nog op 2 andere momenten thuis vragenlijsten invullen. Omdat het laatste meetmoment 3 maanden na de operatie is gepland, is de totale duur van de studie 3 maanden. U zult in totaal ongeveer 5 tot 6 uur met het invullen van de vragenlijsten bezig zijn verspreid over 3 maanden.

Geschiktheidsonderzoek

Eerst bepalen we of u kunt meedoen. Dit gebeurt aan de hand van een aantal voorwaarden waaraan u moet voldoen, daarbij zijn onder andere uw leeftijd en het gebruik van andere medicijnen van belang. Wanneer u besluit mee te doen aan de studie, wordt aan u gevraagd of u een toestemmingsformulier wilt tekenen. Na ondertekening wordt uw deelname aan de studie officieel. De onderzoeker zal dan vragen naar uw medische geschiedenis, zoals psychische achtergrond, medicatie en klachten.

Behandeling

We behandelen u met de studiemedicatie even lang als u volgens de standaard zorg het medicijn dexamethason toegediend krijgt. De ene helft van de mensen die deelnemen aan dit onderzoek krijgt het lichaamseigen hormoon hydrocortison, de andere helft krijgt een tablet waarin geen werkzame stof zit (placebo). Loting bepaalt welke behandeling u krijgt. U, de onderzoeker, verpleegkundige en de arts weten niet in welke groep u zit. Als het voor uw gezondheid belangrijk is, kan dit wel worden opgezocht. Algemene informatie hierover vindt u in de brochure ‘Medisch-wetenschappelijk onderzoek’.

For peer review only - http://bmjopen.bmj.com/site/about/guidelines.xhtml

Bezoeken en metingen

Voor deze studie zijn geen extra bezoeken aan het ziekenhuis nodig. De testen die uitgevoerd moeten worden voor het onderzoek, worden gepland op de dagen dat u toch al in het ziekenhuis bent.

Er zal dan het volgende gebeuren:

- Bij het eerste gesprek vragen we naar uw medische geschiedenis (psychisch en medicatie) en vragen naar klachten.
- We laten u op verschillende momenten vragenlijsten invullen over uw slaapkwaliteit, kwaliteit van leven, hoe u zichzelf voelt en uw psychologisch welzijn.
- We meten op verschillende momenten uw psychisch welzijn via een interview met één van de onderzoekers. Ook zullen de verpleegkundigen uw welzijn in de gaten houden.
- We laten u verschillende korte testen voor geheugen, aandacht en taal uitvoeren.
- We laten u een horloge dragen dat de slaapkwaliteit kan meten.

Veel van de vragenlijsten kunt u (digitaal) thuis invullen. In **bijlage C:** Schema onderzoek handelingen staat welke metingen er bij elk van de bezoeken plaatsvinden. En hoeveel tijd dit ongeveer in beslag zal nemen.

Tijdens het onderzoek kan aan u gevraagd worden om bloed af te staan. In dit bloed willen wij kijken naar genetische variatie. Daarmee onderzoeken we of de reactie op medicatie mede bepaald wordt door de samenstelling van uw DNA.

Daarnaast kan tijdens het onderzoek gevraagd worden of er een stukje van het verwijderde tumor- of hersenweefsel bewaard mag blijven voor nader onderzoek. In dit weefsel willen wij de werking van de hydrocortison onderzoeken.

Het buisje bloed en stukje van het verwijderde weefsel wordt eerst opgeslagen in de lokale onderzoeksinstelling en pas onderzocht nadat de studie is afgerond. Onderzoek naar het lichaamsmateriaal wordt in het LUMC uitgevoerd. Indien u niet in het desbetreffende centrum onder behandeling bent geweest, wordt uw lichaamsmateriaal naar het LUMC gebracht, opgeslagen en onderzocht. Meer gedetailleerde informatie over dit deel van het onderzoek kunt u vinden in **bijlage D:** Extra achtergrond informatie. Als u liever niet aan dit deel van het onderzoek wilt meedoen, kunt u dit aangeven in het toestemmingsformulier.

5. Wat wordt er van u verwacht

Om het onderzoek goed te laten verlopen is het belangrijk dat u zich aan de volgende afspraken houdt.

De afspraken zijn dat u:

- de studiemedicatie inneemt volgens de uitleg.
- niet meedoet aan nog een ander medisch-wetenschappelijk onderzoek.
- afspraken voor bezoeken nakomt.

For peer review only - http://bmjopen.bmj.com/site/about/guidelines.xhtml

Het is belangrijk dat u contact opneemt met de onderzoeker:

- voordat u andere geneesmiddelen gaat gebruiken. Ook als dat homeopathische geneesmiddelen, natuurgeneesmiddelen, vitaminen en/of geneesmiddelen van de drogist zijn.
- als u in de maanden na de operatie in een ziekenhuis wordt opgenomen of behandeld.
- als u plotseling gezondheidsklachten krijgt.
- als u niet meer wilt meedoen aan het onderzoek.
- als uw contactgegevens wijzigen.

Partner, familielid of andere naaste die voor u zorgt
Wij willen voor het onderzoek ook uw partner (of familielid of andere naaste) vragen om drie keer een vragenlijst in te vullen over uw psychisch welzijn.

6. Mogelijke bijwerkingen

Wij kunnen van te voren niet voorspellen of u last gaat krijgen van de dexamethason. Wij verwachten geen extra bijwerkingen door de studiemedicatie, omdat de studiemedicatie nagenoeg gelijk is aan de hoeveelheid lichaamseigen cortisol die normaal geproduceerd zou worden. We vullen een tekort aan. Meedoen aan de studie zal daarom naar onze inschatting geen extra risico's met zich meebrengen naast de al bestaande risico's van uw behandeling.

7. Mogelijke voor- en nadelen

Het is belangrijk dat u de mogelijke voor- en nadelen goed afweegt voordat u besluit mee te doen.

Voordelen van deelname:

- De studiemedicatie zou de mogelijke psychische bijwerkingen van dexamethason kunnen verminderen. Vooral gevoelens van angst en onrust zouden minder kunnen optreden, maar zeker is dat niet.
- De studiemedicatie zou uw slaapkwaliteit kunnen verbeteren, maar zeker is dat niet.

Nadelen van deelname:

- Het is belangrijk dat u de medicijnen met regelmaat en volgens de instructies inneemt.
- De testmomenten waarbij vragenlijsten ingevuld moeten worden kosten tijd en kunnen vermoeiend zijn.
- U heeft afspraken waaraan u zich moet houden.
- De vragenlijsten kunnen confronterend zijn, omdat ze vragen naar hoe u zich voelt en hoe het in het dagelijks leven met u gaat.
- Tijdens de testen voor het geheugen, de aandacht en de taal komen uw sterke maar ook uw minder sterke functies aan het licht. Dit kan soms confronterend zijn.

For peer review only - http://bmjopen.bmj.com/site/about/guidelines.xhtml

8. Als u niet wilt meedoen of wilt stoppen met het onderzoek

U beslist zelf of u meedoet aan het onderzoek. Deelname is vrijwillig. Als u niet wilt meedoen, hoeft u verder niets te doen. U hoeft niets te tekenen. U hoeft ook niet te zeggen waarom u niet wilt meedoen. U krijgt gewoon de behandeling die u anders ook zou krijgen. Er verandert niets.

Als u wel meedoet, kunt u zich altijd bedenken en toch stoppen, ook tijdens het onderzoek. U wordt dan weer op de gebruikelijke manier verder behandeld. U hoeft niet te zeggen waarom u stopt. Wel moet u dit direct melden aan de onderzoeker.
De gegevens die tot dat moment zijn verzameld, worden gebruikt voor het onderzoek. Als u wilt, en indien van toepassing, kan verzameld lichaamsmateriaal worden vernietigd. Dit moet u wel zelf aangeven. Wanneer u dit niet meldt, wordt het gebruikt voor het onderzoek.

Als er nieuwe informatie over het onderzoek is die belangrijk voor u is, laat de onderzoeker dit aan u weten. U wordt dan gevraagd of u blijft meedoen.

9. Einde van het onderzoek

Uw deelname aan het onderzoek stopt als

- alle testmomenten voorbij zijn en de vragenlijsten zijn ingeleverd
- u zelf kiest om te stoppen
- de arts het beter voor u vindt om te stoppen
- de overheid of de beoordelende medisch-ethische toetsingscommissie, besluit om het onderzoek te stoppen.

Het hele onderzoek is afgelopen als alle deelnemers klaar zijn.
De medicatie die u gebruikt heeft bij het onderzoek, is niet beschikbaar na afloop van het onderzoek.

Na het verwerken van alle gegevens informeert de onderzoeker u over de belangrijkste uitkomsten van het onderzoek. Dit gebeurt ongeveer 2 tot 3 jaar na uw deelname.
De onderzoeker kan u ook vertellen welke behandeling u heeft gehad/in welke groep u zat. Als u dit niet wilt, dan kunt u dit tegen de onderzoeker zeggen. De onderzoeker mag het u dan niet vertellen.

For peer review only

For peer review only - http://bmjopen.bmj.com/site/about/guidelines.xhtml

10. Gebruik en bewaren van uw gegevens en lichaamsmateriaal

Voor dit onderzoek worden uw persoonsgegevens en, indien van toepassing, lichaamsmateriaal verzameld, gebruikt en bewaard. Het gaat om gegevens zoals uw naam, adres, geboortedatum en om gegevens over uw gezondheid. Het verzamelen, gebruiken en bewaren van uw gegevens en mogelijk uw lichaamsmateriaal is nodig om de vragen die in dit onderzoek worden gesteld te kunnen beantwoorden en de resultaten te kunnen publiceren. Wij vragen voor het gebruik van uw gegevens en mogelijk lichaamsmateriaal uw toestemming.

Vertrouwelijkheid van uw gegevens en mogelijk lichaamsmateriaal

Om uw privacy te beschermen geven we een code aan uw gegevens en, indien van toepassing, uw lichaamsmateriaal. Uw naam en andere gegevens die u direct kunnen identificeren worden daarbij weggelaten. Alleen met de sleutel van de code zijn gegevens tot u te herleiden. De sleutel van de code blijft opgeborgen in de lokale onderzoeksinstelling. Ook in rapporten en publicaties over het onderzoek zijn de gegevens niet tot u te herleiden.

Toegang tot uw gegevens voor controle

Sommige personen kunnen op de onderzoekslocatie toegang krijgen tot al uw gegevens. Ook tot de gegevens zonder code. Dit is nodig om te kunnen controleren of het onderzoek goed en betrouwbaar is uitgevoerd. Personen die ter controle inzage kunnen krijgen in uw gegevens zijn: de commissie die de veiligheid van het onderzoek in de gaten houdt, monitors die voor het LUMC werken en de Inspectie Gezondheidszorg en Jeugd. Zij houden uw gegevens geheim. Wij vragen u voor deze inzage toestemming te geven.

Bewaartermijn gegevens en lichaamsmateriaal

Uw gegevens moeten 15 jaar worden bewaard op de onderzoekslocatie. Indien van toepassing, uw lichaamsmateriaal wordt voor onbepaalde tijd bewaard in de lokale onderzoeksinstelling. Hiermee willen wij na deze studie nog bepalingen doen. Onderzoek naar het lichaamsmateriaal wordt in het LUMC uitgevoerd. Indien u niet in het desbetreffende centrum onder behandeling bent geweest, wordt uw lichaamsmateriaal voor de onderzoeksbepalingen naar het LUMC gebracht, opgeslagen en onderzocht. De gegevens en lichaamsmateriaal die vanuit een deelnemend ziekenhuis naar het LUMC worden gestuurd bevatten alleen de code, en niet uw naam of andere gegevens waarmee u kunt worden geïdentificeerd.

For peer review only - http://bmjopen.bmj.com/site/about/guidelines.xhtml

Intrekken toestemming

U kunt uw toestemming voor gebruik van uw persoonsgegevens en mogelijk lichaamsmateriaal altijd weer intrekken. Dit geldt voor dit onderzoek en ook voor het bewaren en het gebruik voor het toekomstige onderzoek. De onderzoeksgegevens die zijn verzameld tot het moment dat u uw toestemming intrekt worden nog wel gebruikt in het onderzoek. Uw lichaamsmateriaal wordt alleen op uw verzoek vernietigd. Als er al metingen met dat lichaamsmateriaal zijn gedaan, dan worden die gegevens nog wel gebruikt.

Meer informatie over uw rechten bij verwerking van gegevens

Voor algemene informatie over uw rechten bij verwerking van uw persoonsgegevens kunt u de website van de Autoriteit Persoonsgegevens raadplegen.

Bij vragen over uw rechten kunt u contact opnemen met de verantwoordelijke voor de verwerking van uw persoonsgegevens. Voor dit onderzoek is dat: het LUMC. Zie **bijlage A:** Contactgegevens.

Bij vragen of klachten over de verwerking van uw persoonsgegevens raden we u aan eerst contact op te nemen met de onderzoekslocatie. U kunt contact opnemen met de Functionaris voor de Gegevensbescherming van de instelling of de Autoriteit Persoonsgegevens. Zie **bijlage A:** Contactgegevens.

Registratie van het onderzoek

Informatie over dit onderzoek is ook opgenomen in een overzicht van medisch-wetenschappelijke onderzoeken, namelijk in het Nederlands Trial Register (http://www.trialregister.nl). Daarin zijn geen gegevens opgenomen die naar u herleidbaar zijn. Na het onderzoek kan de website een samenvatting van de resultaten van dit onderzoek tonen. U vindt dit onderzoek onder de naam: DEXA-CORT.

11. Verzekering voor proefpersonen

Voor iedereen die meedoet aan dit onderzoek is een verzekering afgesloten. De verzekering dekt schade door het onderzoek. Niet alle schade is gedekt. In **bijlage B:** Informatie over de verzekering, vindt u meer informatie over de verzekering en de uitzonderingen. Daar staat ook aan wie u de schade kunt melden.

12. Informeren huisarts en behandelend specialist

Wij zullen uw huisarts niet informeren dat u meedoet aan het onderzoek. Als u dit liever wel wilt, kunt u altijd zelf contact opnemen met uw huisarts om te vertellen dat u aan de studie meedoet. Bij vragen over uw medische geschiedenis of medicijngebruik, nemen we wel contact op met uw huisarts of behandeld arts. U kunt niet deelnemen aan het onderzoek als u geen huisarts heeft. Uw behandelend arts wordt wel geïnformeerd over uw deelname via uw dossier.

For peer review only - http://bmjopen.bmj.com/site/about/guidelines.xhtml

13. Geen vergoeding voor meedoen

De studiemedicatie voor het onderzoek kost u niets. U wordt niet betaald voor het meedoen aan dit onderzoek. Studie afspraken worden gepland op dagen dat u al een afspraak in het ziekenhuis heeft. Wanneer u voor het onderzoek extra naar het ziekenhuis moet komen, zullen uw reiskosten worden vergoed.

14. Heeft u vragen?

Bij vragen kunt u contact opnemen met de onderzoeker of het onderzoeksteam. Voor onafhankelijk advies over meedoen aan dit onderzoek kunt u terecht bij de onafhankelijke arts; dr. Stijn W. Genders. Hij weet veel over het onderzoek, maar hij is niet betrokken bij deze studie. Indien u klachten heeft over het onderzoek, kunt u dit bespreken met de onderzoeker of uw behandelend arts. Wilt u dit liever niet, dan kunt u zich wenden tot de klachtenfunctionaris van uw ziekenhuis. Alle gegevens vindt u in **bijlage A**: Contactgegevens.

15. Ondertekening toestemmingsformulier

Wanneer u voldoende bedenktijd van minimaal 1 week en maximaal 2 weken heeft gehad, wordt u gevraagd te beslissen over deelname aan dit onderzoek. Indien u toestemming geeft, zullen wij u vragen deze op de bijbehorende toestemmingsverklaring schriftelijk te bevestigen. Door uw schriftelijke toestemming geeft u aan dat u de informatie heeft begrepen en instemt met deelname aan het onderzoek. Zowel uzelf als de onderzoeker ontvangen een getekende versie van deze toestemmingsverklaring.

Dank voor uw aandacht.

For peer review only

For peer review only - http://bmjopen.bmj.com/site/about/guidelines.xhtml

16. Bijlagen bij deze informatie

A. Contactgegevens

B. Informatie over de verzekering

C. Schema onderzoek handelingen

D. Extra achtergrond informatie

E. Toestemmingsformulier

F. Toestemmingsformulier voor partner (of familielid of andere naaste)

G. Brochure ‘Medisch-wetenschappelijk onderzoek. Algemene informatie voor de proefpersoon’

For peer review only

For peer review only - http://bmjopen.bmj.com/site/about/guidelines.xhtml

Bijlage A: Contactgegevens

**Coördinerend onderzoeker**	**Onafhankelijk arts**
Anne-Sophie Koning, MSc	Dr. Stijn W. Genders
dexonderzoek@lumc.nl	oog.stafsecretariaat@lumc.nl
+31-71-5265303 / +31-71-5263082	+31-71-5262374
**LUMC contact gegevens**	
Dr. Wouter R. van Furth	
neurochirurgie@lumc.nl	
+31-71-5262109	
**Functionaris voor de Gegevensbescherming LUMC**	
Wanneer u vragen heeft over de bescherming van uw privacy kunt u contact opnemen de functionarissen gegevensbescherming van het LUMC (FG) via infoavg@lumc.nl	
**Klachtenfunctionaris van het LUMC**	
Bij klachten kunt u zich melden bij het patiëntenservicebureau in het LUMC, locatie H2-11 (routenummer 473, tegenover het Leidseplein). Hier kunt u melding maken van uw onvrede en het klachtenformulier invullen. Het patiëntenservicebureau informeert u zo snel mogelijk over een mogelijke oplossing en kan eventueel de klachtenfunctionaris inschakelen. U kunt ook het klachtenformulier digitaal invullen. Zie website LUMC pagina klacht indienen<br><br>**Contactgegevens patiëntenservicebureau**<br>LUMC Patiëntenservicebureau<br>Postbus 9600<br>2300 RC Leiden<br>Telefoon: +31 71-5262989	

For peer review only - http://bmjopen.bmj.com/site/about/guidelines.xhtml

Bijlage B: Informatie over de verzekering

Voor iedereen die meedoet aan dit onderzoek heeft het LUMC een verzekering afgesloten. De verzekering dekt schade door deelname aan het onderzoek. Dit geldt voor schade tijdens het onderzoek of binnen vier jaar na het einde van uw deelname aan het onderzoek. Schade moet u binnen die vier jaar aan de verzekeraar hebben gemeld.

De verzekering dekt niet alle schade. Onderaan deze tekst staat in het kort welke schade niet wordt gedekt.
Deze bepalingen staan in het Besluit verplichte verzekering bij medisch-wetenschappelijk onderzoek met mensen. Dit besluit staat op www.ccmo.nl, de website van de Centrale Commissie Mensgebonden Onderzoek (zie 'Bibliotheek' en dan 'Wet- en regelgeving').

Bij schade kunt u direct contact leggen met de verzekeraar.

De verzekeraar van het onderzoek is:	
Naam:	Centramed
Adres:	Maria Montessorilaan 9, 2719 DB Zoetermeer
Telefoonnummer:	070-3017070
E-mail:	info@centramed.nl
Polisnummer:	624.530.305

De verzekering biedt een dekking van € 650.000,- per proefpersoon en € 5.000.000,- voor het hele onderzoek (en € 7.500.000,- per jaar voor alle onderzoeken van het Leids Universitair Medisch Centrum).

De verzekering dekt de volgende schade **niet**:

- schade door een risico waarover u in de schriftelijke informatie bent ingelicht. Dit geldt niet als het risico zich ernstiger voordoet dan was voorzien of als het risico heel onwaarschijnlijk was;
- schade aan uw gezondheid die ook zou zijn ontstaan als u niet aan het onderzoek had meegedaan;
- schade door het niet (volledig) opvolgen van aanwijzingen of instructies;
- schade aan uw nakomelingen, als gevolg van een negatief effect van het onderzoek op u of uw nakomelingen;
- schade door een bestaande behandelmethode bij onderzoek naar bestaande behandelmethoden.

For peer review only - http://bmjopen.bmj.com/site/about/guidelines.xhtml

Bijlage C: Schema onderzoek handelingen

Voor deze studie zijn geen extra bezoeken aan het ziekenhuis nodig. De testen die uitgevoerd moeten worden voor het onderzoek, worden gepland op de dagen dat u in het ziekenhuis bent.

Poli bezoek

Volgens reguliere zorg wordt er een poli afspraak gepland met de neurochirurg. Op de dag van deze poli afspraak willen wij u vragen 1 vragenlijst in te vullen, 1 interview af te laten nemen en een aantal testen voor het geheugen, de aandacht en de taal uit te voeren. Indien mogelijk, ook 1 vragenlijst voor uw partner (of familielid of andere naaste). Bij patiënten met een linkszijdige hersentumor willen wij nog een extra taalscreening doen. In totaal zal dit studiemoment ongeveer 80 (of 70) minuten duren.
Verder zullen wij via de email 5 vragenlijsten naar u sturen, die u thuis kunt invullen. Het invullen van alle vragenlijsten zal in totaal ongeveer 45 minuten in beslag nemen.

Dagen op verpleegafdeling

Op de dag van opname en op de dag van de operatie hoeft u niks te doen. Na de operatie zult u een aantal dagen in het ziekenhuis verblijven volgens reguliere zorg. Het aantal dagen dat u in het ziekenhuis ligt is afhankelijk van het oordeel van de artsen. Dit wordt volgens reguliere zorg bepaald. Op de dagen dat u op de verpleegafdeling verblijft, vragen wij u steeds 1 korte vragenlijst in te vullen. Ook vragen wij u een horloge te dragen waarmee we uw activiteit en slaap kwaliteit kunnen meten. Daarnaast willen wij u vragen in een slaapdagboek kort aan te geven hoe u geslapen heeft. De tijd die u deze dagen in het ziekenhuis kwijt bent aan het onderzoek is maximaal 10 minuten. Gedurende uw opname in het ziekenhuis zullen verpleegkundigen u psychisch welzijn in de gaten houden.

Dag van ontslag

Op de dag dat u naar huis mag, willen wij een interview bij u afnemen, vragen wij u 2 vragenlijsten in te vullen en we zullen een korte screening voor het geheugen, de aandacht en de taal uitvoeren. Ook willen wij uw partner (of familielid of andere naaste) vragen 1 vragenlijst in te vullen. Bij patiënten met een linkszijdige hersentumor willen wij nog een extra taalscreening doen. Dit studiemoment zal ongeveer 55 (of 45) minuten duren.

Na operatie

Ongeveer 2 weken na de operatie willen wij u vragen 4 vragenlijsten in te vullen. Dit kunt u gewoon thuis doen en het invullen van alle vragenlijsten zal in totaal ongeveer 30 minuten duren.
Rond 5 tot 8 weken na de operatie, wordt er volgens reguliere zorg een afspraak in het ziekenhuis gepland. Op deze dag in het ziekenhuis willen wij nog 1 keer het interview bij u afnemen, vragen wij u nog 2 vragenlijsten in te vullen en een aantal testen voor het geheugen, de aandacht en de taal uit te voeren. Indien mogelijk, ook 1 vragenlijst voor uw partner (of familielid of andere naaste). Dit studiemoment zal ongeveer 80 minuten duren.

For peer review only - http://bmjopen.bmj.com/site/about/guidelines.xhtml

Ongeveer 3 maanden na operatie willen wij u vragen 5 vragenlijsten in te vullen. Dit kunt u gewoon thuis doen en het invullen van alle vragenlijsten zal in totaal ongeveer 45 minuten duren.

In totaal zal de studie ongeveer 5 tot 6 uur van u tijd kosten verspreid over 3 maanden.

**Studiemoment**	**Waar**	**Hoe lang**
Preoperatief	Poli bezoek	70 (tot 80) minuten
	Thuis	45 minuten
Gedurende ziekenhuisopname	Op de afdeling	30 minuten
Dag van ontslag	Op de afdeling	45 (tot 55) minuten
2 weken postoperatief	Thuis	30 minuten
5 – 8 weken postoperatief	Ziekenhuis	80 minuten
3 maanden postoperatief	Thuis	45 minuten
**Totaal**		**5 – 6 uur**

For peer review only - http://bmjopen.bmj.com/site/about/guidelines.xhtml

Bijlage D – Extra achtergrond informatie

Ons lichaam maakt het hormoon cortisol. Cortisol beïnvloedt veel dingen, zoals onze stemming en onze slaap. Cortisol wordt gemaakt in de bijnieren, die aangezet worden door een ander hormoon adrenocorticotroop hormoon (ACTH). Dat ACTH komt uit de hypofyse, een orgaan onder de hersenen. Als er genoeg cortisol is, krijgt de hypofyse een seintje om te stoppen met het maken van hormonen, waardoor de bijnieren dan ook geen cortisol meer maken.

Het medicijn dexamethason lijkt heel erg veel op cortisol dat het lichaam zelf kan maken. Maar het is veel sterker dan het lichaamseigen cortisol. Een kleine dosis dexamethason kan er al voor zorgen dat de hypofyse geen hormoon meer maakt. Daardoor stoppen de bijnieren met cortisol maken. Dus een bijwerking bij behandeling met dexamethason is dat ons eigen lichaam geen cortisol meer maakt.

Er is één groot verschil tussen cortisol en dexamethason. Dexamethason werkt via één aangrijpingspunt. Dat is via de glucocorticoïd receptor (GR). Cortisol werkt op twéé aangrijpingspunten: via de GR, maar ook via de mineralocorticoïd receptor (MR). Cortisol activeert dus MR en GR. Dexamethason activeert alleen de GR. Omdat ons lichaam na dexamethason geen cortisol meer maakt, kan cortisol ook niet meer de MR activeren. Het resultaat is dat alleen de GR actief is, terwijl de MR leeg blijft. Wij denken dat de bijwerkingen na dexamethason komen doordat de MR leeg is, omdat de werking van cortisol in de hersenen van de MR afhangt.

In deze studie voegen we cortisol toe aan de behandeling met dexamethason (als pilletje heeft cortisol de naam 'hydrocortison' gekregen). De hydrocortison kan binden aan de MR. Op deze manier hopen wij de normale werking van de MR weer terug te krijgen. En hopen we dat de bijwerkingen zullen verminderen.

Tijdens het onderzoek kan gevraagd worden of er bloed van u mag worden afgenomen en bewaard mag worden voor verder onderzoek. In het bloed willen wij kijken welk type van MR u heeft. Dit kunnen wij onderzoeken door in het DNA naar het gen van de MR te kijken. Het is namelijk bekend dat een bepaalde variant van het MR gen een lager risico geeft op het krijgen van depressie. Wij willen in dit onderzoek kijken of dit een onderliggende reden kan zijn voor de klachten die sommige patiënten van de dexamethason krijgen.

Met het weefsel dat van het verwijderde tumorweefsel gehaald wordt, willen wij de werking van de hydrocortison onderzoeken. Met de toegevoegde hydrocortison verwachten wij dat de MR weer actief wordt. Dat willen wij in dit weefsel meten.

For peer review only

For peer review only - http://bmjopen.bmj.com/site/about/guidelines.xhtml

Bijlage E: Toestemmingsformulier proefpersoon

Cortisol om psychische bijwerkingen van dexamethason te voorkomen

- Ik heb de informatiebrief gelezen. Ook kon ik vragen stellen. Mijn vragen zijn voldoende beantwoord. Ik had genoeg tijd om te beslissen of ik meedoe.

- Ik weet dat meedoen vrijwillig is. Ook weet ik dat ik op ieder moment kan beslissen om toch niet mee te doen of te stoppen met het onderzoek. Daarvoor hoef ik geen reden te geven.

- Ik geef toestemming voor het opvragen van informatie bij mijn huisarts/specialist(en) die mij behandelt indien er vragen zijn over uw medische geschiedenis of medicijngebruik.

- Ik weet dat voor de controle van het onderzoek sommige mensen toegang tot al mijn gegevens kunnen krijgen. Die mensen staan vermeld in deze informatiebrief. Ik geef toestemming voor die inzage door deze personen.

- Ik weet dat mijn partner (of familielid of andere naaste) ook een aantal keer een vragenlijst moet invullen en hij/zij gaat hiermee akkoord.

- Ik geef toestemming voor het verzamelen en gebruiken van mijn gegevens voor de beantwoording van de onderzoeksvraag in dit onderzoek.

- Ik geef □ **wel**
  □ **geen**
  toestemming voor bloedafname en dit te bewaren en te gebruiken voor toekomstig onderzoek op het gebied van onderzoek beschreven in de informatiebrief.

- Ik geef □ **wel**
  □ **geen**
  toestemming voor het bewaren van een stukje weefsel van het verwijderde tumorweefsel en dit te bewaren en te gebruiken voor toekomstig onderzoek op het gebied van onderzoek beschreven in de informatiebrief.

For peer review only

For peer review only - http://bmjopen.bmj.com/site/about/guidelines.xhtml

Versie 4, 14-02-20

- Ik geef □ **wel**
  □ **geen**
  toestemming om mijn persoonsgegevens en lichaamsmateriaal langer te bewaren en te gebruiken voor toekomstig onderzoek op het gebied van onderzoek beschreven in de informatiebrief.

- Ik wil □ **wel**
  □ **niet**
  geïnformeerd worden over welke behandeling ik heb gehad/in welke groep ik zat.

- Ik wil meedoen aan dit onderzoek.

Naam proefpersoon: ___________________

Handtekening:________________________ Datum : __ / __ / __

Ik verklaar dat ik deze proefpersoon volledig heb geïnformeerd over het genoemde onderzoek.

Als er tijdens het onderzoek informatie bekend wordt die de toestemming van de proefpersoon zou kunnen beïnvloeden, dan breng ik hem/haar daarvan tijdig op de hoogte.

Naam onderzoeker (of diens vertegenwoordiger):________________________________

Handtekening:_______________________ Datum: __ / __ / __

For peer review only - http://bmjopen.bmj.com/site/about/guidelines.xhtml

Bijlage F: Toestemmingsformulier voor partner (of familielid of andere naaste)

Cortisol om psychische bijwerkingen van dexamethason te voorkomen

- Ik ben op de hoogte dat mijn partner (of familielid of andere naaste) meedoet met het onderzoek. Ook kon ik vragen stellen. Mijn vragen zijn voldoende beantwoord. Ik had genoeg tijd om te beslissen of ik voor de studie 3x een vragenlijst wil invullen over mijn partner (of familielid of andere naaste).

- Ik weet dat meedoen vrijwillig is. Ook weet ik dat ik op ieder moment kan beslissen om toch niet mee te doen of te stoppen met het onderzoek. Daarvoor hoef ik geen reden te geven.

- Ik wil meedoen aan dit onderzoek.

Naam partner (of familielid of andere naaste):______________

Handtekening:_______________________ Datum : __ / __ / __

Ik verklaar dat ik de partner (of familielid of andere naaste) volledig heb geïnformeerd over het genoemde onderzoek.

Als er tijdens het onderzoek informatie bekend wordt die de toestemming van de partner (of familielid of andere naaste) zou kunnen beïnvloeden, dan breng ik hem/haar daarvan tijdig op de hoogte.

Naam onderzoeker (of diens vertegenwoordiger):______________________________

Handtekening:______________________ Datum: __ / __ / __

For peer review only - http://bmjopen.bmj.com/site/about/guidelines.xhtml

Version 4, 14-02-20

The

DEXA-CORT

Trial

For peer review only

For peer review only - http://bmjopen.bmj.com/site/about/guidelines.xhtml

Study information for participation in medical research

Cortisol to prevent psychiatric adverse effects of dexamethasone

Hydrocortisone as co-medication to prevent adverse neuropsychiatric effects of dexamethasone

Introduction

Dear Sir/Madam,

You are kindly requested to take part in a medical scientific study. We have contacted you because you will undergo surgery for a brain tumor. Participation is voluntarily and requires your written consent. Before you decide whether you want to participate in this study, you will be given an explanation about what the study involves. Please read this information carefully and discuss it with your partner, friends and/or family. If you have any questions you can contact the (coordinating) investigator or independent expert for an explanation. General information about participating in a study can be found in the enclosed general brochure on medical research.

1. General information

This research was set up by the Leiden University Medical Center (LUMC) in collaboration with the Erasmus MC University Medical Center Rotterdam (EMC), the University Medical Center Utrecht (UMCU) and Haaglanden Medical Center (HMC). 180 participants are needed for this study. The medical ethics review committee of the LUMC has approved this research. General information about the assessment of research can be found in the brochure 'Medical scientific research'.

2. Purpose of the study

The aim of this study is to find out whether the psychological (mental) side effects of the drug dexamethasone can be reduced. Dexamethasone is commonly used in brain surgery to prevent swelling and inflammation. However, it is known that dexamethasone can cause psychological side effects, for example depression, anxiety and disturbed thoughts. We think that these side effects are caused because dexamethasone inhibits the production of the body's own hormone cortisol (= hormone made in the adrenal gland). By now adding this body's hormone cortisol (= hydrocortisone) in tablet form to the dexamethasone, we think that these side effects will occur less often.

3. Background of the study

Dexamethasone is prescribed by doctors to different groups of patients. About 1 in 5 patients suffer from psychological side effects or sleeping problems. Whether these complaints actually

For peer review only - http://bmjopen.bmj.com/site/about/guidelines.xhtml

occur and their severity may depend on the dose and the duration of the treatment. Because these complaints can be very bothersome and can lead to a reduced quality of life, we want to use this study to test a treatment to counteract these kinds of side effects. More detailed information about the study can be found in **Appendix** D: Additional background information.

4. What does participation involve?

If you participate, you will be given an extra tablet twice a day in addition to the normal treatment during the hospital stay. This tablet contains either the body's own hormone hydrocortisone, which you no longer produce yourself, or an inactive substance (placebo). During the admission and up to 3 months afterwards, you will be examined at various times during which you have to fill in questionnaires that map the presence of the psychological side effects and your overall functioning. The test moments for the study are coinciding with standard appointments at the hospital. You will receive the study medication during your hospital stay and perhaps at home, this will be up to 15 days, depending on how long you need to take the dexamethasone. In the weeks following your discharge from hospital, the questionnaires will be presented to you on the day you are expected to return to the hospital for a check-up. In addition, you have to complete questionnaires at home at 2 other times. Because the last measurement moment is planned 3 months after surgery, the total duration of the study is 3 months. You will spend a total of about 5 to 6 hours filling in the questionnaires spread over 3 months.

Screening

If you are interested in participating we first will evaluate whether you may participate. This is determined on the basis of a number of conditions that you must meet, including your age and the use of other medicines. When you decide to participate in the study, you will be asked to sign a consent form. After signing, your participation in the study becomes official. The researcher will then ask about your medical history, such as psychological background, medication and complaints.

Treatment

We will treat you with the study medication for as long as you are administered the drug dexamethasone according to standard care. Half of the people participating in this study will be given the body's own hormone hydrocortisone, the other half will be given a tablet that does not contain an active ingredient (placebo). The treatment you will receive is determined by draw. You, the researcher, nurse and doctor do not know which group you are in. If it is important for your health, this can be looked up. General information about this can be found in the brochure 'Medical-scientific research'.

Visits and measurements

No additional hospital visits are required for this study. The tests to be performed for the examination are scheduled on the days that you are already in the hospital.

The following will then happen:

For peer review only - http://bmjopen.bmj.com/site/about/guidelines.xhtml

- At the first meeting we ask about your medical history (psychological and medication) and complaints.
- We let you fill in questionnaires at various times about your sleep quality, quality of life, how you feel and your psychological well-being.
- We measure your psychological well-being at various times through an interview with one of the researchers. The nurses will also monitor your well-being.
- We let you perform several short tests for memory, attention and language.
- We let you wear a watch that can measure sleep quality.

You can complete many of the questionnaires (digitally) at home. **Appendix C**: Schedule of investigation actions, lists the measurements that take place during each of the visits. And how long this will approximately take.

During the study, you may be asked to donate a tube of blood. In this blood sample we want to look at genetic variation. We use this to investigate whether the response to medication is partly determined by the composition of your DNA.

In addition, you may be asked whether a piece of the removed tumor tissue may be kept for further examination. We want to investigate the effect of the hydrocortisone in this tissue.

The samples of blood and/or tissue are first stored at the local study facility and examined only after the study is completed. Research on the body material is carried out in the LUMC. If you have not been treated in this center, your body material will be taken to the LUMC, stored and examined. More detailed information about this part of the study can be found in **Appendix D**: Additional background information. If you prefer not to participate in this part of the study, you can indicate this in the consent form.

5. What is expected of you?

In order for the study to run smoothly, it is important that you adhere to the following agreements.

The agreements are that you:

- Take the study medication according to the instructions.
- Do not participate in any other medical scientific research.
- Fulfill appointments for study visits.

It is important that you contact the researcher:

- before taking any other medicines. Even if they are homeopathic medicines, natural medicines, vitamins and/or medicines from the drugstore.
- if you are hospitalized or treated in the months following the operation.
- if you suddenly develop health problems.
- if you no longer wish to participate in the study.
- if your contact details change.

For peer review only - http://bmjopen.bmj.com/site/about/guidelines.xhtml

Partner, family member or other close relative

Before the study, we would also like to ask your partner (or family member or other close relative) to complete a questionnaire about your psychiatric well-being three times.

6. Possible side effects

We cannot predict in advance whether you will suffer from the dexamethasone. We do not expect any additional side effects from the study medication, because the study medication is almost equal to the amount of the body's own cortisol that would normally be produced. We refill a shortage. In our opinion, participating in the study will therefore not entail any additional risks in addition to the already existing risks of your treatment.

7. Possible advantages and disadvantages

It is important that you carefully consider the possible advantages and disadvantages before you decide to participate.

Benefits of participating:

- The study medication could reduce the possible psychiatric side effects of dexamethasone. Especially feelings of fear and restlessness could occur less, but this is not certain.
- The study medication could improve your sleep quality, but this is not certain.

Disadvantages of participating:

- It is important that you take the medicines regularly and according to the instructions.
- The test moments where questionnaires have to be completed take time and can be tiring.
- You have appointment that you must fulfill.
- The questionnaires can be confronting, because they ask about how you feel and how you are doing in daily life.
- Memory, attention and language tests will reveal your strengths and weaknesses. This can sometimes be confronting.

8. If you do not wish to participate or wish to stop participating

It is up to you to decide whether or not to participate in the study. Participation is voluntary. If you do not want to participate, you will continue your usual treatment as before. You do not have to sign anything and you do not have to explain why you do not want to participate.

If you do participate in the study, you can always change your mind. You may stop participation at any time during the study. This is not harmful and will not affect your usual treatment. You do not have to say why you are stopping, but you do need to tell the investigator immediately. The data collected until that time can still be used for the study.

For peer review only - http://bmjopen.bmj.com/site/about/guidelines.xhtml

If you wish, and if applicable, collected body material can be destroyed. You must indicate this yourself. If you do not report this, it will be used for the investigation.

If there is new information about the study that is important to you, the researcher will let you know. You will then be asked if you want to continue participating.

9. End of the study

Your participation in the study will end if:

- all test moments are done and the questionnaires have been submitted
- you choose to stop yourself
- the doctor thinks it's better for you to stop
- the government or the assessing medical-ethical review committee, decides to stop the research.

The entire study ends when all participants completed the study.
The medication you took during the study will not be available after the study.

After processing all the data, the researcher will inform you about the most important results of the study. This happens approximately 2 to 3 years after your participation.
The researcher can also tell you what treatment you received. If you do not want this, you can say so to the researcher. The researcher is not allowed to tell you.

10. Use and storage of your data and body material

For this research, your personal data and, if applicable, body material will be collected, used and stored. This concerns data such as your name, address, date of birth and data about your health. The collection, use and storage of your data and possibly your body material is necessary to answer the questions posed in this study and to publish the results. We ask your permission for the use of your data and possible body material.

Confidentiality of your data and possible body material

To protect your privacy, we give a code to your data and, if applicable, your body material. Your name and other information that can directly identify you are omitted. Data can only be traced back to you with the key of the code. The key to the code remains stored in the local research facility. Also in reports and publications about the research, the data cannot be traced back to you.

Access your data

Some individuals may have access to all of your data at the study site. Also to the data without code. This is necessary to be able to check whether the research has been carried out properly and reliably. Persons who can access your data for inspection are: the committee that monitors

For peer review only - http://bmjopen.bmj.com/site/about/guidelines.xhtml

the safety of the research, a monitor who works for the LUMC and the Health and Youth Care Inspectorate. They keep your details secret. We ask you to give permission for this inspection.

Storing period of the data and body material

Your data must be kept at the study location for 15 years. If applicable, your body material will be stored indefinitely at the local research facility. We intend further research with these materials after this study. Research into the body material is carried out in the LUMC. If you have not been treated in the center in question, your body material will be brought to the LUMC for the examination, storage and examination. The data and body material sent to the LUMC from a participating hospital only contain the code, and not your name or other data with which you can be identified.

Withdraw permission

You can always withdraw your consent for the use of your personal data and possible body material. This applies to this research and also to the storage and use for future research. The research data collected up to the moment you withdraw your consent will still be used in the research. Your body material will only be destroyed at your request. If measurements have already been taken with the body material, those data will still be used.

More information about your rights when processing data

For general information about your rights when processing your personal data, you can consult the website of the Dutch Data Protection Authority.

If you have any questions about your rights, please contact the person responsible for processing your personal data. For this study, that is: the LUMC. See **Appendix A**: Contact Details.

If you have any questions or complaints about the processing of your personal data, we recommend that you first contact the research location. You can contact the Data Protection Officer of the institution or the Dutch Data Protection Authority. See **Appendix A**: Contact Details.

Registration of the study

Information about this study is also included in an overview of medical scientific studies, namely in the Dutch Trial Register (http://www.trialregister.nl). It does not contain any data that can be traced back to you. After finishing the study, the website may display a summary of the results of this survey. You will find this study under the name: DEXA-CORT.

For peer review only - http://bmjopen.bmj.com/site/about/guidelines.xhtml

11. Insurance for study participants

Insurance has been taken out for everyone participating in this study. The insurance covers damage caused by the study. Not all damage is covered. In **Appendix B**: Information about the insurance, you will find more information about the insurance and the exceptions. It also states who you can report the damage to.

12. Informing the general practitioner and treating specialist

We will not inform your GP that you are participating in the study. If you prefer, you can always contact your GP yourself to let them know that you are participating in the study. If we have any questions about your medical history or medication use, we will contact your GP or treating physician. You cannot participate in the study if you do not have a GP. Your treating physician will be informed about your participation.

13. No compensation for participating

The study medication for the study are free of charge for you. You will not be paid for participating in this study. Study appointments are scheduled on days when you already have a hospital appointment. If we cannot schedule coincide appointments and you have to visit the hospital only for study appointments, you will be reimbursed for your travel costs.

14. Any questions?

If you have any questions, please contact the researcher or the research team. For independent advice about participating in this study, you can contact the independent doctor; Dr. Stijn W. Genders. He knows a lot about the research, but he is not involved in this study. If you have any complaints about the study, you can discuss this with the researcher or your attending physician. If you prefer not to do this, you can contact the complaints officer of your hospital. All details can be found in **Appendix A**: Contact details.

15. Signing the consent

You do not have to decide right away whether you want to participate in the study. When you have had sufficient reflection time of at least 1 week and a maximum of 2 weeks, you will be asked to decide whether to participate in this study. If you give permission, we will ask you to confirm this in writing on the accompanying statement of consent. By your written consent, you indicate that you have understood the information and agree to participate in the study. Both you and the researcher will receive a signed version of this consent form.

Thank you for your attention.

For peer review only - http://bmjopen.bmj.com/site/about/guidelines.xhtml

16. Appendices

A. Contact details

B. Insurance information

C. Schedule of investigation actions

D. Additional background information

E. Informed Consent Form

F. Informed Consent Form (for family or other relative)

G. Brochure ‘Medical research. General information for research participants’

For peer review only

For peer review only - http://bmjopen.bmj.com/site/about/guidelines.xhtml

Appendix A: Contact details

**Coordinating investigator**	**Independent expert**
Anne-Sophie Koning, MSc	Dr. S.W. Genders
dexonderzoek@lumc.nl	oog.stafsecretariaat@lumc.nl
+31-71-5265303 / +31-71-5263082	+31-71-5262374
**LUMC contact details**	
Dr. W.R. van Furth	
neurochirurgie@lumc.nl	
+31-71-5262109	
**Data protection officers of the LUMC**	
If you have any questions about the protection of your privacy, you can contact the data protection officers of the LUMC (FG) via infoavg@lumc.nl	
**Complaints officer of the LUMC**	
In case of complaints, you can report to the patient service desk in the LUMC, location H2-11 (route number 473, opposite Leidseplein). Here you can report your dissatisfaction and fill in the complaint form. The patient service bureau will inform you as soon as possible about a possible solution and may call in the complaints officer if necessary. You can also fill in the complaint form digitally. See website LUMC page submit a complaint.<br><br>**Contact details patient service desk**<br>LUMC Patient service desk<br>Postbus 9600<br>2300 RC Leiden<br>Phone: +31 71 5262989	

For peer review only - http://bmjopen.bmj.com/site/about/guidelines.xhtml

Appendix B: Insurance information

The LUMC has taken out insurance for everyone who participates in this study. The insurance covers damage caused by participating in the study. This applies to damage during the study or within four years of the end of your participation in the study. You must have reported damage to the insurer within those four years.

The insurance does not cover all damage. At the bottom of this text is a brief description of which damage is not covered.
These provisions are contained in the Decree on compulsory insurance for medical research involving humans. This decision can be found on www.ccmo.nl, the website of the Central Committee for Research on Human Subjects (see 'Library' and then 'Laws and regulations').

In the event of damage, you can contact the insurer directly.

The insurance company is:	
Name:	Onderlinge Waarborgmaatschappij Centramed B.A.
Address:	Maria Montessorilaan 9, 2719 DB Zoetermeer
Phone:	070-3017070
Email:	info@centramed.nl
Policy number:	624.530.305

The insurance offers cover of € 650,000 per test participant and € 5,000,000 for the entire study (and € 7,500,000 per year for all studies of the Leiden University Medical Center).

The insurance does **not** cover the following damage::

– damage from a risk of which you have been informed in the written information. This does not apply if the risk is more serious than anticipated or if the risk was very unlikely;
– damage to your health that would also have occurred if you had not taken part in the study;
– damage caused by not (fully) following directions or instructions;
– damage to your descendants, as a result of a negative effect of the research on you or your descendants;
– damage caused by an existing treatment method when researching existing treatment methods.

For peer review only - http://bmjopen.bmj.com/site/about/guidelines.xhtml

Appendix C: Schedule of investigation actions

No additional hospital visits are required for this study. The examinations to be performed are scheduled on the days you are in the hospital.

Outpatient clinic visit

According to regular care, an outpatient clinic appointment is scheduled with the neurosurgeon. On the day of this outpatient clinic appointment, we would like to ask you to fill in 1 questionnaire, to have 1 interview and to perform a number of tests for memory, attention and language. If possible, also 1 questionnaire for your partner (or family member or other relative). We want to do an extra language screening for patients with a left-sided brain tumor. In total, this study moment will last approximately 80 (or 70) minutes.

We will also send you 5 questionnaires via email, which you can complete at home. It will take approximately 45 minutes to complete all questionnaires.

Days on the ward

You do not need to do anything on the day of admission and on the day of surgery. After the surgery you will stay in the hospital for a few days according to regular care. The number of days you will be in the hospital depends on the judgment of the doctors. This is determined according to regular care. On the days that you stay on the nursing ward, we always ask you to fill in 1 short questionnaire. We also ask you to wear a watch with which we can measure your activity and sleep quality. In addition, we would like to ask you to briefly indicate in a sleep diary how you slept. The time you spend in the hospital on the examination these days is a maximum of 10 minutes. During your hospital stay, nurses will monitor your psychiatric well-being.

Day of discharge

On the day you are allowed to go home, we would like to conduct an interview with you, we will ask you to complete 2 questionnaires and we will perform a short screening for memory, attention and language. We would also like to ask your partner (or family member or other close relative) to complete 1 questionnaire. A language screening is conducted for patients with a left-sided brain tumor (mostly according to regular care). This study moment will last approximately 55 (or 45) minutes.

After the surgery

About 2 weeks after the surgery, we would like to ask you to fill in 4 questionnaires. You can do this at home and completing all questionnaires will take approximately 30 minutes in total. According to regular care, an appointment in the hospital is scheduled around 5 to 8 weeks after surgery. On this day in the hospital, we would like to conduct the interview one more time, we ask you to fill in 2 more questionnaires and to perform a number of tests for memory, attention and language. If possible, also 1 questionnaire for your partner (or family member or other close relative). This study moment will last approximately 80 minutes.

For peer review only - http://bmjopen.bmj.com/site/about/guidelines.xhtml

About 3 months after surgery, we would like to ask you to fill in 5 questionnaires. You can do this at home and completing all the questionnaires will take approximately 45 minutes in total.

In total, the study will take about 5 to 6 hours of your time spread over 3 months.

Study moment	Where	How long
Preoperative	Outpatient clinic	70 (tot 80) minutes
	Home	45 minutes
During admission	Ward	30 minutes
Day of discharge	Ward	45 (tot 55) minutes
2 weeks postoperative	Home	30 minutes
5 – 8 weeks postoperative	Outpatient clinic	80 minutes
3 months postoperative	Home	45 minutes
**Total**		**5 – 6 hr**

For peer review only

For peer review only - http://bmjopen.bmj.com/site/about/guidelines.xhtml

Appendix D – Additional background information

Our body produces the hormone cortisol. Cortisol affects many things, such as our mood and our sleep. Cortisol is produced in the adrenal glands, which are regulated by another hormone called adrenocorticotropic hormone (ACTH). The ACTH is made in the pituitary gland, an organ under the brain. When there is enough cortisol, the pituitary gland receives a signal to stop making hormones, this way the adrenal glands no longer make cortisol.

The drug dexamethasone is very similar to cortisol that the body can make itself. But it is much stronger than the body's own cortisol. A small dose of dexamethasone can already ensure that the pituitary gland no longer makes a hormone. This causes the adrenal glands to stop making cortisol. So a side effect of treatment with dexamethasone is that our own bodies no longer make cortisol.

There is one big difference between cortisol and dexamethasone. Dexamethasone works through a single target. This is through the glucocorticoid receptor (GR). Cortisol acts on two targets: via the GR, but also via the mineralocorticoid receptor (MR). Cortisol thus activates MR and GR. Dexamethasone only activates the GR. Because our body no longer makes cortisol after dexamethasone, cortisol can no longer activate the MR. The result is that only the GR is active, while the MR remains empty. We think that the side effects after dexamethasone are because the MR is empty, because the functioning of cortisol in the brain depends on the MR.

In this study we add cortisol to the treatment with dexamethasone (as a pill, cortisol has been given the name 'hydrocortisone'). The hydrocortisone can bind to the MR. In this way we hope to get the normal functioning of the MR again. And we hope that the side effects will decrease.

During the study, you may be asked whether blood may be taken from you and stored for further examination. We want to check in the blood which type of MR you have. We can investigate this by looking at the gene of the MR in the DNA. It is known that a certain variant of the MR gene gives a lower risk of getting depression. In this study we want to see whether this can be an underlying reason for the complaints that some patients get from the dexamethasone.

We want to investigate the effect of the hydrocortisone within the sample tissue that is removed from the removed tumor tissue. We expect the MR to become active again with the added hydrocortisone. We want to measure that in this tissue.

For peer review only

For peer review only - http://bmjopen.bmj.com/site/about/guidelines.xhtml

Appendix E: Informed Consent form participant

Cortisol to prevent psychiatric adverse effects of dexamethasone

- I have read the study information letter. I was also able to ask questions. My questions have been answered to my satisfaction. I had enough time to decide whether to participate.

- I know that participation is voluntary. I know that I may decide at any time not to participate after all or to withdraw from the study. I do not need to give a reason for this.

- I give permission to request information from my general practitioner if there are any questions about your medical history or medication use.

- I know that for the purpose of auditing and monitoring the study, some people may have access to all my data. These people are mentioned in this information letter. I give permission for that access by these persons.

- I know that my partner (or family member or other relative) also has to fill in a questionnaire several times and he/she agrees to this.

- I give permission for the collection and use of my data to answer the research question in this study.

- I
  □ **do**
  □ **do not**
  consent for blood collection and to keep and use this for future research in the field of research described in the information letter.

- I
  □ **do**
  □ **do not**
  consent to keep a sample of the removed tumor tissue and to keep it and use it for future research in the field of research described in the information letter.

- I
  □ **do**
  □ **do not**
  consent to keep my personal data and body material longer and use it for future research in the field of research described in the information letter.

For peer review only

For peer review only - http://bmjopen.bmj.com/site/about/guidelines.xhtml

Version 4, 14-02-20

- I □ **do**
  □ **do not**
  want to be informed on the treatment I received (placebo or hydrocortisone)

- I want to participate in this study.

Name study participant: ___________________

Signature:________________________ Date : __ / __ / __

I hereby declare that I have fully informed this study participant about this study.

If information comes to light during the course of the study that could affect the study participant's consent, I will inform him/her of this in a timely fashion.

Name investigator (or its representative):_______________________________

Signature:_______________________ Date: __ / __ / __

For peer review only

For peer review only - http://bmjopen.bmj.com/site/about/guidelines.xhtml

Appendix F: Informed Consent form partner/other relative

Cortisol to prevent psychiatric adverse effects of dexamethasone

- I am aware that my partner (or family member or other close relative) is participating in the study. I was also able to ask questions. My questions have been answered to my satisfaction. I have had enough time to decide whether I want to fill in a questionnaire about my partner (or relative or other close relative) 3 times for the study.

- I know that participation is voluntary. I know that I may decide at any time not to participate after all or to withdraw from the study. I do not need to give a reason for this.

- I want to participate in this study.

Name partner (or family member or other relative):______________

Signature:_______________________ Date : __ / __ / __

I hereby declare that I have fully informed this partner (or other close relative) about this study.

If information comes to light during the course of the study that could affect the partner's (or other close relative's) consent, I will inform him/her of this in a timely fashion.

Name investigator (or its representative):______________________________

Signature:______________________ Date: __ / __ / __

For peer review only - http://bmjopen.bmj.com/site/about/guidelines.xhtml

The DEXA-CORT trial: study protocol of a randomized placebo-controlled trial of hydrocortisone in brain tumour patients on the prevention of neuropsychiatric adverse effects caused by perioperative dexamethasone

Journal:	*BMJ Open*
Manuscript ID	bmjopen-2021-054405.R2
Article Type:	Protocol
Date Submitted by the Author:	08-Nov-2021
Complete List of Authors:	Koning, Anne-Sophie; Leiden University Medical Center, Department of Medicine, Division of Endocrinology<br>Satoer, Djaina; Erasmus Medical Center, Department of Neurosurgery<br>Vinkers, C; Amsterdam UMC Locatie VUmc, Department of Psychiatry and Department of Anatomy and Neurosciences; GGZ inGeest, Academic Working Place Depression<br>Zamanipoor Najafabadi, Amir H.; University Neurosurgical Center Holland, Leiden University Medical Center, Haaglanden Medical Center and Haga Teaching Hospitals<br>Biermasz, Nienke; Leiden University Medical Center, Department of Medicine, Division of Endocrinology, and Centre for Endocrine Tumors Leiden (CETL)<br>Nandoe Tewarie, Rishi D.S.; University Neurosurgical Center Holland, Leiden University Medical Center, Haaglanden Medical Center and Haga Teaching Hospitals<br>Moojen, Wouter A.; University Neurosurgical Center Holland, Leiden University Medical Center, Haaglanden Medical Center and Haga Teaching Hospitals<br>van Rossum, Elisabeth; Erasmus Medical Center, Department of Internal Medicine, Division of Endocrinology<br>Dirven, Clemens; Erasmus Medical Center, Department of Neurosurgery<br>Pereira, Alberto; Leiden University Medical Center, Department of Medicine, Division of Endocrinology<br>van Furth, W.R.; University Neurosurgical Center Holland, Leiden University Medical Center, Haaglanden Medical Center and Haga Teaching Hospitals, Department of neurosurgery<br>Meijer, Onno C.; Leiden University Medical Center, Department of Medicine, Division of Endocrinology
**Primary Subject Heading**:	Neurology
Secondary Subject Heading:	Diabetes and endocrinology
Keywords:	NEUROSURGERY, PSYCHIATRY, Clinical trials < THERAPEUTICS, CLINICAL PHARMACOLOGY, General endocrinology < DIABETES & ENDOCRINOLOGY

For peer review only - http://bmjopen.bmj.com/site/about/guidelines.xhtml

SCHOLARONE™
Manuscripts

I, the Submitting Author has the right to grant and does grant on behalf of all authors of the Work (as defined in the below author licence), an exclusive licence and/or a non-exclusive licence for contributions from authors who are: i) UK Crown employees; ii) where BMJ has agreed a CC-BY licence shall apply, and/or iii) in accordance with the terms applicable for US Federal Government officers or employees acting as part of their official duties; on a worldwide, perpetual, irrevocable, royalty-free basis to BMJ Publishing Group Ltd ("BMJ") its licensees and where the relevant Journal is co-owned by BMJ to the co-owners of the Journal, to publish the Work in this journal and any other BMJ products and to exploit all rights, as set out in our licence.

The Submitting Author accepts and understands that any supply made under these terms is made by BMJ to the Submitting Author unless you are acting as an employee on behalf of your employer or a postgraduate student of an affiliated institution which is paying any applicable article publishing charge ("APC") for Open Access articles. Where the Submitting Author wishes to make the Work available on an Open Access basis (and intends to pay the relevant APC), the terms of reuse of such Open Access shall be governed by a Creative Commons licence – details of these licences and which Creative Commons licence will apply to this Work are set out in our licence referred to above.

Other than as permitted in any relevant BMJ Author's Self Archiving Policies, I confirm this Work has not been accepted for publication elsewhere, is not being considered for publication elsewhere and does not duplicate material already published. I confirm all authors consent to publication of this Work and authorise the granting of this licence.

For peer review only

The DEXA-CORT trial: study protocol of a randomized placebo-controlled trial of hydrocortisone in brain tumour patients on the prevention of neuropsychiatric adverse effects caused by perioperative dexamethasone

Anne-Sophie C.A.M. Koning[1], Djaina D. Satoer[2], Christiaan H. Vinkers[3,4], Amir H. Zamanipoor Najafabadi[5], Nienke R. Biermasz[1], Rishi D.S. Nandoe Tewarie[5], Wouter A. Moojen[5], Elisabeth F.C. van Rossum[6], Clemens M.F. Dirven[2], Alberto M. Pereira[1]*, Wouter R. van Furth[5]* and Onno C. Meijer[1]

*equal contribution

Affiliations:

[1] Department of Medicine, Division of Endocrinology, Leiden University Medical Center, Leiden, The Netherlands

[2] Department of Neurosurgery, Erasmus MC - University Medical Center, Rotterdam, The Netherlands

[3] Department of Psychiatry and Department of Anatomy and Neurosciences, Amsterdam University Medical Center, location VUMC, Amsterdam, The Netherlands

[4] Academic Working Place Depression, GGZ InGeest, Amsterdam, The Netherlands

[5] University Neurosurgical Center Holland, Leiden University Medical Center, Haaglanden Medical Center and Haga Teaching Hospitals, Leiden and The Hague, The Netherlands

[6] Department of Internal Medicine, Division of Endocrinology, Erasmus MC, University Medical Center Rotterdam, Rotterdam, The Netherlands

Corresponding author: Anne-Sophie C.A.M. Koning, a.c.a.koning@lumc.nl

Word count: Abstract: 282, Main text: 4753

For peer review only

For peer review only - http://bmjopen.bmj.com/site/about/guidelines.xhtml

ABSTRACT

Introduction: The synthetic glucocorticoid dexamethasone can induce serious neuropsychiatric adverse effects. Dexamethasone activates the glucocorticoid receptor (GR) but, unlike endogenous cortisol, not the mineralocorticoid receptor (MR). Moreover, dexamethasone suppresses cortisol production, thereby eliminating its MR binding. Consequently, GR overactivation combined with MR underactivation may contribute to the neuropsychiatric adverse effects of dexamethasone. The DEXA-CORT trial aims to re-activate the MR using cortisol to reduce neuropsychiatric adverse effects of dexamethasone treatment.

Methods and analysis: The DEXA-CORT study is a multi-centre, randomised, double-blind, placebo-controlled trial in adult patients who undergo elective brain tumour resection treated perioperatively with high doses of dexamethasone to minimize cerebral oedema. 180 patients are randomised between treatment with either twice daily 10mg hydrocortisone or placebo during dexamethasone treatment. The primary study outcome is the difference in proportion of patients scoring ≥3 points on at least one of the Brief Psychiatric Rating Scale (BPRS) questions 5 days postoperatively, or earlier at discharge. Secondary outcomes are neuropsychiatric symptoms, quality of sleep, health-related quality of life (HRQoL) and neurocognitive functioning at several time points postoperatively. Furthermore, neuropsychiatric history, (serious) adverse events (S(AEs)), prescribed (psychiatric) medication and referrals or evaluations of psychiatrist/psychologist, and laboratory measurements are assessed.

Ethics and dissemination: The study protocol has been approved by the Medical Research Ethics Committee of the Leiden University Medical Center, and by the Dutch competent authority, and by the Institutional Review Boards of the participating sites. It is an investigator-initiated study with financial support by The Netherlands Organisation for Health Research and Development (ZonMw) and the Dutch Brain Foundation. Results of the study will be submitted for publication in a peer-reviewed journal.

Trial registration number: NL6726 (Netherlands Trial Register); open for patient inclusion. EudraCT number 2017-003705-17.

For peer review only - http://bmjopen.bmj.com/site/about/guidelines.xhtml

Strengths and limitations of this study

- The DEXA-CORT study is the first double-blind, placebo-controlled intervention trial in brain tumour patients aiming to reduce neuropsychiatric adverse effects of perioperative dexamethasone.
- Use of validated outcome measurements.
- Evaluation of acute and potential long-term effects of the intervention.
- Duration of dexamethasone treatment differs between participants.
- Not all patients experience adverse effects following dexamethasone treatment, and patients without adverse effects are included.

For peer review only

For peer review only - http://bmjopen.bmj.com/site/about/guidelines.xhtml

INTRODUCTION

Synthetic glucocorticoids (GCs) are widely used across many different clinical disciplines, in particular immunology, pulmonology, neurosurgery, and oncology. Their potent anti-inflammatory and immunosuppressive effects and ability to control cerebral oedema ensures effective treatment (1-3). However, moderate to high doses of synthetic GCs frequently cause severe side effects (4). These include serious neuropsychiatric conditions such as sleep disturbances, delirium, depression, mania, psychosis and suicidality (5). Despite these mentioned problems, dexamethasone is a standard treatment in brain tumour patients for more than 50 years since no better alternatives are present (6-8). Evidently, these adverse effects negatively affect well-being and quality of life. In particular, depressive symptoms, a common problem in brain tumour patients, appeared to be an independent predictor for quality of life rating (9). The risks of glucocorticoid induced neuropsychiatric adverse effects in this and other patient groups is therefore serious (10). At present, there is no preventive treatment for these unwanted and serious neuropsychiatric adverse effects.

The naturally produced hormone cortisol acts on both the glucocorticoid receptor (GR) and mineralocorticoid receptor (MR), whereas the synthetic GC dexamethasone mainly activates GR with low or absent binding to the MR *in vivo* (11). The increased GR activity is mediating the therapeutic goal of the treatment, i.e., reduction of oedema. However, dexamethasone strongly suppresses endogenous cortisol production through GR-mediated negative feedback mechanisms in the hypothalamus-pituitary-adrenal (HPA) axis. (12, 13). The MR is also a prominent cortisol receptor in the brain, and its activation by cortisol binding strongly decreases after the HPA axis suppression (14). While low concentrations of dexamethasone poorly penetrate the blood-brain-barrier (15), higher clinically used doses do reach the brain. This has led to the hypothesis that, in addition to the strongly stimulated GR, reduced brain MR activity may underlie the neuropsychiatric adverse effects during dexamethasone treatment (16).

MR activity has indeed been related to psychopathology. Correlatively, decreased MR expression has been observed in several psychiatric disorders (17). Functionally, both pharmacological intervention studies and a genetic gain-of-function variant of MR seem to have protective effects (18-20). Therefore, we hypothesize that increasing MR activity with physiological doses of cortisol (= hydrocortisone) may prevent or substantially diminish the disrupting neuropsychiatric effects in brain tumour patients that receive high doses of perioperative dexamethasone, see Figure 1. Such a positive effect of adding hydrocortisone was previously found in a subgroup of children who experienced severe adverse effects after dexamethasone treatment for childhood leukaemia (21). Therefore, the aim of the present study is to investigate the effects of hydrocortisone replacement in adult brain

For peer review only - http://bmjopen.bmj.com/site/about/guidelines.xhtml

tumour patients, compared to placebo, as add-on to the treatment with dexamethasone, on the development of neuropsychiatric adverse effects that can be caused by dexamethasone.

For peer review only

For peer review only - http://bmjopen.bmj.com/site/about/guidelines.xhtml

METHODS ANDS ANALYSIS

This manuscript is written according to SPIRIT Standard Protocol Items: Recommendations for Interventional Trials (supplementary file S1) (22, 23).

Study design and setting

The DEXA-CORT study is a multicentre, randomized, double-blind, placebo-controlled trial investigating the effect of hydrocortisone replacement as co-treatment to reduce perioperative dexamethasone related neuropsychiatric adverse effects in patients with brain tumours undergoing elective neurosurgery. A total of 180 patients will be randomised in a 1:1 ratio, stratified per study centre and on tumour type. With randomisation we intend to equally distribute factors that can have an influence on our main outcome; neuropsychiatric effects. For example, the underlying pathology, the location of the tumour, extent of the surgery, and psychiatric history. The participating centres are Leiden University Medical Center (LUMC), Erasmus University Medical Center (Erasmus MC), Haaglanden Medical Center (HMC), and University Medical Center Utrecht (UMCU). The primary outcome measure is the proportion of patients with neuropsychiatric symptoms 5 days after surgery – or earlier if discharge is within 5 days – compared between the hydrocortisone and placebo treated group. The study is investigator-initiated with the LUMC as sponsor, and is funded by ZonMw and the Dutch Brain Foundation.

Eligibility criteria

Adult patients (≥18y) diagnosed with a brain tumour (glioma or meningioma) and undergoing elective surgical resection are eligible for inclusion. All patients receive high dose dexamethasone according to institutional protocols. Since peri-operative dexamethasone dosing schemes differ among the participating centres a minimal peri-operative cumulative dexamethasone dose of 24mg or more in 6 days is required. Initially, preoperative dexamethasone of more than one day was considered an exclusion criterium. During the course of the study it appeared that many patients, especially those with malignant tumours and severe oedema, had dexamethasone prescribed several days before the planned surgery. Consequently many patients were not eligible for study participation, threatening completion of inclusion. Hence this exclusion criterium was discarded with consent of the Data Safety Monitoring Board (DSMB). Detailed inclusion and exclusion criteria are presented in Figure 2.

Study outline

A timeline of the study is shown in Figure 3. Eligible patients are approached in an outpatient setting by specialized nurses in consultation with the treating neurosurgeon. The study is explained and

For peer review only - http://bmjopen.bmj.com/site/about/guidelines.xhtml

written information is provided (supplementary file S2 and S3). After written informed consent by the coordinating investigator or principle investigators, patient characteristics (age, gender, educational level, medical background, medication use, tumour type and tumour location) are recorded. The informed consent form includes consent for collection for blood sample and residual tumour tissue. Before start of intervention, a baseline study visit is planned that includes a structured psychiatric interview, different self-report questionnaires, and neurocognitive tests. A selection of the questionnaires can be filled out online via the web-based database program Castor Electronic Data Capture (EDC; Castor, Amsterdam, The Netherlands). The intervention starts on the day of hospital admission, concurrent with the dexamethasone treatment one day prior to planned surgery. 5 days postoperatively – or earlier at discharge – a structured psychiatric interview and neurocognitive screening are conducted, and questionnaires are filled out. The study medication is provided to the patients according to clinical care and is continued as long as dexamethasone is prescribed. A follow-up visit is planned 5 to 8 weeks postoperatively coinciding with the regular care assessment, at which a structured psychiatric interview and neurocognitive tests are performed and different questionnaires are filled out. Study visits take place at the medical centre of treatment. 2 weeks and 3 months postoperatively questionnaires can be filled out online via Castor EDC. Study duration from start of intervention until completion of the last questionnaires is 3 months. The study schedule is shown in Table 1. In case the baseline study visit is planned the day before surgery, no neurocognitive tests will be performed – instead a neurocognitive screening may be performed – and presentation of the questionnaires is depend on the patients' psychological and physical condition. To improve adherence the follow-up visits are coinciding with the regular care assessments.

Intervention

Patients will be treated according to routine clinical care, based on local treatment protocols. The investigational product is hydrocortisone with placebo as comparator, both produced by Tiofarma B.V., Oud-Beijerland, The Netherlands. The Clinical Pharmacy and Toxicology department of the LUMC will repack, label and randomize the study medication. 180 patients are randomised between treatment with either twice daily 10mg hydrocortisone or placebo, both orally administered concurrent with the full course of perioperative dexamethasone treatment; mostly with a dexamethasone taper schedule. Study medication is available for 20 days; assuming the slow dexamethasone taper schedule is used, treatment will continue on average for 15 days. In the event that dexamethasone treatment re-starts after completion of its taper schedule, study medication will not be available. The study medication is prescribed by the physician and administration details are recorded. Treatment dosage is targeted towards physiological cortisol replacement. During

For peer review only - http://bmjopen.bmj.com/site/about/guidelines.xhtml

hospitalisation the hydrocortisone or placebo is administered in the morning at 08:00hr and in the evening between 18:00hr and 20:00hr, because at these times dexamethasone is administered according to local standard procedures. To minimise the number of errors in medicine administrations we decided to adhere to the existing logistics. When participants go home and have to taper the dexamethasone, they receive a taper schedule from the nurses. Depending on the schedule, they receive further instructions on the hydrocortisone/placebo administration (both verbally and on paper). For manageability reasons we decided to always take the hydrocortisone/placebo at the first and the last time point of the dexamethasone administration. Study medication and information is handed out to the patients by the nurses, in consultation with the coordinating investigator. Patients will receive the amount of study medication they need to minimise medication errors. Drug accountability is checked by the coordinating investigator.

Allocation, randomisation and blinding

Randomisation is controlled by the Clinical Pharmacy and Toxicology department of LUMC using block randomisation in a 1:1 ratio stratified for each medical centre participating in the study, and for tumour type (glioma or meningioma) with block size of 10. New study participants are assigned to a randomisation number by the coordinating investigator. The coordinating investigator will contact the local pharmacy about the patient and its randomisation number. The local pharmacy assigns the randomisation number to a medication number and then the pharmacists know the treatment allocation. The randomisation list is stored at the department and will not be disclosed until all participants have completed the trial. Only in case of emergency and the knowledge of the assigned treatment is required the randomisation code can be broken. The study is blinded for everyone involved, except the pharmacy who allocates participants to treatment. In addition, hydrocortisone and placebo pills are identical in terms of appearance and taste ensuring blinding.

Assessments during the study

Assessments during the study include neuropsychiatric symptoms, quality of sleep, neurocognitive assessment, registration of (serious) adverse events ((S)AEs), prescribed medication, and referrals to psychiatrist/psychologist. We plan to evaluate potential long term follow-up effects on health-related quality of life (HRQoL). However, we did not include a specific measure for PTSD, even if a 'planned critical illness' like elective brain surgery may induce this disease (24). Furthermore, blood and residual tissue from tumour specimens will be collected to verify MR targeting and molecular effects of hydrocortisone co-treatment.

For peer review only

For peer review only - http://bmjopen.bmj.com/site/about/guidelines.xhtml

Neuropsychiatric symptoms

Brief Psychiatric Rating Scale (BPRS)

Neuropsychiatric symptoms will be measured with the BPRS version 4.0 (25). The BPRS is an interview, which provides a description of major symptom characteristics in psychiatric patients. The BPRS covers the presence and severity of 24 symptoms scored on a 7 point scale (1 = not present, 7 = extremely severe). Possible scores vary from 24 to 168. The BPRS will be used for our primary outcome.

Mini-screen

A mini-screen will be used to determine the patient's neuropsychiatric history. This mini-screen comprises 22 'Yes' or 'No' questions about different neuropsychiatric symptoms (26).

Hospital Anxiety and Depression Scale (HADS)

To determine the levels of anxiety and depression in patients, the HADS questionnaire will be used (27). The HADS comprises 14 items; seven related to anxiety and seven to depression. Each question has four different answer options, ranging from 0 to 3. Patients scoring 8-10 on the anxiety or depression domain are classified as possibly suffering from anxiety or depression, respectively, and a score ≥11 as definite anxiety or depression.

Positive and Negative Affect Schedule (PANAS)

To determine the positive and negative affect of patients the PANAS is used. The PANAS comprises a 20-item mood scale yielding 2 subscales; positive affect (PA) and negative affect (NA) (28). Patients rate how they felt 'in general' on a 5-point scale; 1 = very slightly or not at all, 2 = a little, 3 = moderately, 4 = quite a bit and 5 = very much. PA scores range from 10-50, with higher scores representing higher levels of positive affect. NA scores range form 10-50, with lower scores representing lower levels of negative affect.

The Altman Self-Rating Mania Scale (ASRM)

The ASRM is used to assess the presence and/or severity of manic symptoms (29). The ASRM consists of 5-items consisting of 5 statements. At the 5 items, the patient chooses one statement that best describes the way he or she has been feeling for the past week.

Neuropsychiatric Inventory Question (NPI-Q)

For peer review only - http://bmjopen.bmj.com/site/about/guidelines.xhtml

The NPI-Q is a questionnaire to be filled out by a family member or care-taker of the patient, that specifically assesses neuropsychiatric symptoms of the patient in the last month (30-32). The NPI-Q consist of 12 domains of symptoms. Every domain needs to be answered with 'yes' (is present) or 'no' (is absent). When the symptom is present, the partner rates the severity of the symptom (3-point scale) and the psychiatric burden (6-point scale) for him/herself. The NPI-Q gives a total score for the severity (range 0 – 36) and a total score for emotional burden (range 0 – 60).

Delirium Observation Screening (DOS)

The DOS scale is used to determine the presence of delirium in patients and includes 13 observation items of behaviour that show symptoms of delirium (33). Nurses measure the different items during the day, evening and night. Each item has 3 answer options; 'never', 'sometimes – always' and 'not known'. These 3 scores are then scored with 0, 1 or no points. The total DOS scale score is the sum of the total DOS score at day, evening and night divided by 3. Patients scoring ≥3 are probably suffering from delirium.

Quality of sleep

To determine the sleep quality the Leeds Sleep Evaluation Questionnaire (LSEQ) is used (34, 35). LSEQ contact information and permission to use is available from Mapi Research Trust, Lyon, France, https://eprovide.mapi-trust.org. LSEQ is a reliable instrument to assess sleep quality changes during psycho-pharmacological treatment intervention. This questionnaire is a self-report measure with 10-items consisting of a 10-cm-line visual analogue rating scale that evaluates four domains; ease of initiating sleep, quality of sleep, ease of waking and behaviour following wakefulness. The LSEQs 10-cm-horizontal line has 2 extreme states defined at the end of the line (e.g. Tired = score of 0, Alert = score of 10). Patients respond by placing a vertical mark on the line. Each question is scored out of 10 (e.g. 0 cm = score 0,5 cm = score of 5).

HRQoL

HRQoL will be measured with the EORTC core quality of life questionnaire (QLQ-C30) complemented with the QLQ-BN20 (36, 37). The QLQ-C30 is specific in assessing the HRQoL of cancer patients participating in clinical trials. It consists of 30 items, of which 28 have a four-point scale (1 = not at all, 4 = very much). 2 questions have a seven-point scale (1 = very poor, 7 = excellent). A high score represents a higher response level; high/healthy level of functioning, high QoL and a high level of symptoms. The QLQ-BN20 is a validated module specific for patients with brain neoplasms. It includes

For peer review only - http://bmjopen.bmj.com/site/about/guidelines.xhtml

20 items with a four-point scale (1 = not at all, 4 = very much) and the higher the score the more symptoms or problems.

Neurocognitive assessment

To assess the cognitive domains verbal memory, executive functioning and attention, we used the following standardized neurocognitive tests: HVLT-R – Free Recall (38), Trail Making Test A and B (39), Controlled Oral Word Association (40) and MOS Scale (41). Raw scores can be transformed into z-scores in order to compare patients' performance to control participants (and can be corrected for demographic variables, such as age and education).

The Montreal Cognitive Assessment (MoCA) is a cognitive screening instrument for mild cognitive dysfunction (42). It measures different cognitive domains; attention and concentration, executive functions, memory, language, visuo-constructional skills, conceptual thinking, calculations and orientation. The total score is 30 points; a score of ≥26 is a normal score.

The Aphasia Bedside Check (ABC) is a 10-min screening tool that globally determines the presence of aphasia with seven items for language comprehension and seven items for language production (43). This screening will only be performed in patients who have a left sided brain tumour with (hetero-) anamnestic language problems. With ≥3 mistakes an aphasia may be present and the patient should not be included in the study.

(Serious) Adverse Events ((S)AEs)– Medication – Referral to psychiatrist/psychologist

(S)AEs, prescribed medication (especially psychiatric medication) and referrals or evaluations of psychiatrist/psychologist will be recorded.

Laboratory measures

We try to collect 1 tube of blood taken during anaesthetic procedures for surgery. This will be used for DNA analyses to determine MR haplotype. Additionally, cortisol and glucose levels will be measured in blood.

If possible during the surgical procedure tumour tissue and peritumoral infiltrated brain tissue will be collected to investigate MR and GR and the effect of cortisol on these receptors.

Concomitant care and trial follow-up

Patient care is continued as usual. Participants are allowed to participate in other trials if they are not interfering with our intervention period. Participants may withdraw from the study for any reason at

For peer review only - http://bmjopen.bmj.com/site/about/guidelines.xhtml

any time. Premature study withdrawal will have no effect on regular patient care. All data collected up to withdrawal will be used for analysis. All participants will be followed until completion of the trial and will be notified of the results.

Primary outcome

The primary study outcome is the difference in proportion of patients scoring ≥3 points on at least one of the BPRS questions 5 days postoperatively – or earlier at discharge – between the hydrocortisone and placebo group. A better outcome on the BPRS in the hydrocortisone group would be considered a positive result, which would indicate that addition of hydrocortisone to dexamethasone is beneficial to reduce neuropsychiatric adverse effects of dexamethasone.

Secondary outcomes

Secondary outcomes are neuropsychiatric symptoms, quality of sleep, HRQoL, and neurocognitive functioning 5 days postoperatively – or earlier at discharge – , 2 weeks, 5 to 8 weeks, and 3 months postoperatively. Furthermore, neuropsychiatric history, SAEs, prescribed (psychiatric) medication and referrals or evaluations of psychiatrist/psychologist will be collected. In addition, the laboratory measurements are considered secondary outcomes.

Sample size

The sample size calculation is based on the results of a pilot study in which we tested the BPRS in our study population. We determined postoperative BPRS scores to evaluate the presence of psychiatric symptoms in brain tumour patients who underwent surgery and were treated with dexamethasone. We interviewed 18 patients on the day of hospital discharge. According to the BPRS scores in this group we considered a score of ≥3 on at least one of the BPRS items as clinically relevant for patients. We found that 7 out of 18 patients (≈40%) scored at least ≥3 on one of the BPRS items. Based on expert clinical judgement a 50% reduction in the patients with a score ≥3 is perceived as relevant. This equals an absolute difference of 20%. If we consider an $\alpha = 0.05$ and a power of 80%, and we take the 20% absolute difference between the 2 treatment arms, a sample size of 82 patients in each group would be needed. Based on an expected drop-out rate of ≈10%, 90 patients in each treatment arm (in total 180 patients) will be considered as a satisfactory sample size.

Planned statistical analyses

This study is a randomized controlled trial. All data will be analysed on an intention-to-treat basis.

BPRS

For peer review only - http://bmjopen.bmj.com/site/about/guidelines.xhtml

The primary study parameter is the proportion of patients scoring ≥3 points on at least one of the BPRS questions 5 days postoperatively – or earlier at discharge. The difference in these proportions between the placebo group and the hydrocortisone group will be calculated and statistically compared with the Chi-Square test. Any difference with a $p<0.05$ will be considered significant.

As secondary analyses, both treatment arms will be compared over time using analysis of repeated measures, generalized estimating equation (GEE) for the dichotomous outcomes as described in the primary outcome and the mixed linear model for the BPRS as continuous outcome. Additionally, we will look at the differences between the treatment arms on the separate items of the BRPS.

Mini-screen, HADS, PANAS, ASRM, NPI-Q, DOS, LSEQ and HRQoL

Both treatment arms will be compared over time using analysis of repeated measures, GEE when outcome is dichotomous and mixed linear models for continuous outcomes.

Explanatory univariable and multivariable regression analysis will be used to identify (baseline) predictors of treatment response. Sensitivity analysis will be performed using multiple imputation to handle missing data. Any difference with a $p<0.05$ will be considered significant. A better outcome on the different scales in the hydrocortisone group would be considered a positive result.

Neurocognitive functioning

The neurocognitive tests are a combination of neuropsychological tests for which normative data were published, so that patient's performance can be corrected according to age, gender and education. We will compare the raw data between the treatment arms with a t-test. Test scores can be transformed into z-values using normative data and compared with a one sample t-test. With no-normal distributions the appropriate statistical tests will be used.

Subgroup analyses

Subgroup analyses will be performed to explore differences in our primary and secondary parameters for type of tumour, gender, age and dosage of dexamethasone.

As the study remains blinded until completion, no interim analyses are planned. In addition, the statistical plan will be finalized before starting the analyses.

Data management

All data will be collected, stored, and analysed in a coded manner, and in accordance with the General Data Protection Regulation (GDPR). Data are collected and stored in the web-based database

For peer review only - http://bmjopen.bmj.com/site/about/guidelines.xhtml

management system Castor EDC using electronic case report forms (CRFs). The CRFs have built-in data validity and value range checks. Source documents include medical records, paper CRFs, paper interviews, questionnaires and neuropsychological tests. All data is pseudonymised with a study code and will be stored for 15 years. The subject identification log which links subjects to the code is kept in a trial file only accessible to study personnel. Possible blood samples and residual tumour tissue obtained for standard care and obtained following local consent forms during the study will be stored according to local regulatory guidelines.

Safety

Participating in the trial is very unlikely to bring additional risks for patients, since hydrocortisone is given in a low dose to restore the physiological cortisol level in the body.

DSMB

This study established a DSMB that protects and guarantees patients safety during the trial. The DSMB includes 3 members with different study backgrounds: a statistician, psychiatrist and an internist/clinical epidemiologist. The role and responsibilities of the DSMB is described in the DSMB charter document.

AEs and SAEs

The study population has a high risk of complications, which are inherent to their vulnerable condition (neurological deficits caused by a brain tumour, surgery, high dose dexamethasone) and these are considered unrelated to the intervention. The patient population is expected to encounter many AEs not related to the experimental design, therefore not all AE will be registered. AEs that will be registered are: psychiatric complaints of which psychiatric consultation and medication is needed, brain oedema for which increased dexamethasone medication is given, or for which the dexamethasone reduction scheme is extended, all infections for which antibiotics are needed, cerebrospinal fluid (CSF) leakage that requires surgical intervention, hyperglycaemia for which medication is needed, cardiovascular events requiring intervention, epileptic seizures requiring intervention and secondary adrenal insufficiency that requires hydrocortisone suppletion. The abovementioned AEs will be registered from the start of the intervention until completion of the trial.

SAEs that we will record are any untoward medical occurrence or effect that results in death, is life threatening (at the time of the event), requires hospitalisation or prolongation of existing inpatients' hospitalisation – including intensive care – when a patient has unexpected longer IC admission or unexpected >10 days hospitalisation, results in persistent or significant disability or incapacity – like

For peer review only - http://bmjopen.bmj.com/site/about/guidelines.xhtml

neurological dysfunction – lasting for more than 3 days postoperatively, results in any other important medical event not mentioned above due to medical or surgical intervention and judged as such by the investigators and post-operative haemorrhage for which re-craniotomy is needed. An elective hospital admission will not be considered a SAE. Whether related to the study medication or not, all these SAEs occurring during the intervention period will be reported through the web portal governed and issued by the Dutch National Ethics Committee (CCMO) called 'ToetsingOnline'. SAEs occurring after the intervention period will be line-listed and not reported in ToetsingsOnline.

Monitoring

According to Good Research Practice (GRP) independent on-site monitoring visits will be performed to ensure the conduct of the trial is consistent with the approved protocol(s), the rights and well-being of the subjects are protected, source documents are verified and data is accurately reported from the source documents. Monitoring visits will take place according to the study specific monitoring plan.

Patients and public involvement

Patients and public were not involved in the study design. The ZonMw committee that granted the funding includes a representative of the patients interests. The committee is kept informed annually.

For peer review only

For peer review only - http://bmjopen.bmj.com/site/about/guidelines.xhtml

ETHICS AND DISSEMINATION

The study protocol has been approved by the accredited Medical Research Ethics Committee of the LUMC, and by the Dutch competent authority, and by the Institutional Review Boards of the participating sites. Substantial amendments to the protocol will first be sent for approval and upon consent the participating centres and trial registries will be informed. The study will be conducted according to the principles of the Declaration of Helsinki, Good Clinical Practice guidelines – including centre specific requirements – and in accordance with the Dutch Medical Research Involving Human Subjects Act (WMO). Participant WMO subject insurance is provided by the LUMC for all participating subjects and liability insurances are provided by all participating centres. This trial is registered in the Dutch Trial Register (NTR). Regardless of the outcome, trial results will be submitted for publication in a peer-reviewed journal and presented at conferences. Publication rights and ownership of data is secured in a clinical trial agreement with participating centres. Full protocol, data sets and statistical analyses will be available in a repository with a persistent identifier after publication of the final results.

Trial status

The first patient was included on 10 September 2019 and recruitment is ongoing. Inclusion rate has slowed down due to the COVID-19 pandemic from March 2020 onwards. End of recruitment is expected to be 2022. The current study protocol version is V3, August 28. 2020.

For peer review only

DISCUSSION

The DEXA-CORT trial is the first double-blind, placebo-controlled trial in adult brain tumour patients, in which we investigate the possible prevention of neuropsychiatric adverse effects of perioperative dexamethasone treatment. Dexamethasone is a well-known very potent activator of the GR and consequently suppresses endogenous adrenocortical cortisol production via negative feedback on the HPA-axis, depleting brain MR from its ligand (12-14). The resultant disturbed balance in receptor activation in the brain may predispose for psychopathology. In this study, we test the hypothesis that re-instating brain MR activity via co-treatment with a physiological dose of cortisol (=hydrocortisone) will improve (specific features of) neuropsychological well-being. Because of the high dexamethasone doses and high affinity of dexamethasone for GR, cortisol treatment will only affect MR occupancy and not interfere with the therapeutic goal of dexamethasone treatment, which is GR activation.

Adverse neuropsychiatric effects can also be present with the use of other GCs, like prednisone and prednisolone. Like dexamethasone, prednisone and prednisolone suppress the HPA-axis, which would subsequently deplete MR from its ligand. In addition, compared to the naturally produced cortisol, the synthetic GCs dexamethasone, prednisone, and prednisolone all have much lower MR binding potency (11). An important difference between dexamethasone and the other mentioned GCs is its higher GR potency, its long half-life and lower MR potency. The potency differences could be of influence on the adverse effects; higher GR potency could maybe suppress the HPA-axis faster and longer half-life might extend its effects. Lower MR potency might accentuate the effect of a suppressed HPA-axis. It would be interesting to investigate the difference in neuropsychiatric effects between different GCs, and (eventually) benefit of hydrocortisone add on.

This cortisol re-fill approach was already performed in childhood leukaemia patients. Patients who experienced severe adverse effects of dexamethasone seemed to benefit from the additional hydrocortisone medication (21). To establish this potential positive effect in an adult population, the DEXA-CORT trial is investigating this principle in adult brain tumour patients. Dexamethasone is a standard clinical treatment in brain tumour patients perioperatively and adverse effects are also present in this patient group, which leads to reduced HRQoL. A methodological drawback of our study population is the lack of a cross-over design. Rather, the high doses, but brief perioperative use of dexamethasone formed the rational for testing our hypothesis in this patient group. If additional hydrocortisone to dexamethasone proves effective in reducing its neuropsychiatric adverse effects, it would offer a new treatment option for this patient group and maybe even for many other patients that receive long-term treatment with dexamethasone, and other synthetic GCs. An interesting question that will follow is how long to continue the hydrocortisone replacement. After

For peer review only - http://bmjopen.bmj.com/site/about/guidelines.xhtml

dexamethasone treatment, the endogenous HPA-axis will need a recovery period. It might be beneficial to continue the additional hydrocortisone for at least some days after dexamethasone is stopped.

For peer review only

For peer review only - http://bmjopen.bmj.com/site/about/guidelines.xhtml

Supplementary files

Supplementary file S1: Completed SPIRIT Checklist.

Supplementary file S2: Patient information letter and informed consent, Dutch LUMC version.

Supplementary file S3: Patient information letter and informed consent, English LUMC version.

Footnotes

Contributors: OCM was main applicant in the funding acquisition. ACAM, DDS, AHZN, NRB, EFCR, CHV, CMFD, AMP, WRF and OCM participated in the design of the study. CMFD, DDS, AMP, WRF and RDSN are principal investigators at the participating study centres. CMFD, DDS, RDSN, WAM, ACAM initiated and implemented the study design at the participating study centre. ACAM is the coordinating investigator with DDS as daily supervisor. ACAM drafted the manuscript. All authors reviewed and approved the final manuscript.

Funding: This work is supported by The Netherlands Organisation for Health Research and Development (ZonMw) and Dutch Brain Foundation grant project number 95105005. The DEXA-CORT study is investigator-initiated; Leiden University Medical Center (LUMC) is the sponsor of the study. The funding party did play no role in the final study design or study conduct nor in the manuscript preparation of decision to submit for publication.

Competing interests: Authors have nothing to disclose.

Ethics approval: Medical Research Ethics Committee of the LUMC (reference: NL63350.058.18)

Trial registration: EudraCT: 2017-003705-17, registered 12 September 2017; Netherlands Trial Register (NTR): NL6726, registered 18 January 2018. Registration took place before start of recruitment.

Protocol versions:

- V2.0, February 28 2019: first approved protocol version
- V3.0, August 28 2020: study medication use changed from 10 to 15 days, addition of informed consent form for the partner/family member of the participant. Small protocol changes: better description of tracking of AE and SAE, acceptance of 1 mistake in study medication administration and replacing excluded study participants. Also extension of study duration.

Data sharing statement: The datasets generated and/or analysed during the current study will be available in a repository with a persistent identifier after publication of the final results.

For peer review only - http://bmjopen.bmj.com/site/about/guidelines.xhtml

REFERENCES

1. Ismail MF, Lavelle C, Cassidy EM. Steroid-induced mental disorders in cancer patients: a systematic review. Future Oncol. 2017;13(29):2719-31.
2. Hockey B, Leslie K, Williams D. Dexamethasone for intracranial neurosurgery and anaesthesia. J Clin Neurosci. 2009;16(11):1389-93.
3. Arvold ND, Armstrong TS, Warren KE, Chang SM, DeAngelis LM, Blakeley J, et al. Corticosteroid use endpoints in neuro-oncology: Response Assessment in Neuro-Oncology Working Group. Neuro Oncol. 2018;20(7):897-906.
4. Strehl C, Bijlsma JW, de Wit M, Boers M, Caeyers N, Cutolo M, et al. Defining conditions where long-term glucocorticoid treatment has an acceptably low level of harm to facilitate implementation of existing recommendations: viewpoints from an EULAR task force. Ann Rheum Dis. 2016;75(6):952-7.
5. Judd LL, Schettler PJ, Brown ES, Wolkowitz OM, Sternberg EM, Bender BG, et al. Adverse consequences of glucocorticoid medication: psychological, cognitive, and behavioral effects. Am J Psychiatry. 2014;171(10):1045-51.
6. Wen PY, Schiff D, Kesari S, Drappatz J, Gigas DC, Doherty L. Medical management of patients with brain tumors. J Neurooncol. 2006;80(3):313-32.
7. Galicich JH, French LA, Melby JC. Use of dexamethasone in treatment of cerebral edema associated with brain tumors. J Lancet. 1961;81:46-53.
8. Dietrich J, Rao K, Pastorino S, Kesari S. Corticosteroids in brain cancer patients: benefits and pitfalls. Expert Rev Clin Pharmacol. 2011;4(2):233-42.
9. Pelletier G, Verhoef MJ, Khatri N, Hagen N. Quality of life in brain tumor patients: the relative contributions of depression, fatigue, emotional distress, and existential issues. J Neurooncol. 2002;57(1):41-9.
10. Fardet L, Petersen I, Nazareth I. Suicidal behavior and severe neuropsychiatric disorders following glucocorticoid therapy in primary care. Am J Psychiatry. 2012;169(5):491-7.
11. Grossmann C, Scholz T, Rochel M, Bumke-Vogt C, Oelkers W, Pfeiffer AF, et al. Transactivation via the human glucocorticoid and mineralocorticoid receptor by therapeutically used steroids in CV-1 cells: a comparison of their glucocorticoid and mineralocorticoid properties. Eur J Endocrinol. 2004;151(3):397-406.
12. Spencer RL, Deak T. A users guide to HPA axis research. Physiol Behav. 2017;178:43-65.
13. De Kloet R, Wallach G, McEwen BS. Differences in corticosterone and dexamethasone binding to rat brain and pituitary. Endocrinology. 1975;96(3):598-609.
14. Karssen AM, Meijer OC, Berry A, Sanjuan Pinol R, de Kloet ER. Low doses of dexamethasone can produce a hypocorticosteroid state in the brain. Endocrinology. 2005;146(12):5587-95.
15. Meijer OC, de Lange EC, Breimer DD, de Boer AG, Workel JO, de Kloet ER. Penetration of dexamethasone into brain glucocorticoid targets is enhanced in mdr1A P-glycoprotein knockout mice. Endocrinology. 1998;139(4):1789-93.
16. Meijer OC, de Kloet ER. A Refill for the Brain Mineralocorticoid Receptor: The Benefit of Cortisol Add-On to Dexamethasone Therapy. Endocrinology. 2017;158(3):448-54.
17. ter Heegde F, De Rijk RH, Vinkers CH. The brain mineralocorticoid receptor and stress resilience. Psychoneuroendocrinology. 2015;52:92-110.
18. Klok MD, Giltay EJ, Van der Does AJ, Geleijnse JM, Antypa N, Penninx BW, et al. A common and functional mineralocorticoid receptor haplotype enhances optimism and protects against depression in females. Transl Psychiatry. 2011;1:e62.
19. Otte C, Hinkelmann K, Moritz S, Yassouridis A, Jahn H, Wiedemann K, et al. Modulation of the mineralocorticoid receptor as add-on treatment in depression: a randomized, double-blind, placebo-controlled proof-of-concept study. J Psychiatr Res. 2010;44(6):339-46.

For peer review only - http://bmjopen.bmj.com/site/about/guidelines.xhtml

20. Otte C, Wingenfeld K, Kuehl LK, Kaczmarczyk M, Richter S, Quante A, et al. Mineralocorticoid receptor stimulation improves cognitive function and decreases cortisol secretion in depressed patients and healthy individuals. Neuropsychopharmacology. 2015;40(2):386-93.
21. Warris LT, van den Heuvel-Eibrink MM, Aarsen FK, Pluijm SM, Bierings MB, van den Bos C, et al. Hydrocortisone as an Intervention for Dexamethasone-Induced Adverse Effects in Pediatric Patients With Acute Lymphoblastic Leukemia: Results of a Double-Blind, Randomized Controlled Trial. J Clin Oncol. 2016;34(19):2287-93.
22. Chan AW, Tetzlaff JM, Altman DG, Laupacis A, Gotzsche PC, Krleza-Jeric K, et al. SPIRIT 2013 Statement: Defining Standard Protocol Items for Clinical Trials. Ann Intern Med. 2013;158(3):200-+.
23. Chan AW, Tetzlaff JM, Gotzsche PC, Altman DG, Mann H, Berlin JA, et al. SPIRIT 2013 explanation and elaboration: guidance for protocols of clinical trials. Bmj-Brit Med J. 2013;346.
24. Franks ZM, Alcock JA, Lam T, Haines KJ, Arora N, Ramanan M. Physical Restraints and Post-Traumatic Stress Disorder in Survivors of Critical Illness A Systematic Review and Meta-analysis. Ann Am Thorac Soc. 2021;18(4):689-97.
25. Ventura J, Lukoff D, Nuechterlein KH. Brief Psychiatric Rating Scale (BPRS) Expanded Version (4.0): Scales, Anchor Points and Administration Manual. . International Journal of Methods in Psychiatric Research. 1993;3:227-44.
26. Sheehan DV, Lecrubier Y, Sheehan KH, Amorim P, Janavs J, Weiller E, et al. The Mini-International Neuropsychiatric Interview (M.I.N.I.): the development and validation of a structured diagnostic psychiatric interview for DSM-IV and ICD-10. J Clin Psychiatry. 1998;59 Suppl 20:22-33;quiz 4-57.
27. Zigmond AS, Snaith RP. The hospital anxiety and depression scale. Acta Psychiatr Scand. 1983;67(6):361-70.
28. Watson D, Clark LA, Tellegen A. Development and validation of brief measures of positive and negative affect: the PANAS scales. J Pers Soc Psychol. 1988;54(6):1063-70.
29. Altman EG, Hedeker D, Peterson JL, Davis JM. The Altman Self-Rating Mania Scale. Biol Psychiatry. 1997;42(10):948-55.
30. Cummings JL, Mega M, Gray K, Rosenberg-Thompson S, Carusi DA, Gornbein J. The Neuropsychiatric Inventory: comprehensive assessment of psychopathology in dementia. Neurology. 1994;44(12):2308-14.
31. Kaufer DI, Cummings JL, Ketchel P, Smith V, MacMillan A, Shelley T, et al. Validation of the NPI-Q, a brief clinical form of the Neuropsychiatric Inventory. J Neuropsychiatry Clin Neurosci. 2000;12(2):233-9.
32. de Jonghe JF, Kat MG, Kalisvaart CJ, Boelaarts L. [Neuropsychiatric inventory questionnaire (NPI-Q): A validity study of the Dutch form]. Tijdschr Gerontol Geriatr. 2003;34(2):74-7.
33. Schuurmans MJ, Shortridge-Baggett LM, Duursma SA. The Delirium Observation Screening Scale: a screening instrument for delirium. Res Theory Nurs Pract. 2003;17(1):31-50.
34. Parrott AC, Hindmarch I. Factor analysis of a sleep evaluation questionnaire. Psychol Med. 1978;8(2):325-9.
35. Hindmarch I. A 1,4-benzodiazepine, temazepam (K 3917), its effect on some psychological parameters of sleep and behaviour. Arzneimittelforschung. 1975;25(11):1836-9.
36. Aaronson NK, Ahmedzai S, Bergman B, Bullinger M, Cull A, Duez NJ, et al. The European Organization for Research and Treatment of Cancer QLQ-C30: a quality-of-life instrument for use in international clinical trials in oncology. J Natl Cancer Inst. 1993;85(5):365-76.
37. Taphoorn MJ, Claassens L, Aaronson NK, Coens C, Mauer M, Osoba D, et al. An international validation study of the EORTC brain cancer module (EORTC QLQ-BN20) for assessing health-related quality of life and symptoms in brain cancer patients. Eur J Cancer. 2010;46(6):1033-40.
38. Brandt J, Benedict RHB. HVLT-R: Hopkins Verbal Learning Test-Revised. Copyright 1991, 1998, 2001 by PAR, Inc
39. Tombaugh TN. Trail Making Test A and B: normative data stratified by age and education. Arch Clin Neuropsychol. 2004;19(2):203-14.

For peer review only - http://bmjopen.bmj.com/site/about/guidelines.xhtml

565 40. Benton AL, Hamsher KdS. Multilingual Aphasia Examination. Copyright 1978 by PAR, Inc.
566 41. Stewart AL, Greenfield S, Hays RD, Wells K, Rogers WH, Berry SD, et al. Functional Status and Well-Being of Patients with Chronic Conditions - Results from the Medical Outcomes Study. Jama-J Am Med Assoc. 1989;262(7):907-13.
569 42. Nasreddine ZS, Phillips NA, Bedirian V, Charbonneau S, Whitehead V, Collin I, et al. The Montreal Cognitive Assessment, MoCA: a brief screening tool for mild cognitive impairment. J Am Geriatr Soc. 2005;53(4):695-9.
572 43. Visch-Brink E, El Hachioui H. *Afasie Bedside Check*. Erasmus MC, Rotterdam, Nederland. 2013.

573

574

For peer review only

For peer review only - http://bmjopen.bmj.com/site/about/guidelines.xhtml

TABLES

Table 1. The DEXA-CORT study schedule of enrolment, intervention and assessments.

	**STUDY PERIOD**								
	**Outpatient clinic**	**Baseline**	**Treatment**				**Follow-up**	**Follow-up**	**Close-out**
			**admission**	**surgery**	**ward**	**discharge**			
**Days**		-14 – -1	-1	0	1 – 4	3 – 5	14	35 – 56	84
**Visits**	V_0	V_1	V_1/V_2					V_2/V_3	
**Enrolment:**									
Eligibility screen	X								
Informed consent		X	X						
Baseline characteristics		X	X						
Allocation		X	X						
**Interventions:**									
Hydrocortisone			X	→	→	→	→		
Placebo			X	→	→	→	→		
**Assessments:**									
BPRS		X	X			X		X	
Mini-screen		X	X						
HADS		X	(X)				X		X
PANAS		X	(X)		X	X	X	X	X
ASRM		X	(X)				X		X
NPI-Q		X	X			X		X	
DOS					X				
LSEQ		X	(X)			X	X	X	X
QoL-C30/BN20		X	(X)						X
Cognitive tests		X						X	
MoCA			(X)			X			
ABC		X	X			X			
SAEs			X	→	→	→	→		

For peer review only

AEs		X								X
Medication		X								X
Psychiatric referrals		X								X
**Laboratory measures:**										
Tube of blood				X						
Residual tumour tissue				X						

BPRS: Brief Psychiatric Rating Scale, HADS: Hospital Anxiety and Depression Scale, PANAS: Positive And Negative Affect Schedule, ASRM: Altman Self-Rating Mania Scale, NPI-Q: Neuropsychiatric Inventory Question, DOS: Delirium Observation Screening, LSEQ: Leeds Sleep Evaluation Questionnaire, QoL: Quality of Life questionnaires, MoCA: Montreal Cognitive Assessment, ABC: Aphasia Bedside Check, (S)AEs: (Serious) Adverse Events.

For peer review only

FIGURE LEGENDS

Figure 1: Preventing neuropsychiatric adverse effects of dexamethasone with co-administration of cortisol. Dexamethasone suppresses the HPA-axis and thereby the adrenocortical cortisol production, leaving cortisol-preferring MRs unoccupied. Re-activating the MR using cortisol might reduce or prevent neuropsychiatric adverse effects of dexamethasone treatment via activation of brain MR.

Figure 2: Eligibility criteria of the DEXA-CORT study.

Figure 3: The DEXA-CORT study timeline.

For peer review only

For peer review only - http://bmjopen.bmj.com/site/about/guidelines.xhtml

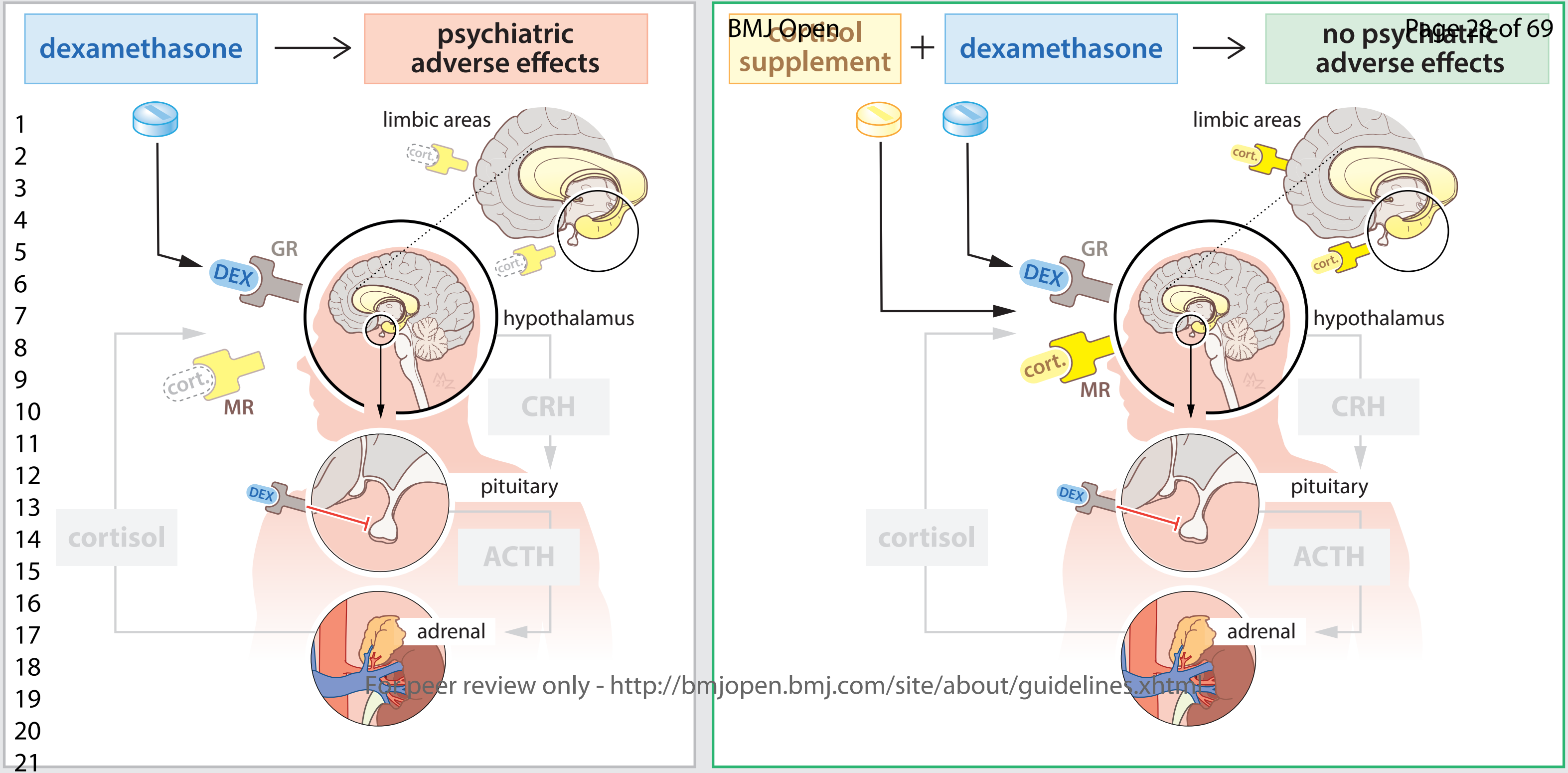

For peer review only - http://bmjopen.bmj.com/site/about/guidelines.xhtml

Inclusion criteria:

- Age = ≥18 years
- Cranial glioma or meningioma patients scheduled to undergo surgery (resection)
- Minimal dose of peri-operative cumulative dexamethasone exposure of 24mg or more in 6 days
- Good clinical condition; KPS ≥70
- Life expectancy ≥ 6 months

Exclusion criteria:

- Non-native speakers of Dutch or insufficient command of the Dutch language
- Patients that are unable to overview consequences of trial participation
- Patients with severe aphasia
- Patients that are not able to fill in the questionnaires because of cognitive impairments at the discretion of the physician
- Patients with psychiatric diseases or neurological deficits that interfere with the study to the judgement of treating physician
- Patients with prior diagnosis of secondary adrenal insufficiency/already taking hydrocortisone

For peer review only

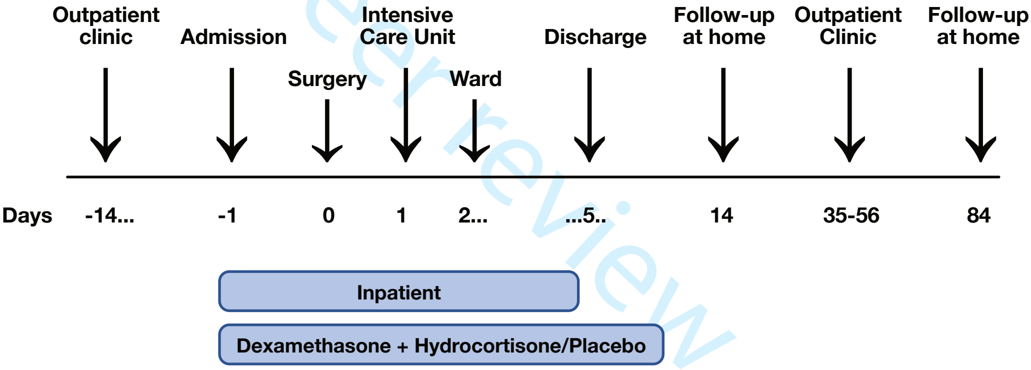

For peer review only

STANDARD PROTOCOL ITEMS: RECOMMENDATIONS FOR INTERVENTIONAL TRIALS

Manuscript Koning *et al.* 'The DEXA-CORT trial: study protocol of a randomized placebo-controlled trial of hydrocortisone in brain tumour patients on the prevention of neuropsychiatric adverse effects caused by perioperative dexamethasone.'

For peer review only

SPIRIT 2013 Checklist: Recommended items to address in a clinical trial protocol and related documents*

Section/item	Item No	Description	Addressed on page number
**Administrative information**			
Title	1	Descriptive title identifying the study design, population, interventions, and, if applicable, trial acronym	Title page, p1
Trial registration	2a	Trial identifier and registry name. If not yet registered, name of intended registry	Abstract, p2
	2b	All items from the World Health Organization Trial Registration Data Set	N/A (see registry)
Protocol version	3	Date and version identifier	Footnotes, p19
Funding	4	Sources and types of financial, material, and other support	Footnotes, p19
Roles and responsibilities	5a	Names, affiliations, and roles of protocol contributors	Title page, p1; Footnotes, p19
	5b	Name and contact information for the trial sponsor	Title page, p1
	5c	Role of study sponsor and funders, if any, in study design; collection, management, analysis, and interpretation of data; writing of the report; and the decision to submit the report for publication, including whether they will have ultimate authority over any of these activities	Ethics and Dissemination, p16; Footnotes, p19

For peer review only - http://bmjopen.bmj.com/site/about/guidelines.xhtml

	5d	Composition, roles, and responsibilities of the coordinating centre, steering committee, endpoint adjudication committee, data management team, and other individuals or groups overseeing the trial, if applicable (see Item 21a for data monitoring committee)	N/A
**Introduction**			
Background and rationale	6a	Description of research question and justification for undertaking the trial, including summary of relevant studies (published and unpublished) examining benefits and harms for each intervention	Introduction, p4
	6b	Explanation for choice of comparators	Intervention, p7-8
Objectives	7	Specific objectives or hypotheses	Introduction, p4
Trial design	8	Description of trial design including type of trial (eg, parallel group, crossover, factorial, single group), allocation ratio, and framework (eg, superiority, equivalence, noninferiority, exploratory)	Study design and setting, p6
**Methods: Participants, interventions, and outcomes**			
Study setting	9	Description of study settings (eg, community clinic, academic hospital) and list of countries where data will be collected. Reference to where list of study sites can be obtained	Study design and setting, p6
Eligibility criteria	10	Inclusion and exclusion criteria for participants. If applicable, eligibility criteria for study centres and individuals who will perform the interventions (eg, surgeons, psychotherapists)	Eligibility criteria, p6; Figure 2
Interventions	11a	Interventions for each group with sufficient detail to allow replication, including how and when they will be administered	Intervention, p7-8;
	11b	Criteria for discontinuing or modifying allocated interventions for a given trial participant (eg, drug dose change in response to harms, participant request, or improving/worsening disease)	Concomitant care and trial follow-up, p11
	11c	Strategies to improve adherence to intervention protocols, and any procedures for monitoring adherence (eg, drug tablet return, laboratory tests)	Study outline, p6-7; Intervention, p7-8

For peer review only - http://bmjopen.bmj.com/site/about/guidelines.xhtml

	11d	Relevant concomitant care and interventions that are permitted or prohibited during the trial	Concomitant care and trial follow-up, p11
Outcomes	12	Primary, secondary, and other outcomes, including the specific measurement variable (eg, systolic blood pressure), analysis metric (eg, change from baseline, final value, time to event), method of aggregation (eg, median, proportion), and time point for each outcome. Explanation of the clinical relevance of chosen efficacy and harm outcomes is strongly recommended	Assessments during study, p8; Outcomes, p12
Participant timeline	13	Time schedule of enrolment, interventions (including any run-ins and washouts), assessments, and visits for participants. A schematic diagram is highly recommended (see Figure)	Study outline, p6-7; Figure 3;Table 1
Sample size	14	Estimated number of participants needed to achieve study objectives and how it was determined, including clinical and statistical assumptions supporting any sample size calculations	Sample size, p12
Recruitment	15	Strategies for achieving adequate participant enrolment to reach target sample size	Study outline, p6-7
**Methods: Assignment of interventions (for controlled trials)**			
Allocation:			
Sequence generation	16a	Method of generating the allocation sequence (eg, computer-generated random numbers), and list of any factors for stratification. To reduce predictability of a random sequence, details of any planned restriction (eg, blocking) should be provided in a separate document that is unavailable to those who enrol participants or assign interventions	Allocation, randomisation and blinding, p8
Allocation concealment mechanism	16b	Mechanism of implementing the allocation sequence (eg, central telephone; sequentially numbered, opaque, sealed envelopes), describing any steps to conceal the sequence until interventions are assigned	Allocation, randomisation and blinding, p8
Implementation	16c	Who will generate the allocation sequence, who will enrol participants, and who will assign participants to interventions	Allocation, randomisation and blinding, p8
Blinding (masking)	17a	Who will be blinded after assignment to interventions (eg, trial participants, care providers, outcome assessors, data analysts), and how	Allocation, randomisation and blinding, p8

For peer review only - http://bmjopen.bmj.com/site/about/guidelines.xhtml

	17b	If blinded, circumstances under which unblinding is permissible, and procedure for revealing a participant's allocated intervention during the trial	Allocation, randomisation and blinding, p8
**Methods: Data collection, management, and analysis**			
Data collection methods	18a	Plans for assessment and collection of outcome, baseline, and other trial data, including any related processes to promote data quality (eg, duplicate measurements, training of assessors) and a description of study instruments (eg, questionnaires, laboratory tests) along with their reliability and validity, if known. Reference to where data collection forms can be found, if not in the protocol	Study outline, p6-7; Assessments, p8-11; Outcomes, p12; Data management, p13-14
	18b	Plans to promote participant retention and complete follow-up, including list of any outcome data to be collected for participants who discontinue or deviate from intervention protocols	Concomitant care and trial follow-up, p11; Study outline, p6-7; Intervention, p7-8
Data management	19	Plans for data entry, coding, security, and storage, including any related processes to promote data quality (eg, double data entry; range checks for data values). Reference to where details of data management procedures can be found, if not in the protocol	Data management, p13-14
Statistical methods	20a	Statistical methods for analysing primary and secondary outcomes. Reference to where other details of the statistical analysis plan can be found, if not in the protocol	Planned statistical analyses, p12-13
	20b	Methods for any additional analyses (eg, subgroup and adjusted analyses)	Planned statistical analyses, p12-13
	20c	Definition of analysis population relating to protocol non-adherence (eg, as randomised analysis), and any statistical methods to handle missing data (eg, multiple imputation)	Planned statistical analyses, p12-13
**Methods: Monitoring**			

Data monitoring	21a	Composition of data monitoring committee (DMC); summary of its role and reporting structure; statement of whether it is independent from the sponsor and competing interests; and reference to where further details about its charter can be found, if not in the protocol. Alternatively, an explanation of why a DMC is not needed	N/A (no DMC), Monitoring, p15
	21b	Description of any interim analyses and stopping guidelines, including who will have access to these interim results and make the final decision to terminate the trial	N/A (no interim analyses)
Harms	22	Plans for collecting, assessing, reporting, and managing solicited and spontaneously reported adverse events and other unintended effects of trial interventions or trial conduct	Assessments, p11; Safety, p14
Auditing	23	Frequency and procedures for auditing trial conduct, if any, and whether the process will be independent from investigators and the sponsor	Monitoring, p15
**Ethics and dissemination**			
Research ethics approval	24	Plans for seeking research ethics committee/institutional review board (REC/IRB) approval	Ethics and Dissemination, p16
Protocol amendments	25	Plans for communicating important protocol modifications (eg, changes to eligibility criteria, outcomes, analyses) to relevant parties (eg, investigators, REC/IRBs, trial participants, trial registries, journals, regulators)	Ethics and Dissemination, p16
Consent or assent	26a	Who will obtain informed consent or assent from potential trial participants or authorised surrogates, and how (see Item 32)	Study outline, p6-7
	26b	Additional consent provisions for collection and use of participant data and biological specimens in ancillary studies, if applicable	Study outline, p6-7
Confidentiality	27	How personal information about potential and enrolled participants will be collected, shared, and maintained in order to protect confidentiality before, during, and after the trial	Data management, p13-14
Declaration of interests	28	Financial and other competing interests for principal investigators for the overall trial and each study site	Footnotes, p19

peer review only

Access to data	29	Statement of who will have access to the final trial dataset, and disclosure of contractual agreements that limit such access for investigators	Ethics and Dissemination, p16; Footnotes, p19
Ancillary and post-trial care	30	Provisions, if any, for ancillary and post-trial care, and for compensation to those who suffer harm from trial participation	Ethics and Dissemination, p16
Dissemination policy	31a	Plans for investigators and sponsor to communicate trial results to participants, healthcare professionals, the public, and other relevant groups (eg, via publication, reporting in results databases, or other data sharing arrangements), including any publication restrictions	Concomitant care and trial follow-up, p11; Ethics and Dissemination, p16
	31b	Authorship eligibility guidelines and any intended use of professional writers	Ethics and Dissemination, p16
	31c	Plans, if any, for granting public access to the full protocol, participant-level dataset, and statistical code	Ethics and Dissemination, p16
**Appendices**			
Informed consent materials	32	Model consent form and other related documentation given to participants and authorised surrogates	Supplementary file S2 and S3
Biological specimens	33	Plans for collection, laboratory evaluation, and storage of biological specimens for genetic or molecular analysis in the current trial and for future use in ancillary studies, if applicable	Data management, p13-14; Assessments, p11

*It is strongly recommended that this checklist be read in conjunction with the SPIRIT 2013 Explanation & Elaboration for important clarification on the items. Amendments to the protocol should be tracked and dated. The SPIRIT checklist is copyrighted by the SPIRIT Group under the Creative Commons "Attribution-NonCommercial-NoDerivs 3.0 Unported" license.

For peer review only

The

DEXA-CORT

Trial

For peer review only - http://bmjopen.bmj.com/site/about/guidelines.xhtml
For peer review only

Proefpersoneninformatie voor deelname aan medisch-wetenschappelijk onderzoek

Cortisol om psychische bijwerkingen van dexamethason te voorkomen

Hydrocortison als co-medicatie om nadelige neuropsychiatrische effecten van dexamethason te voorkomen.

Inleiding

Geachte heer/mevrouw,

Wij vragen u om mee te doen aan een medisch-wetenschappelijk onderzoek.
Meedoen is vrijwillig. Om mee te doen is wel uw schriftelijke toestemming nodig. U ontvangt deze brief omdat bij u een hersentumor is gevonden waaraan u binnenkort wordt geopereerd. Voordat u beslist of u wilt meedoen aan dit onderzoek, is het belangrijk om meer te weten over het onderzoek. Lees deze informatie rustig door. Bespreek het met uw partner, vrienden of familie. Heeft u na het lezen van de informatie nog vragen? Dan kunt u terecht bij de onderzoeker of de onafhankelijk deskundige, die aan het eind van deze brief genoemd wordt. Verdere informatie over meedoen aan zo'n onderzoek staat in de bijgevoegde brochure 'Medisch-wetenschappelijk onderzoek'.

1. Algemene informatie

Dit onderzoek is opgezet door het Leids Universitair Medisch Centrum (LUMC) in samenwerking met het Erasmus MC Universitair Medisch Centrum Rotterdam (EMC), het Universitair Medisch Centrum Utrecht (UMCU) en Haaglanden Medisch Centrum (HMC). Voor dit onderzoek zijn 180 proefpersonen nodig. De medisch-ethische toetsingscommissie van het LUMC heeft dit onderzoek goedgekeurd. Algemene informatie over de toetsing van onderzoek vindt u in de brochure 'Medisch-wetenschappelijk onderzoek'.

2. Doel van het onderzoek

Het doel van dit onderzoek is uitzoeken of de psychische (geestelijke) bijwerkingen van het medicijn dexamethason verminderd kunnen worden. Dexamethason wordt standaard gebruikt bij hersenoperaties om zwelling en ontsteking te voorkomen. Echter, het is bekend dat dexamethason psychische bijwerkingen kan veroorzaken, bijvoorbeeld depressie, angst en verstoorde gedachten. Wij denken dat deze bijwerkingen worden veroorzaakt omdat dexamethason de aanmaak van het lichaamseigen hormoon cortisol (= hormoon gemaakt in de bijnier) afremt. Door nu dit lichaamseigen hormoon cortisol (= hydrocortison) in tabletvorm aan de dexamethason toe te voegen, denken wij dat deze bijwerkingen minder vaak zullen optreden.

For peer review only - http://bmjopen.bmj.com/site/about/guidelines.xhtml

3. Achtergrond van het onderzoek

Dexamethason wordt door dokters aan verschillende groepen patiënten voorgeschreven. Ongeveer 1 op de 5 patiënten krijgt last van psychische bijwerkingen of slaapproblemen. Of deze klachten daadwerkelijk optreden en de ernst hiervan hangen af van de dosis en de duur van de behandeling. Omdat deze klachten zeer hinderlijk kunnen zijn en kunnen leiden tot een verminderde kwaliteit van leven, willen wij met deze studie een behandeling testen om dit soort bijwerkingen tegen te gaan. Meer gedetailleerde informatie over het onderzoek kunt u vinden in **bijlage D:** Extra achtergrond informatie.

4. Wat meedoen inhoudt

Als u meedoet, krijgt u rondom de operatie, tijdens het verblijf in het ziekenhuis twee keer daags een extra tablet toegediend naast de normale behandeling. Deze tablet bevat ofwel het lichaamseigen hormoon hydrocortison, dat bij u niet meer aangemaakt wordt, ofwel een niet werkzame stof (placebo). Tijdens de opname en tot 3 maanden erna wordt u op verschillende momenten onderzocht waarbij u vragenlijsten moet invullen die de aanwezigheid van de psychische bijwerkingen en uw algehele functioneren in kaart brengen. De testmomenten voor het onderzoek worden gekoppeld aan de zorg die u al krijgt. De studiemedicatie zult u gedurende uw opname in het ziekenhuis krijgen en misschien thuis, dit zal maximaal 15 dagen zijn, afhankelijk van hoe lang u de dexamethason moet gebruiken. In de weken na uw ontslag uit het ziekenhuis zullen de vragenlijsten aan u voorgelegd worden op de dag dat u voor controle terug verwacht wordt in het ziekenhuis. Daarnaast moet u nog op 2 andere momenten thuis vragenlijsten invullen. Omdat het laatste meetmoment 3 maanden na de operatie is gepland, is de totale duur van de studie 3 maanden. U zult in totaal ongeveer 5 tot 6 uur met het invullen van de vragenlijsten bezig zijn verspreid over 3 maanden.

Geschiktheidsonderzoek

Eerst bepalen we of u kunt meedoen. Dit gebeurt aan de hand van een aantal voorwaarden waaraan u moet voldoen, daarbij zijn onder andere uw leeftijd en het gebruik van andere medicijnen van belang. Wanneer u besluit mee te doen aan de studie, wordt aan u gevraagd of u een toestemmingsformulier wilt tekenen. Na ondertekening wordt uw deelname aan de studie officieel. De onderzoeker zal dan vragen naar uw medische geschiedenis, zoals psychische achtergrond, medicatie en klachten.

Behandeling

We behandelen u met de studiemedicatie even lang als u volgens de standaard zorg het medicijn dexamethason toegediend krijgt. De ene helft van de mensen die deelnemen aan dit onderzoek krijgt het lichaamseigen hormoon hydrocortison, de andere helft krijgt een tablet waarin geen werkzame stof zit (placebo). Loting bepaalt welke behandeling u krijgt. U, de onderzoeker, verpleegkundige en de arts weten niet in welke groep u zit. Als het voor uw gezondheid belangrijk is, kan dit wel worden opgezocht. Algemene informatie hierover vindt u in de brochure ‘Medisch-wetenschappelijk onderzoek’.

For peer review only - http://bmjopen.bmj.com/site/about/guidelines.xhtml

Bezoeken en metingen

Voor deze studie zijn geen extra bezoeken aan het ziekenhuis nodig. De testen die uitgevoerd moeten worden voor het onderzoek, worden gepland op de dagen dat u toch al in het ziekenhuis bent.

Er zal dan het volgende gebeuren:

- Bij het eerste gesprek vragen we naar uw medische geschiedenis (psychisch en medicatie) en vragen naar klachten.
- We laten u op verschillende momenten vragenlijsten invullen over uw slaapkwaliteit, kwaliteit van leven, hoe u zichzelf voelt en uw psychologisch welzijn.
- We meten op verschillende momenten uw psychisch welzijn via een interview met één van de onderzoekers. Ook zullen de verpleegkundigen uw welzijn in de gaten houden.
- We laten u verschillende korte testen voor geheugen, aandacht en taal uitvoeren.
- We laten u een horloge dragen dat de slaapkwaliteit kan meten.

Veel van de vragenlijsten kunt u (digitaal) thuis invullen. In **bijlage C:** Schema onderzoek handelingen staat welke metingen er bij elk van de bezoeken plaatsvinden. En hoeveel tijd dit ongeveer in beslag zal nemen.

Tijdens het onderzoek kan aan u gevraagd worden om bloed af te staan. In dit bloed willen wij kijken naar genetische variatie. Daarmee onderzoeken we of de reactie op medicatie mede bepaald wordt door de samenstelling van uw DNA.

Daarnaast kan tijdens het onderzoek gevraagd worden of er een stukje van het verwijderde tumor- of hersenweefsel bewaard mag blijven voor nader onderzoek. In dit weefsel willen wij de werking van de hydrocortison onderzoeken.

Het buisje bloed en stukje van het verwijderde weefsel wordt eerst opgeslagen in de lokale onderzoeksinstelling en pas onderzocht nadat de studie is afgerond. Onderzoek naar het lichaamsmateriaal wordt in het LUMC uitgevoerd. Indien u niet in het desbetreffende centrum onder behandeling bent geweest, wordt uw lichaamsmateriaal naar het LUMC gebracht, opgeslagen en onderzocht. Meer gedetailleerde informatie over dit deel van het onderzoek kunt u vinden in **bijlage D:** Extra achtergrond informatie. Als u liever niet aan dit deel van het onderzoek wilt meedoen, kunt u dit aangeven in het toestemmingsformulier.

5. Wat wordt er van u verwacht

Om het onderzoek goed te laten verlopen is het belangrijk dat u zich aan de volgende afspraken houdt.

De afspraken zijn dat u:

- de studiemedicatie inneemt volgens de uitleg.
- niet meedoet aan nog een ander medisch-wetenschappelijk onderzoek.
- afspraken voor bezoeken nakomt.

For peer review only - http://bmjopen.bmj.com/site/about/guidelines.xhtml

Het is belangrijk dat u contact opneemt met de onderzoeker:

- voordat u andere geneesmiddelen gaat gebruiken. Ook als dat homeopathische geneesmiddelen, natuurgeneesmiddelen, vitaminen en/of geneesmiddelen van de drogist zijn.
- als u in de maanden na de operatie in een ziekenhuis wordt opgenomen of behandeld.
- als u plotseling gezondheidsklachten krijgt.
- als u niet meer wilt meedoen aan het onderzoek.
- als uw contactgegevens wijzigen.

Partner, familielid of andere naaste die voor u zorgt

Wij willen voor het onderzoek ook uw partner (of familielid of andere naaste) vragen om drie keer een vragenlijst in te vullen over uw psychisch welzijn.

6. Mogelijke bijwerkingen

Wij kunnen van te voren niet voorspellen of u last gaat krijgen van de dexamethason. Wij verwachten geen extra bijwerkingen door de studiemedicatie, omdat de studiemedicatie nagenoeg gelijk is aan de hoeveelheid lichaamseigen cortisol die normaal geproduceerd zou worden. We vullen een tekort aan. Meedoen aan de studie zal daarom naar onze inschatting geen extra risico's met zich meebrengen naast de al bestaande risico's van uw behandeling.

7. Mogelijke voor- en nadelen

Het is belangrijk dat u de mogelijke voor- en nadelen goed afweegt voordat u besluit mee te doen.

Voordelen van deelname:

- De studiemedicatie zou de mogelijke psychische bijwerkingen van dexamethason kunnen verminderen. Vooral gevoelens van angst en onrust zouden minder kunnen optreden, maar zeker is dat niet.
- De studiemedicatie zou uw slaapkwaliteit kunnen verbeteren, maar zeker is dat niet.

Nadelen van deelname:

- Het is belangrijk dat u de medicijnen met regelmaat en volgens de instructies inneemt.
- De testmomenten waarbij vragenlijsten ingevuld moeten worden kosten tijd en kunnen vermoeiend zijn.
- U heeft afspraken waaraan u zich moet houden.
- De vragenlijsten kunnen confronterend zijn, omdat ze vragen naar hoe u zich voelt en hoe het in het dagelijks leven met u gaat.
- Tijdens de testen voor het geheugen, de aandacht en de taal komen uw sterke maar ook uw minder sterke functies aan het licht. Dit kan soms confronterend zijn.

For peer review only - http://bmjopen.bmj.com/site/about/guidelines.xhtml

8. Als u niet wilt meedoen of wilt stoppen met het onderzoek

U beslist zelf of u meedoet aan het onderzoek. Deelname is vrijwillig. Als u niet wilt meedoen, hoeft u verder niets te doen. U hoeft niets te tekenen. U hoeft ook niet te zeggen waarom u niet wilt meedoen. U krijgt gewoon de behandeling die u anders ook zou krijgen. Er verandert niets.

Als u wel meedoet, kunt u zich altijd bedenken en toch stoppen, ook tijdens het onderzoek. U wordt dan weer op de gebruikelijke manier verder behandeld. U hoeft niet te zeggen waarom u stopt. Wel moet u dit direct melden aan de onderzoeker.
De gegevens die tot dat moment zijn verzameld, worden gebruikt voor het onderzoek. Als u wilt, en indien van toepassing, kan verzameld lichaamsmateriaal worden vernietigd. Dit moet u wel zelf aangeven. Wanneer u dit niet meldt, wordt het gebruikt voor het onderzoek.

Als er nieuwe informatie over het onderzoek is die belangrijk voor u is, laat de onderzoeker dit aan u weten. U wordt dan gevraagd of u blijft meedoen.

9. Einde van het onderzoek

Uw deelname aan het onderzoek stopt als

- alle testmomenten voorbij zijn en de vragenlijsten zijn ingeleverd
- u zelf kiest om te stoppen
- de arts het beter voor u vindt om te stoppen
- de overheid of de beoordelende medisch-ethische toetsingscommissie, besluit om het onderzoek te stoppen.

Het hele onderzoek is afgelopen als alle deelnemers klaar zijn.
De medicatie die u gebruikt heeft bij het onderzoek, is niet beschikbaar na afloop van het onderzoek.

Na het verwerken van alle gegevens informeert de onderzoeker u over de belangrijkste uitkomsten van het onderzoek. Dit gebeurt ongeveer 2 tot 3 jaar na uw deelname.
De onderzoeker kan u ook vertellen welke behandeling u heeft gehad/in welke groep u zat. Als u dit niet wilt, dan kunt u dit tegen de onderzoeker zeggen. De onderzoeker mag het u dan niet vertellen.

For peer review only - http://bmjopen.bmj.com/site/about/guidelines.xhtml

10. Gebruik en bewaren van uw gegevens en lichaamsmateriaal

Voor dit onderzoek worden uw persoonsgegevens en, indien van toepassing, lichaamsmateriaal verzameld, gebruikt en bewaard. Het gaat om gegevens zoals uw naam, adres, geboortedatum en om gegevens over uw gezondheid. Het verzamelen, gebruiken en bewaren van uw gegevens en mogelijk uw lichaamsmateriaal is nodig om de vragen die in dit onderzoek worden gesteld te kunnen beantwoorden en de resultaten te kunnen publiceren. Wij vragen voor het gebruik van uw gegevens en mogelijk lichaamsmateriaal uw toestemming.

Vertrouwelijkheid van uw gegevens en mogelijk lichaamsmateriaal

Om uw privacy te beschermen geven we een code aan uw gegevens en, indien van toepassing, uw lichaamsmateriaal. Uw naam en andere gegevens die u direct kunnen identificeren worden daarbij weggelaten. Alleen met de sleutel van de code zijn gegevens tot u te herleiden. De sleutel van de code blijft opgeborgen in de lokale onderzoeksinstelling. Ook in rapporten en publicaties over het onderzoek zijn de gegevens niet tot u te herleiden.

Toegang tot uw gegevens voor controle

Sommige personen kunnen op de onderzoekslocatie toegang krijgen tot al uw gegevens. Ook tot de gegevens zonder code. Dit is nodig om te kunnen controleren of het onderzoek goed en betrouwbaar is uitgevoerd. Personen die ter controle inzage kunnen krijgen in uw gegevens zijn: de commissie die de veiligheid van het onderzoek in de gaten houdt, monitors die voor het LUMC werken en de Inspectie Gezondheidszorg en Jeugd. Zij houden uw gegevens geheim. Wij vragen u voor deze inzage toestemming te geven.

Bewaartermijn gegevens en lichaamsmateriaal

Uw gegevens moeten 15 jaar worden bewaard op de onderzoekslocatie. Indien van toepassing, uw lichaamsmateriaal wordt voor onbepaalde tijd bewaard in de lokale onderzoeksinstelling. Hiermee willen wij na deze studie nog bepalingen doen. Onderzoek naar het lichaamsmateriaal wordt in het LUMC uitgevoerd. Indien u niet in het desbetreffende centrum onder behandeling bent geweest, wordt uw lichaamsmateriaal voor de onderzoeksbepalingen naar het LUMC gebracht, opgeslagen en onderzocht. De gegevens en lichaamsmateriaal die vanuit een deelnemend ziekenhuis naar het LUMC worden gestuurd bevatten alleen de code, en niet uw naam of andere gegevens waarmee u kunt worden geïdentificeerd.

For peer review only - http://bmjopen.bmj.com/site/about/guidelines.xhtml

Intrekken toestemming

U kunt uw toestemming voor gebruik van uw persoonsgegevens en mogelijk lichaamsmateriaal altijd weer intrekken. Dit geldt voor dit onderzoek en ook voor het bewaren en het gebruik voor het toekomstige onderzoek. De onderzoeksgegevens die zijn verzameld tot het moment dat u uw toestemming intrekt worden nog wel gebruikt in het onderzoek. Uw lichaamsmateriaal wordt alleen op uw verzoek vernietigd. Als er al metingen met dat lichaamsmateriaal zijn gedaan, dan worden die gegevens nog wel gebruikt.

Meer informatie over uw rechten bij verwerking van gegevens

Voor algemene informatie over uw rechten bij verwerking van uw persoonsgegevens kunt u de website van de Autoriteit Persoonsgegevens raadplegen.

Bij vragen over uw rechten kunt u contact opnemen met de verantwoordelijke voor de verwerking van uw persoonsgegevens. Voor dit onderzoek is dat: het LUMC. Zie **bijlage A:** Contactgegevens.

Bij vragen of klachten over de verwerking van uw persoonsgegevens raden we u aan eerst contact op te nemen met de onderzoekslocatie. U kunt contact opnemen met de Functionaris voor de Gegevensbescherming van de instelling of de Autoriteit Persoonsgegevens. Zie **bijlage A:** Contactgegevens.

Registratie van het onderzoek

Informatie over dit onderzoek is ook opgenomen in een overzicht van medisch-wetenschappelijke onderzoeken, namelijk in het Nederlands Trial Register (http://www.trialregister.nl). Daarin zijn geen gegevens opgenomen die naar u herleidbaar zijn. Na het onderzoek kan de website een samenvatting van de resultaten van dit onderzoek tonen. U vindt dit onderzoek onder de naam: DEXA-CORT.

11. Verzekering voor proefpersonen

Voor iedereen die meedoet aan dit onderzoek is een verzekering afgesloten. De verzekering dekt schade door het onderzoek. Niet alle schade is gedekt. In **bijlage B:** Informatie over de verzekering, vindt u meer informatie over de verzekering en de uitzonderingen. Daar staat ook aan wie u de schade kunt melden.

12. Informeren huisarts en behandelend specialist

Wij zullen uw huisarts niet informeren dat u meedoet aan het onderzoek. Als u dit liever wel wilt, kunt u altijd zelf contact opnemen met uw huisarts om te vertellen dat u aan de studie meedoet. Bij vragen over uw medische geschiedenis of medicijngebruik, nemen we wel contact op met uw huisarts of behandeld arts. U kunt niet deelnemen aan het onderzoek als u geen huisarts heeft. Uw behandelend arts wordt wel geïnformeerd over uw deelname via uw dossier.

For peer review only - http://bmjopen.bmj.com/site/about/guidelines.xhtml

13. Geen vergoeding voor meedoen

De studiemedicatie voor het onderzoek kost u niets. U wordt niet betaald voor het meedoen aan dit onderzoek. Studie afspraken worden gepland op dagen dat u al een afspraak in het ziekenhuis heeft. Wanneer u voor het onderzoek extra naar het ziekenhuis moet komen, zullen uw reiskosten worden vergoed.

14. Heeft u vragen?

Bij vragen kunt u contact opnemen met de onderzoeker of het onderzoeksteam. Voor onafhankelijk advies over meedoen aan dit onderzoek kunt u terecht bij de onafhankelijke arts; dr. Stijn W. Genders. Hij weet veel over het onderzoek, maar hij is niet betrokken bij deze studie. Indien u klachten heeft over het onderzoek, kunt u dit bespreken met de onderzoeker of uw behandelend arts. Wilt u dit liever niet, dan kunt u zich wenden tot de klachtenfunctionaris van uw ziekenhuis. Alle gegevens vindt u in **bijlage A**: Contactgegevens.

15. Ondertekening toestemmingsformulier

Wanneer u voldoende bedenktijd van minimaal 1 week en maximaal 2 weken heeft gehad, wordt u gevraagd te beslissen over deelname aan dit onderzoek. Indien u toestemming geeft, zullen wij u vragen deze op de bijbehorende toestemmingsverklaring schriftelijk te bevestigen. Door uw schriftelijke toestemming geeft u aan dat u de informatie heeft begrepen en instemt met deelname aan het onderzoek. Zowel uzelf als de onderzoeker ontvangen een getekende versie van deze toestemmingsverklaring.

Dank voor uw aandacht.

For peer review only

For peer review only - http://bmjopen.bmj.com/site/about/guidelines.xhtml

16. Bijlagen bij deze informatie

A. Contactgegevens

B. Informatie over de verzekering

C. Schema onderzoek handelingen

D. Extra achtergrond informatie

E. Toestemmingsformulier

F. Toestemmingsformulier voor partner (of familielid of andere naaste)

G. Brochure 'Medisch-wetenschappelijk onderzoek. Algemene informatie voor de proefpersoon'

For peer review only

For peer review only - http://bmjopen.bmj.com/site/about/guidelines.xhtml

Bijlage A: Contactgegevens

Coördinerend onderzoeker	Onafhankelijk arts
Anne-Sophie Koning, MSc	Dr. Stijn W. Genders
dexonderzoek@lumc.nl	oog.stafsecretariaat@lumc.nl
+31-71-5265303 / +31-71-5263082	+31-71-5262374
**LUMC contact gegevens**	
Dr. Wouter R. van Furth	
neurochirurgie@lumc.nl	
+31-71-5262109	
**Functionaris voor de Gegevensbescherming LUMC**	
Wanneer u vragen heeft over de bescherming van uw privacy kunt u contact opnemen de functionarissen gegevensbescherming van het LUMC (FG) via infoavg@lumc.nl	
**Klachtenfunctionaris van het LUMC**	
Bij klachten kunt u zich melden bij het patiëntenservicebureau in het LUMC, locatie H2-11 (routenummer 473, tegenover het Leidseplein). Hier kunt u melding maken van uw onvrede en het klachtenformulier invullen. Het patiëntenservicebureau informeert u zo snel mogelijk over een mogelijke oplossing en kan eventueel de klachtenfunctionaris inschakelen. U kunt ook het klachtenformulier digitaal invullen. Zie website LUMC pagina klacht indienen<br><br>**Contactgegevens patiëntenservicebureau**<br>LUMC Patiëntenservicebureau<br>Postbus 9600<br>2300 RC Leiden<br>Telefoon: +31 71-5262989	

For peer review only

For peer review only - http://bmjopen.bmj.com/site/about/guidelines.xhtml

Bijlage B: Informatie over de verzekering

Voor iedereen die meedoet aan dit onderzoek heeft het LUMC een verzekering afgesloten. De verzekering dekt schade door deelname aan het onderzoek. Dit geldt voor schade tijdens het onderzoek of binnen vier jaar na het einde van uw deelname aan het onderzoek. Schade moet u binnen die vier jaar aan de verzekeraar hebben gemeld.

De verzekering dekt niet alle schade. Onderaan deze tekst staat in het kort welke schade niet wordt gedekt.
Deze bepalingen staan in het Besluit verplichte verzekering bij medisch-wetenschappelijk onderzoek met mensen. Dit besluit staat op www.ccmo.nl, de website van de Centrale Commissie Mensgebonden Onderzoek (zie 'Bibliotheek' en dan 'Wet- en regelgeving').

Bij schade kunt u direct contact leggen met de verzekeraar.

De verzekeraar van het onderzoek is:	
Naam:	Centramed
Adres:	Maria Montessorilaan 9, 2719 DB Zoetermeer
Telefoonnummer:	070-3017070
E-mail:	info@centramed.nl
Polisnummer:	624.530.305

De verzekering biedt een dekking van € 650.000,- per proefpersoon en € 5.000.000,- voor het hele onderzoek (en € 7.500.000,- per jaar voor alle onderzoeken van het Leids Universitair Medisch Centrum).

De verzekering dekt de volgende schade **niet**:

- schade door een risico waarover u in de schriftelijke informatie bent ingelicht. Dit geldt niet als het risico zich ernstiger voordoet dan was voorzien of als het risico heel onwaarschijnlijk was;
- schade aan uw gezondheid die ook zou zijn ontstaan als u niet aan het onderzoek had meegedaan;
- schade door het niet (volledig) opvolgen van aanwijzingen of instructies;
- schade aan uw nakomelingen, als gevolg van een negatief effect van het onderzoek op u of uw nakomelingen;
- schade door een bestaande behandelmethode bij onderzoek naar bestaande behandelmethoden.

For peer review only - http://bmjopen.bmj.com/site/about/guidelines.xhtml

Bijlage C: Schema onderzoek handelingen

Voor deze studie zijn geen extra bezoeken aan het ziekenhuis nodig. De testen die uitgevoerd moeten worden voor het onderzoek, worden gepland op de dagen dat u in het ziekenhuis bent.

Poli bezoek

Volgens reguliere zorg wordt er een poli afspraak gepland met de neurochirurg. Op de dag van deze poli afspraak willen wij u vragen 1 vragenlijst in te vullen, 1 interview af te laten nemen en een aantal testen voor het geheugen, de aandacht en de taal uit te voeren. Indien mogelijk, ook 1 vragenlijst voor uw partner (of familielid of andere naaste). Bij patiënten met een linkszijdige hersentumor willen wij nog een extra taalscreening doen. In totaal zal dit studiemoment ongeveer 80 (of 70) minuten duren.

Verder zullen wij via de email 5 vragenlijsten naar u sturen, die u thuis kunt invullen. Het invullen van alle vragenlijsten zal in totaal ongeveer 45 minuten in beslag nemen.

Dagen op verpleegafdeling

Op de dag van opname en op de dag van de operatie hoeft u niks te doen. Na de operatie zult u een aantal dagen in het ziekenhuis verblijven volgens reguliere zorg. Het aantal dagen dat u in het ziekenhuis ligt is afhankelijk van het oordeel van de artsen. Dit wordt volgens reguliere zorg bepaald. Op de dagen dat u op de verpleegafdeling verblijft, vragen wij u steeds 1 korte vragenlijst in te vullen. Ook vragen wij u een horloge te dragen waarmee we uw activiteit en slaap kwaliteit kunnen meten. Daarnaast willen wij u vragen in een slaapdagboek kort aan te geven hoe u geslapen heeft. De tijd die u deze dagen in het ziekenhuis kwijt bent aan het onderzoek is maximaal 10 minuten. Gedurende uw opname in het ziekenhuis zullen verpleegkundigen u psychisch welzijn in de gaten houden.

Dag van ontslag

Op de dag dat u naar huis mag, willen wij een interview bij u afnemen, vragen wij u 2 vragenlijsten in te vullen en we zullen een korte screening voor het geheugen, de aandacht en de taal uitvoeren. Ook willen wij uw partner (of familielid of andere naaste) vragen 1 vragenlijst in te vullen. Bij patiënten met een linkszijdige hersentumor willen wij nog een extra taalscreening doen. Dit studiemoment zal ongeveer 55 (of 45) minuten duren.

Na operatie

Ongeveer 2 weken na de operatie willen wij u vragen 4 vragenlijsten in te vullen. Dit kunt u gewoon thuis doen en het invullen van alle vragenlijsten zal in totaal ongeveer 30 minuten duren.

Rond 5 tot 8 weken na de operatie, wordt er volgens reguliere zorg een afspraak in het ziekenhuis gepland. Op deze dag in het ziekenhuis willen wij nog 1 keer het interview bij u afnemen, vragen wij u nog 2 vragenlijsten in te vullen en een aantal testen voor het geheugen, de aandacht en de taal uit te voeren. Indien mogelijk, ook 1 vragenlijst voor uw partner (of familielid of andere naaste). Dit studiemoment zal ongeveer 80 minuten duren.

For peer review only - http://bmjopen.bmj.com/site/about/guidelines.xhtml

Ongeveer 3 maanden na operatie willen wij u vragen 5 vragenlijsten in te vullen. Dit kunt u gewoon thuis doen en het invullen van alle vragenlijsten zal in totaal ongeveer 45 minuten duren.

In totaal zal de studie ongeveer 5 tot 6 uur van u tijd kosten verspreid over 3 maanden.

**Studiemoment**	**Waar**	**Hoe lang**
Preoperatief	Poli bezoek	70 (tot 80) minuten
	Thuis	45 minuten
Gedurende ziekenhuisopname	Op de afdeling	30 minuten
Dag van ontslag	Op de afdeling	45 (tot 55) minuten
2 weken postoperatief	Thuis	30 minuten
5 – 8 weken postoperatief	Ziekenhuis	80 minuten
3 maanden postoperatief	Thuis	45 minuten
**Totaal**		**5 – 6 uur**

For peer review only - http://bmjopen.bmj.com/site/about/guidelines.xhtml

Bijlage D – Extra achtergrond informatie

Ons lichaam maakt het hormoon cortisol. Cortisol beïnvloedt veel dingen, zoals onze stemming en onze slaap. Cortisol wordt gemaakt in de bijnieren, die aangezet worden door een ander hormoon adrenocorticotroop hormoon (ACTH). Dat ACTH komt uit de hypofyse, een orgaan onder de hersenen. Als er genoeg cortisol is, krijgt de hypofyse een seintje om te stoppen met het maken van hormonen, waardoor de bijnieren dan ook geen cortisol meer maken.

Het medicijn dexamethason lijkt heel erg veel op cortisol dat het lichaam zelf kan maken. Maar het is veel sterker dan het lichaamseigen cortisol. Een kleine dosis dexamethason kan er al voor zorgen dat de hypofyse geen hormoon meer maakt. Daardoor stoppen de bijnieren met cortisol maken. Dus een bijwerking bij behandeling met dexamethason is dat ons eigen lichaam geen cortisol meer maakt.

Er is één groot verschil tussen cortisol en dexamethason. Dexamethason werkt via één aangrijpingspunt. Dat is via de glucocorticoïd receptor (GR). Cortisol werkt op twéé aangrijpingspunten: via de GR, maar ook via de mineralocorticoïd receptor (MR). Cortisol activeert dus MR en GR. Dexamethason activeert alleen de GR. Omdat ons lichaam na dexamethason geen cortisol meer maakt, kan cortisol ook niet meer de MR activeren. Het resultaat is dat alleen de GR actief is, terwijl de MR leeg blijft. Wij denken dat de bijwerkingen na dexamethason komen doordat de MR leeg is, omdat de werking van cortisol in de hersenen van de MR afhangt.

In deze studie voegen we cortisol toe aan de behandeling met dexamethason (als pilletje heeft cortisol de naam 'hydrocortison' gekregen). De hydrocortison kan binden aan de MR. Op deze manier hopen wij de normale werking van de MR weer terug te krijgen. En hopen we dat de bijwerkingen zullen verminderen.

Tijdens het onderzoek kan gevraagd worden of er bloed van u mag worden afgenomen en bewaard mag worden voor verder onderzoek. In het bloed willen wij kijken welk type van MR u heeft. Dit kunnen wij onderzoeken door in het DNA naar het gen van de MR te kijken. Het is namelijk bekend dat een bepaalde variant van het MR gen een lager risico geeft op het krijgen van depressie. Wij willen in dit onderzoek kijken of dit een onderliggende reden kan zijn voor de klachten die sommige patiënten van de dexamethason krijgen.

Met het weefsel dat van het verwijderde tumorweefsel gehaald wordt, willen wij de werking van de hydrocortison onderzoeken. Met de toegevoegde hydrocortison verwachten wij dat de MR weer actief wordt. Dat willen wij in dit weefsel meten.

For peer review only

For peer review only - http://bmjopen.bmj.com/site/about/guidelines.xhtml

Bijlage E: Toestemmingsformulier proefpersoon

Cortisol om psychische bijwerkingen van dexamethason te voorkomen

- Ik heb de informatiebrief gelezen. Ook kon ik vragen stellen. Mijn vragen zijn voldoende beantwoord. Ik had genoeg tijd om te beslissen of ik meedoe.

- Ik weet dat meedoen vrijwillig is. Ook weet ik dat ik op ieder moment kan beslissen om toch niet mee te doen of te stoppen met het onderzoek. Daarvoor hoef ik geen reden te geven.

- Ik geef toestemming voor het opvragen van informatie bij mijn huisarts/specialist(en) die mij behandelt indien er vragen zijn over uw medische geschiedenis of medicijngebruik.

- Ik weet dat voor de controle van het onderzoek sommige mensen toegang tot al mijn gegevens kunnen krijgen. Die mensen staan vermeld in deze informatiebrief. Ik geef toestemming voor die inzage door deze personen.

- Ik weet dat mijn partner (of familielid of andere naaste) ook een aantal keer een vragenlijst moet invullen en hij/zij gaat hiermee akkoord.

- Ik geef toestemming voor het verzamelen en gebruiken van mijn gegevens voor de beantwoording van de onderzoeksvraag in dit onderzoek.

- Ik geef ◻ **wel**
  ◻ **geen**
  toestemming voor bloedafname en dit te bewaren en te gebruiken voor toekomstig onderzoek op het gebied van onderzoek beschreven in de informatiebrief.

- Ik geef ◻ **wel**
  ◻ **geen**
  toestemming voor het bewaren van een stukje weefsel van het verwijderde tumorweefsel en dit te bewaren en te gebruiken voor toekomstig onderzoek op het gebied van onderzoek beschreven in de informatiebrief.

For peer review only

For peer review only - http://bmjopen.bmj.com/site/about/guidelines.xhtml

Versie 4, 14-02-20

- Ik geef ☐ **wel**
  ☐ **geen**
  toestemming om mijn persoonsgegevens en lichaamsmateriaal langer te bewaren en te gebruiken voor toekomstig onderzoek op het gebied van onderzoek beschreven in de informatiebrief.

- Ik wil ☐ **wel**
  ☐ **niet**
  geïnformeerd worden over welke behandeling ik heb gehad/in welke groep ik zat.

- Ik wil meedoen aan dit onderzoek.

Naam proefpersoon: ___________________

Handtekening:________________________ Datum : __ / __ / __

Ik verklaar dat ik deze proefpersoon volledig heb geïnformeerd over het genoemde onderzoek.

Als er tijdens het onderzoek informatie bekend wordt die de toestemming van de proefpersoon zou kunnen beïnvloeden, dan breng ik hem/haar daarvan tijdig op de hoogte.

Naam onderzoeker (of diens vertegenwoordiger):_______________________________

Handtekening:_______________________ Datum: __ / __ / __

For peer review only - http://bmjopen.bmj.com/site/about/guidelines.xhtml

Bijlage F: Toestemmingsformulier voor partner (of familielid of andere naaste)

Cortisol om psychische bijwerkingen van dexamethason te voorkomen

- Ik ben op de hoogte dat mijn partner (of familielid of andere naaste) meedoet met het onderzoek. Ook kon ik vragen stellen. Mijn vragen zijn voldoende beantwoord. Ik had genoeg tijd om te beslissen of ik voor de studie 3x een vragenlijst wil invullen over mijn partner (of familielid of andere naaste).

- Ik weet dat meedoen vrijwillig is. Ook weet ik dat ik op ieder moment kan beslissen om toch niet mee te doen of te stoppen met het onderzoek. Daarvoor hoef ik geen reden te geven.

- Ik wil meedoen aan dit onderzoek.

Naam partner (of familielid of andere naaste):______________

Handtekening:_______________________ Datum : __ / __ / __

Ik verklaar dat ik de partner (of familielid of andere naaste) volledig heb geïnformeerd over het genoemde onderzoek.

Als er tijdens het onderzoek informatie bekend wordt die de toestemming van de partner (of familielid of andere naaste) zou kunnen beïnvloeden, dan breng ik hem/haar daarvan tijdig op de hoogte.

Naam onderzoeker (of diens vertegenwoordiger):______________________________

Handtekening:______________________ Datum: __ / __ / __

For peer review only - http://bmjopen.bmj.com/site/about/guidelines.xhtml

The

DEXA-CORT

Trial

For peer review only

For peer review only - http://bmjopen.bmj.com/site/about/guidelines.xhtml

Study information for participation in medical research

Cortisol to prevent psychiatric adverse effects of dexamethasone

Hydrocortisone as co-medication to prevent adverse neuropsychiatric effects of dexamethasone

Introduction

Dear Sir/Madam,

You are kindly requested to take part in a medical scientific study. We have contacted you because you will undergo surgery for a brain tumor. Participation is voluntarily and requires your written consent. Before you decide whether you want to participate in this study, you will be given an explanation about what the study involves. Please read this information carefully and discuss it with your partner, friends and/or family. If you have any questions you can contact the (coordinating) investigator or independent expert for an explanation. General information about participating in a study can be found in the enclosed general brochure on medical research.

1. General information

This research was set up by the Leiden University Medical Center (LUMC) in collaboration with the Erasmus MC University Medical Center Rotterdam (EMC), the University Medical Center Utrecht (UMCU) and Haaglanden Medical Center (HMC). 180 participants are needed for this study. The medical ethics review committee of the LUMC has approved this research. General information about the assessment of research can be found in the brochure 'Medical scientific research'.

2. Purpose of the study

The aim of this study is to find out whether the psychological (mental) side effects of the drug dexamethasone can be reduced. Dexamethasone is commonly used in brain surgery to prevent swelling and inflammation. However, it is known that dexamethasone can cause psychological side effects, for example depression, anxiety and disturbed thoughts. We think that these side effects are caused because dexamethasone inhibits the production of the body's own hormone cortisol (= hormone made in the adrenal gland). By now adding this body's hormone cortisol (= hydrocortisone) in tablet form to the dexamethasone, we think that these side effects will occur less often.

3. Background of the study

Dexamethasone is prescribed by doctors to different groups of patients. About 1 in 5 patients suffer from psychological side effects or sleeping problems. Whether these complaints actually

For peer review only - http://bmjopen.bmj.com/site/about/guidelines.xhtml

occur and their severity may depend on the dose and the duration of the treatment. Because these complaints can be very bothersome and can lead to a reduced quality of life, we want to use this study to test a treatment to counteract these kinds of side effects. More detailed information about the study can be found in **Appendix** D: Additional background information.

4. What does participation involve?

If you participate, you will be given an extra tablet twice a day in addition to the normal treatment during the hospital stay. This tablet contains either the body's own hormone hydrocortisone, which you no longer produce yourself, or an inactive substance (placebo). During the admission and up to 3 months afterwards, you will be examined at various times during which you have to fill in questionnaires that map the presence of the psychological side effects and your overall functioning. The test moments for the study are coinciding with standard appointments at the hospital. You will receive the study medication during your hospital stay and perhaps at home, this will be up to 15 days, depending on how long you need to take the dexamethasone. In the weeks following your discharge from hospital, the questionnaires will be presented to you on the day you are expected to return to the hospital for a check-up. In addition, you have to complete questionnaires at home at 2 other times. Because the last measurement moment is planned 3 months after surgery, the total duration of the study is 3 months. You will spend a total of about 5 to 6 hours filling in the questionnaires spread over 3 months.

Screening

If you are interested in participating we first will evaluate whether you may participate. This is determined on the basis of a number of conditions that you must meet, including your age and the use of other medicines. When you decide to participate in the study, you will be asked to sign a consent form. After signing, your participation in the study becomes official. The researcher will then ask about your medical history, such as psychological background, medication and complaints.

Treatment

We will treat you with the study medication for as long as you are administered the drug dexamethasone according to standard care. Half of the people participating in this study will be given the body's own hormone hydrocortisone, the other half will be given a tablet that does not contain an active ingredient (placebo). The treatment you will receive is determined by draw. You, the researcher, nurse and doctor do not know which group you are in. If it is important for your health, this can be looked up. General information about this can be found in the brochure 'Medical-scientific research'.

Visits and measurements

No additional hospital visits are required for this study. The tests to be performed for the examination are scheduled on the days that you are already in the hospital.

The following will then happen:

For peer review only - http://bmjopen.bmj.com/site/about/guidelines.xhtml

- At the first meeting we ask about your medical history (psychological and medication) and complaints.
- We let you fill in questionnaires at various times about your sleep quality, quality of life, how you feel and your psychological well-being.
- We measure your psychological well-being at various times through an interview with one of the researchers. The nurses will also monitor your well-being.
- We let you perform several short tests for memory, attention and language.
- We let you wear a watch that can measure sleep quality.

You can complete many of the questionnaires (digitally) at home. **Appendix C**: Schedule of investigation actions, lists the measurements that take place during each of the visits. And how long this will approximately take.

During the study, you may be asked to donate a tube of blood. In this blood sample we want to look at genetic variation. We use this to investigate whether the response to medication is partly determined by the composition of your DNA.

In addition, you may be asked whether a piece of the removed tumor tissue may be kept for further examination. We want to investigate the effect of the hydrocortisone in this tissue.

The samples of blood and/or tissue are first stored at the local study facility and examined only after the study is completed. Research on the body material is carried out in the LUMC. If you have not been treated in this center, your body material will be taken to the LUMC, stored and examined. More detailed information about this part of the study can be found in **Appendix D**: Additional background information. If you prefer not to participate in this part of the study, you can indicate this in the consent form.

5. What is expected of you?

In order for the study to run smoothly, it is important that you adhere to the following agreements.

The agreements are that you:

- Take the study medication according to the instructions.
- Do not participate in any other medical scientific research.
- Fulfill appointments for study visits.

It is important that you contact the researcher:

- before taking any other medicines. Even if they are homeopathic medicines, natural medicines, vitamins and/or medicines from the drugstore.
- if you are hospitalized or treated in the months following the operation.
- if you suddenly develop health problems.
- if you no longer wish to participate in the study.
- if your contact details change.

For peer review only - http://bmjopen.bmj.com/site/about/guidelines.xhtml

Partner, family member or other close relative

Before the study, we would also like to ask your partner (or family member or other close relative) to complete a questionnaire about your psychiatric well-being three times.

6. Possible side effects

We cannot predict in advance whether you will suffer from the dexamethasone. We do not expect any additional side effects from the study medication, because the study medication is almost equal to the amount of the body's own cortisol that would normally be produced. We refill a shortage. In our opinion, participating in the study will therefore not entail any additional risks in addition to the already existing risks of your treatment.

7. Possible advantages and disadvantages

It is important that you carefully consider the possible advantages and disadvantages before you decide to participate.

Benefits of participating:

- The study medication could reduce the possible psychiatric side effects of dexamethasone. Especially feelings of fear and restlessness could occur less, but this is not certain.
- The study medication could improve your sleep quality, but this is not certain.

Disadvantages of participating:

- It is important that you take the medicines regularly and according to the instructions.
- The test moments where questionnaires have to be completed take time and can be tiring.
- You have appointment that you must fulfill.
- The questionnaires can be confronting, because they ask about how you feel and how you are doing in daily life.
- Memory, attention and language tests will reveal your strengths and weaknesses. This can sometimes be confronting.

8. If you do not wish to participate or wish to stop participating

It is up to you to decide whether or not to participate in the study. Participation is voluntary. If you do not want to participate, you will continue your usual treatment as before. You do not have to sign anything and you do not have to explain why you do not want to participate.

If you do participate in the study, you can always change your mind. You may stop participation at any time during the study. This is not harmful and will not affect your usual treatment. You do not have to say why you are stopping, but you do need to tell the investigator immediately. The data collected until that time can still be used for the study.

For peer review only - http://bmjopen.bmj.com/site/about/guidelines.xhtml

If you wish, and if applicable, collected body material can be destroyed. You must indicate this yourself. If you do not report this, it will be used for the investigation.

If there is new information about the study that is important to you, the researcher will let you know. You will then be asked if you want to continue participating.

9. End of the study

Your participation in the study will end if:

- all test moments are done and the questionnaires have been submitted
- you choose to stop yourself
- the doctor thinks it's better for you to stop
- the government or the assessing medical-ethical review committee, decides to stop the research.

The entire study ends when all participants completed the study.
The medication you took during the study will not be available after the study.

After processing all the data, the researcher will inform you about the most important results of the study. This happens approximately 2 to 3 years after your participation.
The researcher can also tell you what treatment you received. If you do not want this, you can say so to the researcher. The researcher is not allowed to tell you.

10. Use and storage of your data and body material

For this research, your personal data and, if applicable, body material will be collected, used and stored. This concerns data such as your name, address, date of birth and data about your health. The collection, use and storage of your data and possibly your body material is necessary to answer the questions posed in this study and to publish the results. We ask your permission for the use of your data and possible body material.

Confidentiality of your data and possible body material

To protect your privacy, we give a code to your data and, if applicable, your body material. Your name and other information that can directly identify you are omitted. Data can only be traced back to you with the key of the code. The key to the code remains stored in the local research facility. Also in reports and publications about the research, the data cannot be traced back to you.

Access your data

Some individuals may have access to all of your data at the study site. Also to the data without code. This is necessary to be able to check whether the research has been carried out properly and reliably. Persons who can access your data for inspection are: the committee that monitors

For peer review only

For peer review only - http://bmjopen.bmj.com/site/about/guidelines.xhtml

the safety of the research, a monitor who works for the LUMC and the Health and Youth Care Inspectorate. They keep your details secret. We ask you to give permission for this inspection.

Storing period of the data and body material

Your data must be kept at the study location for 15 years. If applicable, your body material will be stored indefinitely at the local research facility. We intend further research with these materials after this study. Research into the body material is carried out in the LUMC. If you have not been treated in the center in question, your body material will be brought to the LUMC for the examination, storage and examination. The data and body material sent to the LUMC from a participating hospital only contain the code, and not your name or other data with which you can be identified.

Withdraw permission

You can always withdraw your consent for the use of your personal data and possible body material. This applies to this research and also to the storage and use for future research. The research data collected up to the moment you withdraw your consent will still be used in the research. Your body material will only be destroyed at your request. If measurements have already been taken with the body material, those data will still be used.

More information about your rights when processing data

For general information about your rights when processing your personal data, you can consult the website of the Dutch Data Protection Authority.

If you have any questions about your rights, please contact the person responsible for processing your personal data. For this study, that is: the LUMC. See **Appendix A**: Contact Details.

If you have any questions or complaints about the processing of your personal data, we recommend that you first contact the research location. You can contact the Data Protection Officer of the institution or the Dutch Data Protection Authority. See **Appendix A**: Contact Details.

Registration of the study

Information about this study is also included in an overview of medical scientific studies, namely in the Dutch Trial Register (http://www.trialregister.nl). It does not contain any data that can be traced back to you. After finishing the study, the website may display a summary of the results of this survey. You will find this study under the name: DEXA-CORT.

For peer review only - http://bmjopen.bmj.com/site/about/guidelines.xhtml

11. Insurance for study participants

Insurance has been taken out for everyone participating in this study. The insurance covers damage caused by the study. Not all damage is covered. In **Appendix B**: Information about the insurance, you will find more information about the insurance and the exceptions. It also states who you can report the damage to.

12. Informing the general practitioner and treating specialist

We will not inform your GP that you are participating in the study. If you prefer, you can always contact your GP yourself to let them know that you are participating in the study. If we have any questions about your medical history or medication use, we will contact your GP or treating physician. You cannot participate in the study if you do not have a GP. Your treating physician will be informed about your participation.

13. No compensation for participating

The study medication for the study are free of charge for you. You will not be paid for participating in this study. Study appointments are scheduled on days when you already have a hospital appointment. If we cannot schedule coincide appointments and you have to visit the hospital only for study appointments, you will be reimbursed for your travel costs.

14. Any questions?

If you have any questions, please contact the researcher or the research team. For independent advice about participating in this study, you can contact the independent doctor; Dr. Stijn W. Genders. He knows a lot about the research, but he is not involved in this study. If you have any complaints about the study, you can discuss this with the researcher or your attending physician. If you prefer not to do this, you can contact the complaints officer of your hospital. All details can be found in **Appendix A**: Contact details.

15. Signing the consent

You do not have to decide right away whether you want to participate in the study. When you have had sufficient reflection time of at least 1 week and a maximum of 2 weeks, you will be asked to decide whether to participate in this study. If you give permission, we will ask you to confirm this in writing on the accompanying statement of consent. By your written consent, you indicate that you have understood the information and agree to participate in the study. Both you and the researcher will receive a signed version of this consent form.

Thank you for your attention.

For peer review only - http://bmjopen.bmj.com/site/about/guidelines.xhtml

16. Appendices

A. Contact details

B. Insurance information

C. Schedule of investigation actions

D. Additional background information

E. Informed Consent Form

F. Informed Consent Form (for family or other relative)

G. Brochure ‘Medical research. General information for research participants’

For peer review only

For peer review only - http://bmjopen.bmj.com/site/about/guidelines.xhtml

Appendix A: Contact details

**Coordinating investigator**	**Independent expert**
Anne-Sophie Koning, MSc	Dr. S.W. Genders
dexonderzoek@lumc.nl	oog.stafsecretariaat@lumc.nl
+31-71-5265303 / +31-71-5263082	+31-71-5262374
**LUMC contact details**	
Dr. W.R. van Furth	
neurochirurgie@lumc.nl	
+31-71-5262109	
**Data protection officers of the LUMC**	
If you have any questions about the protection of your privacy, you can contact the data protection officers of the LUMC (FG) via infoavg@lumc.nl	
**Complaints officer of the LUMC**	
In case of complaints, you can report to the patient service desk in the LUMC, location H2-11 (route number 473, opposite Leidseplein). Here you can report your dissatisfaction and fill in the complaint form. The patient service bureau will inform you as soon as possible about a possible solution and may call in the complaints officer if necessary. You can also fill in the complaint form digitally. See website LUMC page submit a complaint.<br><br>**Contact details patient service desk**<br>LUMC Patient service desk<br>Postbus 9600<br>2300 RC Leiden<br>Phone: +31 71 5262989	

For peer review only

For peer review only - http://bmjopen.bmj.com/site/about/guidelines.xhtml

Appendix B: Insurance information

The LUMC has taken out insurance for everyone who participates in this study. The insurance covers damage caused by participating in the study. This applies to damage during the study or within four years of the end of your participation in the study. You must have reported damage to the insurer within those four years.

The insurance does not cover all damage. At the bottom of this text is a brief description of which damage is not covered.
These provisions are contained in the Decree on compulsory insurance for medical research involving humans. This decision can be found on www.ccmo.nl, the website of the Central Committee for Research on Human Subjects (see 'Library' and then 'Laws and regulations').

In the event of damage, you can contact the insurer directly.

The insurance company is:	
Name:	Onderlinge Waarborgmaatschappij Centramed B.A.
Address:	Maria Montessorilaan 9, 2719 DB Zoetermeer
Phone:	070-3017070
Email:	info@centramed.nl
Policy number:	624.530.305

The insurance offers cover of € 650,000 per test participant and € 5,000,000 for the entire study (and € 7,500,000 per year for all studies of the Leiden University Medical Center).

The insurance does **not** cover the following damage::

- damage from a risk of which you have been informed in the written information. This does not apply if the risk is more serious than anticipated or if the risk was very unlikely;
- damage to your health that would also have occurred if you had not taken part in the study;
- damage caused by not (fully) following directions or instructions;
- damage to your descendants, as a result of a negative effect of the research on you or your descendants;
- damage caused by an existing treatment method when researching existing treatment methods.

For peer review only - http://bmjopen.bmj.com/site/about/guidelines.xhtml

Appendix C: Schedule of investigation actions

No additional hospital visits are required for this study. The examinations to be performed are scheduled on the days you are in the hospital.

Outpatient clinic visit

According to regular care, an outpatient clinic appointment is scheduled with the neurosurgeon. On the day of this outpatient clinic appointment, we would like to ask you to fill in 1 questionnaire, to have 1 interview and to perform a number of tests for memory, attention and language. If possible, also 1 questionnaire for your partner (or family member or other relative). We want to do an extra language screening for patients with a left-sided brain tumor. In total, this study moment will last approximately 80 (or 70) minutes.
We will also send you 5 questionnaires via email, which you can complete at home. It will take approximately 45 minutes to complete all questionnaires.

Days on the ward

You do not need to do anything on the day of admission and on the day of surgery. After the surgery you will stay in the hospital for a few days according to regular care. The number of days you will be in the hospital depends on the judgment of the doctors. This is determined according to regular care. On the days that you stay on the nursing ward, we always ask you to fill in 1 short questionnaire. We also ask you to wear a watch with which we can measure your activity and sleep quality. In addition, we would like to ask you to briefly indicate in a sleep diary how you slept. The time you spend in the hospital on the examination these days is a maximum of 10 minutes. During your hospital stay, nurses will monitor your psychiatric well-being.

Day of discharge

On the day you are allowed to go home, we would like to conduct an interview with you, we will ask you to complete 2 questionnaires and we will perform a short screening for memory, attention and language. We would also like to ask your partner (or family member or other close relative) to complete 1 questionnaire. A language screening is conducted for patients with a left-sided brain tumor (mostly according to regular care). This study moment will last approximately 55 (or 45) minutes.

After the surgery

About 2 weeks after the surgery, we would like to ask you to fill in 4 questionnaires. You can do this at home and completing all questionnaires will take approximately 30 minutes in total. According to regular care, an appointment in the hospital is scheduled around 5 to 8 weeks after surgery. On this day in the hospital, we would like to conduct the interview one more time, we ask you to fill in 2 more questionnaires and to perform a number of tests for memory, attention and language. If possible, also 1 questionnaire for your partner (or family member or other close relative). This study moment will last approximately 80 minutes.

For peer review only - http://bmjopen.bmj.com/site/about/guidelines.xhtml

Version 4, 14-02-20

About 3 months after surgery, we would like to ask you to fill in 5 questionnaires. You can do this at home and completing all the questionnaires will take approximately 45 minutes in total.

In total, the study will take about 5 to 6 hours of your time spread over 3 months.

Study moment	Where	How long
Preoperative	Outpatient clinic	70 (tot 80) minutes
	Home	45 minutes
During admission	Ward	30 minutes
Day of discharge	Ward	45 (tot 55) minutes
2 weeks postoperative	Home	30 minutes
5 – 8 weeks postoperative	Outpatient clinic	80 minutes
3 months postoperative	Home	45 minutes
**Total**		**5 – 6 hr**

For peer review only

For peer review only - http://bmjopen.bmj.com/site/about/guidelines.xhtml

Appendix D – Additional background information

Our body produces the hormone cortisol. Cortisol affects many things, such as our mood and our sleep. Cortisol is produced in the adrenal glands, which are regulated by another hormone called adrenocorticotropic hormone (ACTH). The ACTH is made in the pituitary gland, an organ under the brain. When there is enough cortisol, the pituitary gland receives a signal to stop making hormones, this way the adrenal glands no longer make cortisol.

The drug dexamethasone is very similar to cortisol that the body can make itself. But it is much stronger than the body's own cortisol. A small dose of dexamethasone can already ensure that the pituitary gland no longer makes a hormone. This causes the adrenal glands to stop making cortisol. So a side effect of treatment with dexamethasone is that our own bodies no longer make cortisol.

There is one big difference between cortisol and dexamethasone. Dexamethasone works through a single target. This is through the glucocorticoid receptor (GR). Cortisol acts on two targets: via the GR, but also via the mineralocorticoid receptor (MR). Cortisol thus activates MR and GR. Dexamethasone only activates the GR. Because our body no longer makes cortisol after dexamethasone, cortisol can no longer activate the MR. The result is that only the GR is active, while the MR remains empty. We think that the side effects after dexamethasone are because the MR is empty, because the functioning of cortisol in the brain depends on the MR.

In this study we add cortisol to the treatment with dexamethasone (as a pill, cortisol has been given the name 'hydrocortisone'). The hydrocortisone can bind to the MR. In this way we hope to get the normal functioning of the MR again. And we hope that the side effects will decrease.

During the study, you may be asked whether blood may be taken from you and stored for further examination. We want to check in the blood which type of MR you have. We can investigate this by looking at the gene of the MR in the DNA. It is known that a certain variant of the MR gene gives a lower risk of getting depression. In this study we want to see whether this can be an underlying reason for the complaints that some patients get from the dexamethasone.

We want to investigate the effect of the hydrocortisone within the sample tissue that is removed from the removed tumor tissue. We expect the MR to become active again with the added hydrocortisone. We want to measure that in this tissue.

For peer review only

For peer review only - http://bmjopen.bmj.com/site/about/guidelines.xhtml

Version 4, 14-02-20

Appendix E: Informed Consent form participant

Cortisol to prevent psychiatric adverse effects of dexamethasone

- I have read the study information letter. I was also able to ask questions. My questions have been answered to my satisfaction. I had enough time to decide whether to participate.

- I know that participation is voluntary. I know that I may decide at any time not to participate after all or to withdraw from the study. I do not need to give a reason for this.

- I give permission to request information from my general practitioner if there are any questions about your medical history or medication use.

- I know that for the purpose of auditing and monitoring the study, some people may have access to all my data. These people are mentioned in this information letter. I give permission for that access by these persons.

- I know that my partner (or family member or other relative) also has to fill in a questionnaire several times and he/she agrees to this.

- I give permission for the collection and use of my data to answer the research question in this study.

- I □ **do**
  □ **do not**
  consent for blood collection and to keep and use this for future research in the field of research described in the information letter.

- I □ **do**
  □ **do not**
  consent to keep a sample of the removed tumor tissue and to keep it and use it for future research in the field of research described in the information letter.

- I □ **do**
  □ **do not**
  consent to keep my personal data and body material longer and use it for future research in the field of research described in the information letter.

For peer review only

For peer review only - http://bmjopen.bmj.com/site/about/guidelines.xhtml

- I ◻ **do**
  ◻ **do not**
  want to be informed on the treatment I received (placebo or hydrocortisone)

- I want to participate in this study.

Name study participant: ___________________

Signature:________________________ Date : __ / __ / __

I hereby declare that I have fully informed this study participant about this study.

If information comes to light during the course of the study that could affect the study participant's consent, I will inform him/her of this in a timely fashion.

Name investigator (or its representative):_______________________________

Signature:_______________________ Date: __ / __ / __

For peer review only

For peer review only - http://bmjopen.bmj.com/site/about/guidelines.xhtml

Appendix F: Informed Consent form partner/other relative

Cortisol to prevent psychiatric adverse effects of dexamethasone

- I am aware that my partner (or family member or other close relative) is participating in the study. I was also able to ask questions. My questions have been answered to my satisfaction. I have had enough time to decide whether I want to fill in a questionnaire about my partner (or relative or other close relative) 3 times for the study.

- I know that participation is voluntary. I know that I may decide at any time not to participate after all or to withdraw from the study. I do not need to give a reason for this.

- I want to participate in this study.

Name partner (or family member or other relative):______________

Signature:_______________________ Date : __ / __ / __

I hereby declare that I have fully informed this partner (or other close relative) about this study.

If information comes to light during the course of the study that could affect the partner's (or other close relative's) consent, I will inform him/her of this in a timely fashion.

Name investigator (or its representative):______________________________

Signature:______________________ Date: __ / __ / __

For peer review only - http://bmjopen.bmj.com/site/about/guidelines.xhtml
